# Phosphorylation-Mediated Molecular Pathway Changes in Human Pituitary Neuroendocrine Tumors Identified by Quantitative Phosphoproteomics

**DOI:** 10.3390/cells10092225

**Published:** 2021-08-27

**Authors:** Jiajia Li, Siqi Wen, Biao Li, Na Li, Xianquan Zhan

**Affiliations:** 1 Key Laboratory of Cancer Proteomics of Chinese Ministry of Health, Central South University, 87 Xiangya Road, Changsha 410008, China; 198111089@csu.edu.cn (J.L.); 198111109@csu.edu.cn (S.W.); libiao6660208@163.com (B.L.); 2 Medical Science and Technology Innovation Center, Shandong First Medical University, 6699 Qingdao Road, Jinan 250117, China; qianshoulina@163.com; 3 Shandong Key Laboratory of Radiation Oncology, Shandong First Medical University, 440 Jiyan Road, Jinan 250117, China

**Keywords:** pituitary neuroendocrine tumor (PitNET), phosphorylation, phosphoprotein, phosphoproteome, phosphoproteomics, TMT, TiO_2_, liquid chromatography, tandem mass spectrometry, signaling pathway, molecular network, biomarkers, therapeutic target

## Abstract

To investigate the biological role of protein phosphorylation in human nonfunctional pituitary neuroendocrine tumors (NF-PitNETs), proteins extracted from NF-PitNET and control tissues were analyzed with tandem mass tag (TMT)-based quantitative proteomics coupled with TiO_2_ enrichment of phosphopeptides. A total of 595 differentially phosphorylated proteins (DPPs) with 1412 phosphosites were identified in NF-PitNETs compared to controls (*p* < 0.05). KEGG pathway network analysis of 595 DPPs identified nine statistically significant signaling pathways, including the spliceosome pathway, the RNA transport pathway, proteoglycans in cancer, SNARE interactions in vesicular transport, platelet activation, bacterial invasion of epithelial cells, tight junctions, vascular smooth muscle contraction, and protein processing in the endoplasmic reticulum. GO analysis revealed that these DPPs were involved in multiple cellular components (CCs), biological processes (BPs), and molecule functions (MFs). The kinase analysis of 595 DPPs identified seven kinases, including GRP78, WSTF, PKN2, PRP4, LOK, NEK1, and AMPKA1, and the substrate of these kinases could provide new ideas for seeking drug targets for NF-PitNETs. The randomly selected DPP calnexin was further confirmed with immunoprecipitation (IP) and Western blot (WB). These findings provide the first DPP profiling, phosphorylation-mediated molecular network alterations, and the key kinase profiling in NF-PitNET pathogenesis, which are a precious resource for understanding the biological roles of protein phosphorylation in NF-PitNET pathogenesis and discovering effective phosphoprotein biomarkers and therapeutic targets and drugs for the management of NF-PitNETs.

## 1. Introduction

Human genomics has made great advancements in recent years, and ca. 20,300 genes have been deciphered by means of sequencing techniques [1]. Some researchers tried to illuminate the mechanisms of an illness at the level of the genome, which does work in some diseases. However, when certain determinable factors of a disease such as cancer are obtained from only the gene level for the prediction, prevention, diagnosis, therapy, and prognostic assessment of cancer, the effort often fails. This is because many diseases, including cancers, are very complex, involving a series of molecule alterations at the genome, transcriptome, proteome, and metabolome levels, and these molecules interact mutually and function in a molecular network system [2]. On the other hand, this could be explained by proteomic variations; in other words, it could be described as a “one gene, multiple proteins” instead of a “one gene, one protein” model. Studies on omics and systems biology have revealed that the proteome is much more intricate than the genome with regard to the aspects of amount and structure [3]. The proteomic variations are generally stemmed from mutations, splicing, and post-translational modifications (PTMs) [4]. PTMs, including phosphorylation, acetylation, ubiquitylation, nitration, and glycosylation, result in proteomic variations and the diversity of protein function to a great extent. Phosphorylation is an important PTM which can transfer the conformation of amino acid residues Ser (S), Tyr (Y), and Thr (T) through adding a phosphate group (PO_4_) to the amino acid residues. Additionally, the phosphorylation proportion of residues of S, T, and Y in humans was 86.4%, 11.8%, and 1.8%, respectively [5,6,7]. The phosphorylation of a protein plays important roles in almost every conceivable behavior in an organism. Similarly, abnormal phosphorylation processes are commonly associated with the mechanism of a tumor. For example, immunohistochemistry staining analysis of 89 invasive breast cancer tissues and six normal mammary tissues found that more than 70% of invasive breast cancer tissues expressed high levels of phosphorylated PDK-1, AKT, p70S6K, and EGFR, relative to normal mammary tissues. Elevated phosphorylation levels for the proteins PDK-1, AKT, p70S6K, EGFR, and Stat3 were highly correlated with invasive breast cancers (*p* < 0.05). These phosphorylation-activated kinase pathways may act as the molecular pathogenesis of human breast cancer [8]. These studies helped researchers to uncover the mechanism of pituitary neuroendocrine tumors (PitNET) with respect to protein phosphorylation and its kinase.

PitNETs are a common kind of neuroendocrine neoplasm derived from adenohypophyseal cells, and PitNETs constitute about 15%–20% of intracranial neoplasms [9]. They can cause mortality either by exerting cerebral pressure from the pituitary bulk or by generating superfluous pituitary hormones. Depending on the serum hormone levels, PitNETs can be categorized into functional PitNETs (F-PitNETs) and nonfunctional PitNETs (NF-PitNETs) [10]. NF-PitNETs are generally benign tumors in the pituitary gland. However, they are difficult to diagnose at an early stage because there is a lack of detectable hypersecreting serum hormones and specific clinical symptoms of NF-PitNETs at the early stage compared to FPAs [11]. NF-PitNETs are commonly detected according to some non-specific clinical symptoms such as headache, vision loss, or hypopituitarism. Despite the great progress made in microsurgical and radiotherapy techniques, some NF-PitNETs remain difficult to cure. The recurrence of tumors and the development of secondary malignancies are still the main causes of mortality for NF-PitNETs [5,12]. It is necessary to obtain a comprehensive characterization of the mechanisms in NF-PitNETs. At the level of the genome, previous research has identified multiple common genetic mutations of NF-PitNETs, such as the activated mutations of GNAS, which have been found to be related to the pathogenesis of GH-PAs, while USP8 mutations were involved in 11 out of 20 ACTH-PAs [13,14,15]. At the level of RNAomics, lncRNAs and mRNAs differentially expressed in primary gonadotrophin adenomas have been identified by RNA-seq [16]. Furthermore, because of the crucial role of proteomics, it would be of great clinical significance to analyze the pathogenesis of NF-PitNETs from the perspective of phosphoproteomics and proteomics.

Phosphorylation and dephosphorylation processes proceed by virtue of enzymes, kinases and phosphatases, respectively. About 2%–5% of the human genome codes protein kinases and phosphatases [17]. Phosphokinases play an important role in the development of tumors, including NF-PitNETs, by shifting a phosphate moiety (PO_4_) to residues of Ser/Thr/Tyr of the substrate, which could induce a change in protein electricity, a conformational change of the substrate, and protein–protein interaction [17,18]. There are a huge number of studies with the purpose of finding effective medicines targeting phosphokinases. What is gratifying is that some such kinase inhibitors have been invented as drugs to target the corresponding kinases; for example, Herceptin and Gleevec are tyrosine kinase inhibitors, and some inhibitors have been developed to target serine-threonine kinases, such as p38, Rho-kinase, cyclin-dependent kinases, and Chk1 [19]. This helps researchers to find similar drugs targeting phosphokinases differentially expressed in NF-PitNETs.

The tandem mass tag (TMT) isobaric labeling technique, in combination with titanium dioxide (TiO_2_) enrichment of phosphopeptides and liquid chromatography-tandem mass spectrometry (LC-MS/MS), is an effective phosphoproteomics approach to identify amino acid sequences, phosphosites, and the phosphorylation level of a phosphoprotein [20,21,22].

This study used TMT-TiO_2_-LC-MS/MS quantitative proteomics to investigate the DPP profiling, phosphorylation-medicated signaling pathway network changes, and kinase system alterations in NF-PitNETs. These findings will be a precious resource to provide in-depth insights into the functions of the phosphoproteome in NF-PitNETs, and to help in the discovery of phosphoprotein biomarkers and effective therapeutic targets for PitNETs.

## 2. Materials and Methods

### 2.1. Tissue Specimen and Preparation of Protein Samples

Seven NF-PitNET tissue samples, obtained from the Department of Neurosurgery of Xiangya Hospital, Central South University, were approved by the Xiangya Hospital Medical Ethics Committee of Central South University. Five post mortem control pituitary tissue samples, obtained from the Memphis Regional Medical Center, were approved by the University of Tennessee Health Science Center Internal Review Board (Supplementary Table S1). Written informed consent was obtained from the family of each control post mortem pituitary subject or each patient for PitNET biopsy tissues, after full explanation of the purpose and nature of all experimental procedures.

Each tissue sample (100 mg) was treated in a volume (1 mL) of urea pyrolysis solution (9 M urea, 20 mM 2-hydroxyethyl HEPES, 2.5 mM sodium pyrophosphate, 1 mM sodium orthovanadate, 1 mM β-glycerophosphate, and pH 8.0) with an ice bath ultrasound (100 W, 10 times, each time for 10 s) and was centrifuged (18,000× *g*, 10 min, 4 °C) to obtain the supernatant as the extracted protein sample. The protein content of each sample was determined with the Bradford method (Bradford Protein Quantification Kit, YEASEN, Cat# 20202ES76). The extracted protein samples were stored at −80 °C. Quantitative phosphoproteomics was carried out with the four mixed control samples vs. the four mixed NF-PitNET samples, and immunoprecipitation and Western blot were carried out with the five mixed control samples vs. the three mixed NF-PitNET samples (Supplementary Table S1).

### 2.2. Enzyme Hydrolysis and Peptide Quantification

NF-PitNET protein samples (n = 4) were mixed equally as tumor protein samples. Post-mortem control pituitary protein samples (n = 4) were mixed equally as control protein samples. A total of 300 µg of proteins were taken from each mixed protein sample, and then dithiothreitol (DTT) was added (the final DTT concentration was 10 mM), and incubated (37 °C; 2.5 h). The iodoacetamide (IAA) was added (the final IAA concentration was 50 mM), and incubated in the dark (2.5 h). Five times the volume of water was added to dilute the solution to 1.5 M, then trypsin was added at a 1:50 ratio (*v*:*v*) to digest the protein mixture (37 °C; 18 h). The tryptic peptides were processed with an SPE C18 column (Waters WAT051910, Waters Corporation, Milford, CT, USA) for desalination, and then lyophilized.

### 2.3. TMT Labeling

An aliquot (100 μg) of tryptic peptides (NF-PitNET: n = 3 parts; control: n = 3 parts) was taken from each digested protein sample. These 6 aliquots of tryptic peptides were labeled by the TMT six plex^TM^ isobaric label reagent (Thermo Fisher Scientific, Waltham, MA, USA) with reporter ions (*m/z* = 126, 127, 128, 129, 130, and 131, respectively).

### 2.4. TiO_2_ Enrichment of Phosphopeptides

The six TMT-labeled mixed peptides (NF-PitNET: n = 3; control: n = 3) were equally mixed (1:1:1:1:1:1), lyophilized in vacuum, and resuspended in 1×DHB buffer, which was mixed with 3% DHB, 80% acetonitrile (ACN; I592230123 Merck, Darmstadt, Germany), 0.1% trifluoroacetic acid (TFA), and diluted with water at a 1:4 ratio (*v*:*v*). TiO_2_ beads were added to the redissolved mixed TMT-labeled peptide solution, and then slightly vibrated (40 min) and centrifuged (5000× *g*, 1 min). The supernatant was discarded, retaining the TiO_2_ beads with the peptides, followed by washing with 50 μL of washing buffer 1 (30% ACN and 3% TFA) (3×) and 50 μL of washing buffer 2 (80% ACN and 0.3% TFA) (3×) to remove the remaining impurities. After washing, the TiO_2_ beads containing peptides were treated with 50 μL of elution buffer (40% ACN and 15% NH_3_·H_2_O) (3×) to elute the phosphopeptides, followed by concentration in vacuum to collect the phosphopeptides. The collected phosphopeptide samples were dissolved with a volume (30 μL) of 0.1% TFA, and then 20 μL phosphopeptide samples were taken for LC-MS/MS analysis.

### 2.5. LC-MS/MS Analysis of Enriched Phosphopeptides

Each enriched phosphopeptide sample was analyzed with LC-MS/MS in a high-performance liquid chromatography (HPLC) system EASY-nLC1000 at nanoliter flow rate and Q-Exactive mass spectrometer (Thermo Finnigan, Palmer, MA, USA). The enriched phosphopeptide sample was loaded with an autosampler onto a sample column Thermo Scientific EASY column (2 cm × 100 µm 5 µm-C18) that was balanced with 95% solution A (0.1% formic acid aqueous solution), and then the enriched peptides were separated with an analytical column (75 µm × 250 mm 3 µm-C18 at a flow rate of 250 nL/min) whose linear gradient was solution B (0.1% formic acid in 84% ACN aqueous solution). The HPLC liquid phase gradients were set as follows: solution B, linear, gradient (0%–55% for 0–220 min; 55%–100% for 220–228 min; maintained at 100% for 228–240 min). LC-separated peptides were analyzed with a Q-Exactive mass spectrometer (Thermo Finnigan) for 240 min. The parameters of mass spectrometry (MS) were set as follows: scan mode positive-ion, scan range *m*/*z* 350–1800, resolution 70,000 at *m*/*z* 200, automatic gain control (AGC) target 3 × 10^6^, maximum inject time 20 ms, number of scan ranges 1, and dynamic exclusion 30.0 s. For each MS full scan, the most abundant 10 precursor ions were selected for MS/MS analysis. The MS/MS analysis parameters were set as follows: MS^2^ activation type HCD fragmentation; isolation window 2 *m*/*z*, resolution 17, 500 at *m*/*z* 200, maximum inject time 60 ms, normalized collision energy 29 eV, and underfill ratio 0.1%.

MS/MS data were input into MaxQuant software (version 1.3.0.5) for data analysis, protein identification, phosphorlylation-site determination, and quantification of phosphorlylation level. The database used for this analysis was uniprot_human_76417_20141212.fasta (76,417 entries, downloaded on 12 December 2014). The database searching parameters were set as follows: enzyme (trypsin), main search 6 ppm, max missed cleavage 4, first search 20 ppm, MS/MS tolerance 20 ppm, fixed modification carbamidomethyl (C), variable modification oxidation (M), acetyl (protein N-term) and phospho (STY), include contaminants (True, decoy), database pattern (reverse), and time window (match between runs 2 min, peptide FDR 0.01, and protein FDR 0.01). Thus, MS/MS data were used to determine the protein amino acid sequence and phosphorylation sites. TMT reporter ion intensities were used to quantify the phosphorylation level with MaxQuant software (version 1.6.1.0).

### 2.6. Statistical Analysis and Bioinformatics

The protein data files from MaxQuant were processed with GraphPad Prism to obtain the volcano plot. Those DPPs were disposed for KEGG signaling pathway-enrichment analysis with the DAVID database, and for Gene Ontology (GO) analysis including biological processes (BPs), cellular components (CCs), and molecular functions (MFs) with R software. To understand the biological roles of phosphorylations in human NF-PitNETs, the functional annotation of each DPP was analyzed in the DAVID database.

### 2.7. Immunoprecipitation and Western Blot Analyses of DPP Calnexin

Three NF-PitNET tissue samples were equally mixed as the tumor protein group, and three control pituitary tissue samples were equally mixed as the control protein group (Supplementary Table S1). The mixed tissue samples were used to extract proteins for immunoprecipitation (IP) and Western blot analysis. Briefly, each tissue was completely washed to remove blood impurities, and was triturated in liquid nitrogen, followed by extracting total proteins with buffer (0.15 M NaCl, 0.025 M Tris, 1% NP-40, 0.001 M EDTA, 1 mM PMF, and 5% glycerol). The content of total proteins was measured with a BCA protein assay kit (lot. no. B68010, CAT 2020-B, YEASEN, Shanghai, China). Calnexin was immunoprecipitated with mouse anti-human calnexin polyclonal antibody (6 μg; sc-23954, Santa Cruz Biotechnology, Santa Cruz, USA) from total proteins (NF-PitNET: n = 1.5 mg; control: n = 1.5 mg). To test the specificity of anti-calnexin antibody, a negative-control group was set, which used mouse IgG (6 μg; Cat. No. B900620, Proteintech, Wuhan, China) to replace anti-calnexin antibody for IP. The obtained IP products was divided into two parts. (i) One part of IP products was used for Western blot to test calnexin immunoreactivities with rabbit anti-human calnexin polyclonal antibody (1:10,000 dilution) (AB92573; Lot. GR53900-29; Abcam, Boston, USA). An amount (1 μg) of mouse anti-calnexin antibodies (Santa Cruz Biotechnology, sc-23954) was immunoblotted with rabbit anti-human calnexin polyclonal antibody (1:10,000 dilution) (AB92573; Lot. GR53900-29; Abcam) to test for any cross-reactions between two types of anti-calnexin antibodies. The total proteins extracted from NF-PitNETs and control pituitary tissues were immunoblotted with rabbit anti-calnexin antibody (AB92573; Lot. GR53900-29; Abcam) to test the overall level of calnexin in the tissues. (ii) Another part of IP products was used for Western blot to detect phosphoserine immunoreactivities with rabbit anti-human phosphoserine polyclonal antibody (1:1000 dilution) (bs-11993R; Lot. AI10085027; Bioss Antibodies, Beijing, China). An amount (1 μg) of anti-calnexin antibodies (Santa Cruz Biotechnology, sc-23954) was immunoblotted with anti-phosphoserine antibody (bs-11993R; Lot. AI10085027; Bioss Antibodies) to test any cross-reaction between anti-calnexin antibodies and anti-phosphoserine antibodies. The total proteins extracted from NF-PitNETs and control pituitary tissues were immunoblotted with anti-phosphoserine antibody (bs-11993R; Lot. AI10085027; Bioss Antibodies) to examine the overall level of phosphor-calnexin in tissues.

## 3. Results

### 3.1. Differentially Phosphorylated Protein (DPP) Profiling in NF-PitNETs

**Figure 1:**
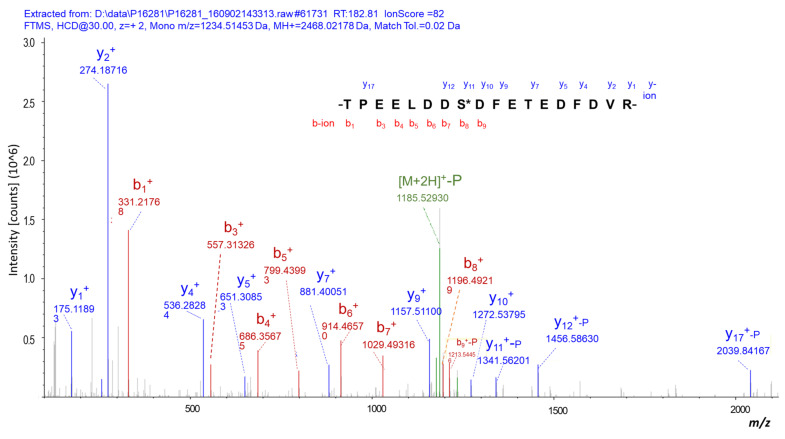
MS/MS spectrum of the representative peptide. The representative peptide ^633^TPEELDDS*DFETEDFDVR^652^ derived from catenin alpha-1 protein (P35221). The observed b- and y-ions were labeled in the MS/MS spectrum. S* = phosphorylated amino acid residue.

**Figure 2:**
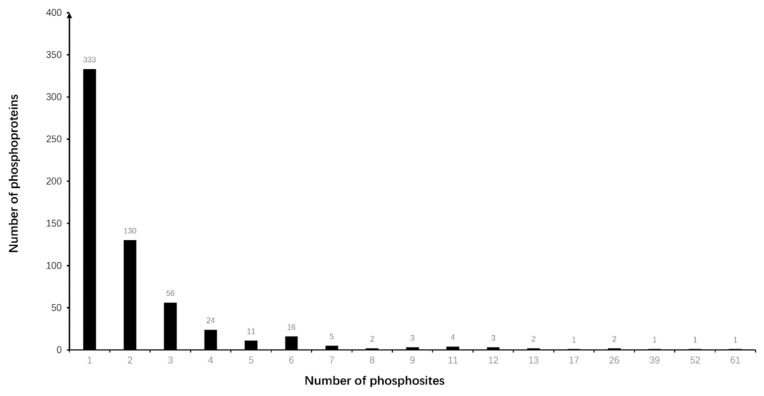
Distribution profile of phosphoproteins based on the number of phosphosites in NF-PitNETs and control pituitary tissues.

**Figure 3:**
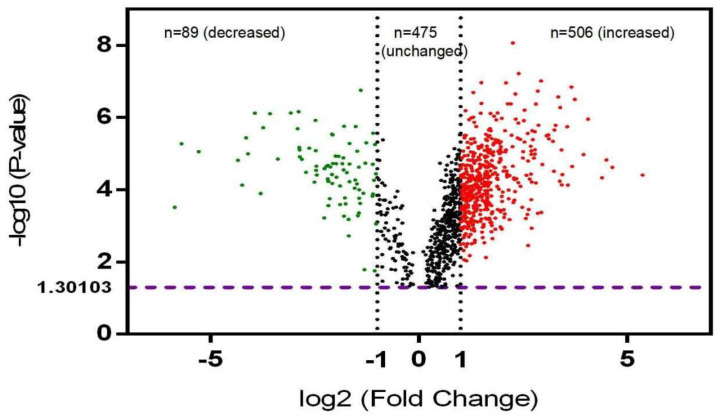
The volcano plot for 595 differentially phosphorylated proteins (DPPs) between NF-PitNETs and controls. The abscissa represents the logarithm of the expression level difference from a certain protein in NF-PitNETs and controls, namely log2(FC). The greater the absolute value of abscissa, the greater the expression with multiple differences between NF-PitNETs and controls. The y-coordinate represents the negative log of *p*-value, namely the −log10(*p*-value). The larger the ordinate value, the more significant DPPs were, and the more reliable the screened DPPs were.

In total, 595 DPPs with 1412 phosphosites were identified in NF-PitNETs compared to controls (*p* < 0.05) (Supplementary Table S2). A representative MS/MS spectrum of phosphopeptide is shown, which is phosphopeptide ^633^TPEELDDS*DFETEDFDVR^652^ ([M+H]^+^, *m*/*z* = 1234.51, z = 2+, S* = phosphorylated serine residue) derived from catenin alpha-1 (P35221) (Figure 1), with a good MS/MS spectrum, high S/N ratio, and excellent b-ion and y-ion series (b_1_, b_3_, b_4_, b_5_, b_6_, b_7_, b_8_, and b_9_; y_1_, y_2_, y_4_, y_5_, y_7_, y_9_, y_10_, y_11_, y_12_, and y_17_). The phosphorylation site was localized to the amino acid residue Ser_641_, and the phosphorylation level was significantly increased (ratio of T/N = 1.36, *p* = 1.56 × 10^−3^) in NF-PitNETs compared to controls (Supplementary Table S2). Similarly, each DPP and its phosphosites were identified and quantified. Supplementary Table S2 shows the accession number, gene name, protein name, modified peptide, phosphosite, phosphorylated probabilities, ion score, protein score, and phosphorylated levels. Among 595 DPPs with 1412 phosphosites, 333 DPPs had 1 phosphosite, 130 DPPs had 2 phosphosites, 56 DPPs had 3 phosphosites, 24 DPPs had 4 phosphosites, 11 DPPs had 5 phosphosites, 16 DPPs had 6 phosphosites, 5 DPPs had 7 phosphosites, 2 DPPs had 8 phosphosites, 3 DPPs had 9 phosphosites, 4 DPPs had 11 phosphosites, 3 DPPs had 12 phosphosites, 2 DPPs had 13 phosphosites, 1 DPP had 17 phosphosites, 2 DPPs had 26 phosphosites, 1 DPP had 39 phosphosites, 1 DPP had 52 phosphosites, and 1 DPP had 61 phosphosites (Figure 2). Among these 595 DPPs, 89 DPPs had the significantly decreased phosphorylation levels, and 506 DPPs had significantly increased phosphorylation levels (Figure 3); these 506 DPPs with increased phosphorylation levels included four DPPs (FGA, CDS2, DNM1, and SRRM1) that were only phosphorylated in NF-PitNETs but not in controls (Supplementary Table S2).

### 3.2. Functional Characteristics of DPPs in NF-PitNETs

GO analysis was used to reveal the functional characteristics of DPPs in NF-PitNETs, including BPs, CCs, and MFs. (i) For BP analysis, 595 DPPs were significantly assigned to 122 BPs, which were mainly involved in RNA processing (mRNA processing, RNA splicing, RNA export, and transcription), the regulation of subcellular organelles (Golgi vesicle, endoplasmic reticulum/ER processing, and chondriosome regulation), cell–cell interaction (cell adhesion, and cell division), and cellular reactions to specific matter (Supplementary Table S3). For example, it is well-known that mRNA splicing through the spliceosome is an important process of protein formation, and its abnormal splicing process was closely related to the occurrence and development of tumors [23]. When the proteins relating mRNA splicing are phosphorylated, abnormal mRNA splicing might occur to cause tumors. When the directed movement of substances from the ER to the Golgi is mediated by COP II vesicles, the small form of COP II-coated vesicles are released from the ER and then fuse directly with the cis-Golgi. In contrast, larger structures are shuttled along microtubules to the cis-Golgi [24]. This is an indispensable and important functional structural component of the intracellular intimal system and is the manifestation of the directional transport of intracellular substances. Thus, it is understandable that the abnormal phosphorylation involved in vesicle transport might make it difficult for drugs to reach their specific targets in PitNET patients, and thus may contribute to the development of tumors. (ii) For CC analysis, 595 DPPs were significantly assigned to 124 CCs, which were mainly associated with cytoplasm components, nucleus components, the spliceosomal complex, Golgi, and vesicles (Supplementary Table S4). For example, the spliceosomal complex, Golgi, and vesicles play a crucial role in protein synthesis. The network of interconnected vesicles and cisternal structures is located within the Golgi apparatus on the side distal to the ER, from which secretory vesicles emerge. In addition, the trans-Golgi network is important in the later stages of protein synthesis and secretion, where it is deemed to play a key role in the sorting and targeting of secreted proteins to its specific destination [25]. When the protein on Golgi or vesicles is phosphorylated, the protein may degrade and thus affect the synthesis and secretion of other proteins, which could cause the abnormal physiological function of the body. (iii) For MF analysis, 595 DPPs were significantly assigned to 52 MFs, which were mainly regarding RNA, protein binding, ATP, protein kinase, histone, and actin (Supplementary Table S5). For example, protein binding, which is referred to as interacting selectively and non-covalently with any protein and/or protein complex (a complex containing two or more proteins and potentially including other non-protein molecules), has a vital role in maintaining normal physiological function of human body. Any cadherin binding that occurs as part of the process of cell–cell adhesion is indispensable for establishing a selectively permeable barrier to diffusion through the paracellular space between neighboring cells [26]. Phosphoproteins could bind to cadherin binding, which might affect the transport activities of neighboring cells, to thus contribute to the occurrence and development of a tumor.

Further, the functional clustering analysis of all BPs, CCs, MFs, and KEGG pathways derived from 595 DPPs (569 DPPs entered into analysis) in the DAVID database identified 12 statistically significant clusters (*p* < 0.05) (Table 1). Cluster 1 was involved in cell–cell adhesion. This bioprocess refers to the assembly and the disassembly of the cell–cell junction or the arrangement of constituent parts. There are three main kinds of cell–cell adhesive junctions in mammals, including tight junctions, adhesion junctions, and gap junctions, which detect and transmit signals from neighboring cells, and adhesion between cells is mediated by specific cell adhesion molecules such as cadherin. It is closely related to the occurrence of tumors, and the adhesion ability of most tumors is reduced or lost, while the recovery of the adhesion ability can inhibit the progression of tumor [27]. Cluster 2 was involved in RNA export, which is referred to as the directed movement of protein-coding and/or non-coding RNA molecules from the nucleus to the cytoplasm, and is critical for gene expression. It is necessary that the continuous transport of RNA species with diverse size, shape and function across the nuclear pore complexes via different export receptors and related enzymes is maintained. Its importance is emphasized by the growing interest from studies with respect to the deregulation of RNA export pathways, which is closely associated with human diseases such as cancer [28]. Cluster 3 was involved in spectrin. It is a large, heterodimeric, and cytoskeletal protein which is composed of α and β subunits and typically occupies 106 contiguous amino acid sequence motifs referred as “spectrin repeats”. Spectrin is indispensable for maintaining the structure and stability of the cell. In addition, it is associated with different cell functions including cell adhesion, cell cycle and cell spreading. Abnormality in spectrin results in diverse human diseases such as hereditary hemolytic anemia, cancer, as well as others [29]. Cluster 4 was involved in nuclear chromatin and DNA binding, which means the interacting activities selectively and non-covalently with the DNA portion of a nucleosome. In addition, all ATP-dependent chromatin remodelers own a DNA translocase domain, which enables them to move along the double-stranded DNA when ATP is hydrolyzed. This is the key action that results in DNA moving through nucleosomes [30]. Cluster 5 was involved in the BAF complex and the SWI/SNF complex. The BAF complex (= mammalian SWI/SNF complex) is found in neural stem or progenitor cells and is crucial for the regulation of gene expression and differentiation [31]. Moreover, the BAF complex evolved a tremendous complexity with a huge number of subunits encoded by the gene families. In this way, the functional and developmental regulation of tissue-specific BAF begins with the combinatorial assembly of different BAF complexes, such as nBAF, npBAF, and esBAF. In addition, whole-genome sequencing uncovered the various roles of BAF complex mutations in both neurodevelopmental disorders and human malignancies [32]. Cluster 6 was involved in sister chromatid cohesion (SCC), which is the cell cycle process where the sister chromatids of a replicated chromosome are connected with each other along the entire length of the chromosome, during their formation of S phase metaphase. This cohesion cycle is thus a crucial prerequisite for chromosome segregation (SCCS). Overall, 32% of tumors destroyed genetic alterations in the SCCS process [33]. Cluster 7 was involved in membrane fusion and SNARE interactions in the vesicular transport singling pathway. This is a cellular transport process where the transported matters are moved in the membrane-bounded vesicles. Vesicles are then targeted to, and fuse with, a specific acceptor membrane. Moreover, soluble N-ethylmaleimide-sensitive factor attachment protein receptor (SNARE) proteins are the key machinery for membrane fusion. Vesicular SNAREs (v-SNAREs) interact with their specific target SNAREs (t-SNAREs) and could produce profuse SNARE complexes to regulate the process of membrane fusion [34]. Cluster 8 was involved in material transport including the intracellular transport of viruses, tRNA export from the nucleus, the regulation of glucose transport, and the regulation of cellular response to heat. Cluster 9 was involved in actin cytoskeleton organization, which is referred as a process that leads to the assembly and arrangement of constituent parts, or the disassembly of actin-based cytoskeletal structures in the cell. It is important to regulate the actin cytoskeleton organization for maintaining the normal physiological function of cell and delivering information. Cluster 10 was involved in histone ubiquitination. There is growing evidence underlining the importance of ubiquitination as a part of the histone code. Monoubiquitination, the covalent combination of a single ubiquitin molecule at the specific lysines of histone tails, has been proven to be related to the transcriptional elongation and the DNA damage response [35]. Histone ubiquitination is a core epigenomic event in shaping the chromatin landscape of malignancy and affecting how cells respond to DNA damage [36]. Cluster 11 was involved in translation initiation. The process includes the preceding formation of the peptide bond between the first two amino acids of a protein. Moreover, it includes the formation of complexes such as the ribosome, mRNA or circRNA, and an initiation complex such as the first aminoacyl-tRNA. Recent work has uncovered multiple mechanisms of translation initiation that function in cells as the functional equivalent of canonical cap-dependent translation initiation, which has important implications for cancer [37]. Canonical cap-dependent translation initiation is inhibited by diverse stresses, including hypoxia, proteotoxic stress, nutrient limitation, or genotoxic stress. Cancer cells usually rely on the alternated modes of translation initiation for protein synthesis and cell growth when they are frequently exposed to these stresses. Cancer mutations are now being discovered in components of the translation machinery and the cis-regulatory elements of mRNAs, which both regulate the translation of cancer-relevant genes [38]. Cluster 12 was involved in nucleus organization, which is referred to as a process that leads to the assembly and arrangement of constituent parts, or the disassembly of the nucleus. It is crucial to regulate the nucleus’ organization in order to maintain the normal physiological function of cells and to deliver information [39].

### 3.3. Phosphorylation-Involved Signaling Pathway Alterations in NF-PitNETs

KEGG pathway analysis of 595 DPPs with 1412 phosphosites identified nine statistically significant signaling pathways (*p* < 0.05) (Supplementary Figure S1), including the spliceosome pathway, the RNA transport pathway, proteoglycans in cancer, SNARE interactions in vesicular transport, platelet activation, bacterial invasion of epithelial cells, tight junctions, vascular smooth muscle contraction, and protein processing in the endoplasmic reticulum.

(i) The spliceosome pathway (Supplementary Figure S1.1) is one of the key steps of the “central rule” and is considered to be an important molecular basis for the complexity of eukaryotes. The spliceosome, which consists of five nucleic acid protein subcomplexes (U1, U2, U4, U5, and U6), is the most complex macromolecular machine known in cells. The spliceosome turns pre-mRNA into mRNA by mutual dynamic coordination of five subunits of the spliceosome in a series of processes. This study found that phosphorylation occurs at the spliceosome subcomplex, including phosphorylation at residues S215 (ratio of T/N = 1.44, *p* = 5.52 × 10^−5^) in Slu7 (ID: O95391), S885 and S888 (ratio of T/N = 1.78, *p* = 1.62 × 10^−3^), and S883, S885 and S888 (ratio of T/N = 1.80, *p* = 9.93 × 10^−4^) in U1-FBP11 (ID: O75400), S583 (ratio of T/N = 2.37, *p* = 1.28 × 10^−4^) and S677 (ratio of T/N = 2.94, *p* = 2.05 × 10^−5^) in U1-related-S164 (ID: P49756), Ser359 (ratio of T/N = 1.56, *p* = 3.33 × 10^−3^) in U2-SF3a (ID: Q15459), Thr244 and Thr248 (ratio of T/N = 1.93, *p* = 2.82 × 10^−3^) in U2-SF3b (ID: O75533), S155 (ratio of T/N = 1.40, *p* = 3.43 × 10^−3^) in U2-related-SPF45 (ID: Q96I25), S619 (ratio of T/N = 2.30, *p* = 8.14 × 10^−5^) in U4/U6-Prp3 (ID: O43395), S82 (ratio of T/N = 4.76, *p* = 2.22 × 10^−5^) in U4/U6/U5tn-SnRNP-associated-Sad1 (ID: Q53GS9), S527 and S529 (ratio of T/N = 1.85, *p* = 1.43 × 10^−5^) in U4/U6/U5tn-SnRNP-associated-Prp38 (ID: Q5VTL8), S225 (ratio of T/N = 1.78, *p* = 1.65 × 10^−4^) in U5-Bn2 (ID: O75643), S107 and S109 (ratio of T/N = 6.47, *p* = 1.05 × 10^−5^) in U5-Prp28 (ID: A0A024R0Z3), S216 (ratio of T/N = 2.49, *p* = 3.31 × 10^−4^), S386 and S388 (ratio of T/N = 2.26, *p* = 1.60 × 10^−4^), S710 (ratio of T/N = 3.05, *p* = 2.94 × 10^−6^), and S898 (ratio of T/N = 2.46, *p* = 9.73 × 10^−7^) in EJC/TREX-ACINUS (ID: Q9UKV3), S560 (ratio of T/N = 1.56, *p* = 1.28 × 10^−3^) in EJC/TREX-THOC (ID: Q96FV9), S6 (ratio of T/N = 3.13, *p* = 7.49 × 10^−6^) in common component-hnRNA-HNRNPA1 (ID: P09651), S233 (ratio of T/N = 1.59, *p* = 3.25 × 10^−3^), S306 (ratio of T/N = 1.80, *p* = 5.37 × 10^−3^), S253 and S260 (ratio of T/N = 0.88, *p* = 2.01 × 10^−2^) in common component-hnRNA-C1/C2 (ID: P07910), S59 (ratio of T/N = 2.76, *p* = 4.80 × 10^−4^) in common component-hnRNA-HNRNPU (ID: Q00839), S199 and S201 (ratio of T/N = 1.77, *p* = 5.39 × 10^−3^) in common component-SR-SRSF1 (ID: J3KTL2), S133 (ratio of T/N = 2.24, *p* = 9.62 × 10^−5^) in common component-SR-SRSF10 (ID: O75494), S206 and S220 (ratio of T/N = 0.36, *p* = 6.85 × 10^−5^), S206, S208 and S212 (ratio of T/N = 0.57, *p* = 7.60 × 10^−5^), and S189 and S191 (ratio of T/N = 1.54, *p* = 8.72 × 10^−5^) in common component-SR-SRSF2 (ID: Q01130), S431 (ratio of T/N = 1.71, *p* = 7.27 × 10^−4^) in common component-SR-SRSF4 (ID: Q08170), S314 and S316 (ratio of T/N = 1.89, *p* = 1.39 × 10^−4^) in common component-SR-SRSF6 (ID: Q13247), S231 and S233 (ratio of T/N = 1.27, *p* = 7.05 × 10^−3^) in common component-SR-SRSF7 (ID: Q16629), and S204 (ratio of T/N = 3.98, *p* = 2.08 × 10^−4^) in common component-SR-SRSF9 (ID: Q13242).

(ii) The RNA transport pathway (Supplementary Figure S1.2) from the nucleus to the cytoplasm is the basis of gene expression. The different RNA species that are generated in the nucleus, such as tRNAs, U snRNAs, rRNAs, and mRNAs, are processed via specific complex. For example, the exon–junction complex (EJC) and the transcription–export complex (TREX) help to export the pre-mRNAs from the nucleus to the cytoplasm [40]. Moreover, the nuclear export of mRNAs was functionally coupled with different processes in gene expression, such as transcription, splicing, 3’-end formation, and even translation. EJC and TREX also allow the later other transcriptional factors or signaling pathways to come into play, such as the mRNA surveillance pathway [41]. For these crucial complexes, their molecules were associated with the identified phosphoproteins. This study found that phosphorylation occurred at the RNA transport pathway-related subcomplex, including phosphorylation at residues S994 (ratio of T/N = 2.38, *p* = 7.92 × 10^−5^) in nuclear pore complex-(NPC-)Nup155 (ID: O75694), S612 (ratio of T/N = 1.94, *p* = 9.16 × 10^−4^), and S888 (ratio of T/N = 1.30, *p* = 6.60 × 10^−3^) in NPC-Nup98 (ID: P52948), Tyr83, S85 and S87 (ratio of T/N = 0.02, *p* = 3.05 × 10^−4^) in NPC-Rae1 (ID: L0R530), Thr437 (ratio of T/N = 1.69, *p* = 2.09 × 10^−3^) in NPC-Nup214 (ID: P35658), Thr1399 (ratio of T/N = 3.36, *p* = 3.84 × 10^−6^), and S1400 (ratio of T/N = 3.15, *p* = 4.81 × 10^−5^) in NPC-RanBP2 (ID: P49792), S28 (ratio of T/N = 1.41, *p* = 7.09 × 10^−3^) in survival motor neuron (SMN) complex-SMN (ID: Q16637), S39 (ratio of T/N = 4.61, *p* = 4.17 × 10^−7^) in translation initiation factors-(eIFs-)eIF3-EIF3CL (ID: B5ME19), S1604 (ratio of T/N = 1.44, *p* = 1.33 × 10^−3^) in eIFs-eIF3-EIF4G3 (ID: Q59GJ0), S214 (ratio of T/N = 7.28, *p* = 1.63 × 10^−6^), S164 (ratio of T/N = 3.43, *p* = 1.12 × 10^−5^), S135 and S137 (ratio of T/N = 3.26, *p* = 2.02 × 10^−5^), S107 and S113 (ratio of T/N = 4.17, *p* = 2.37 × 10^−5^), and S992 (ratio of T/N = 2.38, *p* = 7.92 × 10^−5^) in eIFs-eIF5B (ID: O60841), S497 and S498 (ratio of T/N = 9.46, *p* = 3.10 × 10^−5^), and S504 (ratio of T/N = 2.01, *p* = 1.04 × 10^−3^) in eIFs-eIF4B (ID: P23588), S544 (ratio of T/N = 1.98, *p* = 7.67 × 10^−5^ eIFs-eIF2B (ID: Q13144), SS96 and S100 (ratio of T/N = 2.24, *p* = 4.81 × 10^−6^), and S443 (ratio of T/N = 1.76, *p* = 1.69 × 10^−3^) in EJC-Pinin (ID: Q9H307), S216 (ratio of T/N = 2.49, *p* = 3.31 × 10^−4^), S386 and S388 (ratio of T/N = 2.66, *p* = 1.60 × 10^−^^4^), S710 (ratio of T/N = 3.05, *p* = 2.94 × 10^−6^), and S898 (ratio of T/N = 2.46, *p* = 9.73 × 10^−7^) in EJC-ACIN1 (ID: Q9UKV3), S155 and S157 (ratio of T/N = 0.89, *p* = 1.20 × 10^−2^) in EJC-RNPS1 (ID: Q15287), S560 (ratio of T/N = 1.56, *p* = 1.28 × 10^−3^) in TREX-THOC1 (ID: Q96FV9), T220 (ratio of T/N = 2.30, *p* = 1.14 × 10^−6^), S389, S391 and S393 (ratio of T/N = 2.29, *p* = 2.02 × 10^−3^), S402 and T406 (ratio of T/N = 3.01, *p* = 1.16 × 10^−5^), S450 and S452 (ratio of T/N = 2.21, *p* = 7.88 × 10^−5^), S463 and S465 (ratio of T/N = 2.20, *p* = 2.18 × 10^−4^), S552 (ratio of T/N = 5.24, *p* = 6.10 × 10^−8^), S563 and S565 (ratio of T/N = 2.68, *p* = 1.69 × 10^−4^), S574 and S576 (ratio of T/N = 2.36, *p* = 8.98 × 10^−6^), T586 and T588 (ratio of T/N = 3.11, *p* = 3.37 × 10^−5^), T595 and S597 (ratio of T/N = 2.82, *p* = 6.37 × 10^−4^), T628 and S630 (ratio of T/N = 3.07, *p* = 3.09 × 10^−5^), S640 and S642 (ratio of T/N = 1.88, *p* = 5.12 × 10^−4^), S650 and S652 (ratio of T/N = 2.42, *p* = 4.01 × 10^−5^), S752 and S754 (ratio of T/N = 2.21, *p* = 3.17 × 10^−5^), S619 and S621 (ratio of T/N = 3.26, *p* = 2.05 × 10^−6^), S727 and S729 (ratio of T/N = 2.97, *p* = 8.16 × 10^−6^), S752 and S754 (ratio of T/N = 2.21, *p* = 3.17 × 10^−5^), S766, S768 and S770 (ratio of T/N = 1.67, *p* = 6.00 × 10^−4^), S783 and S787 (ratio of T/N = 1.48, *p* = 4.60 × 10^−3^), and T886 and S888 (ratio of T/N = 2.82, *p* = 1.08 × 10^−7^) in EJC-SRm160 (ID: A0A0S2Z4Z6).

(iii) Proteoglycans (PGs) in cancer (Supplementary Figure S1.3), including four main types: hyaluronan (HA), chondroitin sulfate proteoglycans (CSPGs), heparan sulfate proteoglycans (HSPGs), and keratan sulfate proteoglycans (KSPGs), were the key bio-molecules in multifarious biological reactions of cancer, such as proliferation, metastasis, adhesion, and angiogenesis [42]. HA, with the interaction of CD44, contributed to the acceleration of tumor growth and metastases. On the contrary, some proteolgycans, such as decorin and lumican, could act as tumor suppressors by interacting with key proteins and various receptors [42]. This study found that phosphorylation occurred at the proteoglycans in cancer-related molecules, including phosphorylations at residues S2384 (ratio of T/N = 2.66, *p* = 7.67 × 10^−5^) in CD44 and FN (ID: P02751), S781 (ratio of T/N = 3.58, *p* = 4.13 × 10^−6^), T961 (ratio of T/N = 1.61, *p* = 4.31 × 10^−4^), S1428 (ratio of T/N = 1.72, *p* = 1.74 × 10^−5^), and S1607 (ratio of T/N = 0.76, *p* = 3.19 × 10^−3^) in anyrin-ANK1 (ID: P16157), S1461 (ratio of T/N = 0.90, *p* = 5.52 × 10^−3^) in anyrin-ANK2 (ID: Q01484), S1445 (ratio of T/N = 0.90, *p* = 5.52 × 10^−3^) in anyrin-ANK3 (ID: Q12955), S309 (ratio of T/N = 2.94, *p* = 9.07 × 10^−4^) in LARG (ID: Q9NZN5), T401 and S405 (ratio of T/N = 2.15, *p* = 4.43E-04), S417 (ratio of T/N = 3.45, *p* = 5.65 × 10^−6^), and S418 (ratio of T/N = 2.14, *p* = 3.63 × 10^−6^) in cortactin (ID: Q14247), S446 (ratio of T/N = 1.33, *p* = 3.82 × 10^−2^), S447 (ratio of T/N = 1.35, *p* = 2.09 × 10^−3^), S729 (ratio of T/N = 1.69, *p* = 5.94 × 10^−4^), and S732 (ratio of T/N = 2.00, *p* = 2.53 × 10^−4^) in Raf-1 (ID: P15056), S1687 (ratio of T/N = 0.67, *p* = 2.20 × 10^−3^) in IP3R (ID: Q14571), S703 (ratio of T/N = 2.52, *p* = 2.91 × 10^−3^) in NHE-1 (ID: P19634), S497 and S498 (ratio of T/N = 9.46, *p* = 3.10 × 10^−5^), and S504 (ratio of T/N = 2.01, *p* = 1.04 × 10^−3^) in eIF4B (ID: P23588), S457 (ratio of T/N = 6.12, *p* = 1.59 × 10^−5^), and Thr93 (ratio of T/N = 6.12, *p* = 1.59 × 10^−5^) in PDCD4 (ID: Q53EL6), S2152 (ratio of T/N = 1.54, *p* = 3.04 × 10^−3^) in Filamin (ID: P21333), T245 (ratio of T/N = 0.38, *p* = 2.37 × 10^−4^), and T202 (ratio of T/N = 0.29, *p* = 5.36 × 10^−4^) in PKA (ID: A0A0A0MS54), S226 (ratio of T/N = 1.85, *p* = 1.02 × 10^−4^) in PKC (ID: P17252), and S334 (ratio of T/N = 2.54, *p* = 2.26 × 10^−4^) in SOS (ID: Q07889).

(iv) SNARE interactions in vesicular transport (Supplementary Figure S1.5) are important processes in protein translocation, and the so-called SNARE model explains the fusion mechanism in vesicular transport at the molecular level. This hypothesis suggests that each type of transport vesicle has different v-SNARE proteins (VAMP-2 in the neuron), which can be identified and bond to specific a t-SNARE (SNAP-25 in the neuron) on the corresponding target membrane, and the vesicle is anchored to the target membrane via this specific interaction to form a trans-SNARE complex. Then, with the assistance of α-nap, the trans-SNARE complex is reversibly dissociated by the ATPase of NSF to drive the membrane fusion. SNARE proteins still occupy a central position in all intracellular transport pathways studied to date [43]. Syntaxin, SNAP25, and VAMP/synaptobrevin were the first discovered SNARE proteins and are the most well-studied SNARE proteins [44]. This study found phosphorylation occurred at the SNARE interactions in vesicular transport-related molecules, including phosphorylation at residues S110 (ratio of T/N = 2.13, *p* = 6.07) in Stx1-4-STX1A (ID: Q16623), S110 (ratio of T/N = 2.13, *p* = 6.07 × 10^−4^) in SNAP23 (ID: O00161), S75 (ratio of T/N = 2.10, *p* = 5.40 × 10^−4^) in VAMP1-3 (ID: P63027), S30 (ratio of T/N = 6.31, *p* = 5.01 × 10^−5^) in VAMP4 (ID: O75379), and S137 (ratio of T/N = 5.06, *p* = 4.97 × 10^−5^), and T140 (ratio of T/N = 5.57, *p* = 3.04 × 10^−4^) in Sec22 (ID: O75396). These significantly expressed phosphoproteins and phosphosites involved in SNARE interactions in vesicular transport, especially VAMP, would contribute to the treatment of diseases caused by abnormal vesicle transport including PitNETs.

(v) Platelet activation (Supplementary Figure S1.7) platelets play a pivotal and beneficial role in primary hemostasis when the integrity of the vessel wall is destroyed. Platelet activation and adhesion at the injury sites of vascular wall is initiated via adhering to the adhesive macromolecules, such as von Willebrand factor (vWF) and collagen, or via soluble platelet agonists, such as thrombin, ADP and thromboxane A2. Various receptors are stimulated by diverse agonists, and most of them are involved in the concentration of increased intracellular Ca^2+^, which stimulates platelet shape alteration and granule secretion, to ultimately induce the “inside-out” signaling process which could induce activation of the ligand-binding function of integrin αIIbβ3 [45,46]. In addition, the binding of αIIbβ3 to its specific ligands, mainly fibrinogen, regulates the adhesion and aggregation of platelet and triggers “outside-in” signaling, which could lead to platelet spreading, additional granule secretion, adhesion and aggregation of platelet, and clot retraction [45]. This study found that phosphorylation occurred at the platelet activation-related molecules, including phosphorylation at residues S629 and S632 (ratio of T/N = 13.31, *p* = 3.17 × 10^−7^) in GP1bα (ID: P07359), T193 (ratio of T/N = 4.99, *p* = 1.10 × 10^−6^), and S191 (ratio of T/N = 9.48, *p* = 2.20 × 10^−6^) in GP1bβ (ID: P13224), S110 (ratio of T/N = 1.16, *p* = 1.17 × 10^−2^) in SNAP23 (ID: O00161), S13 (ratio of T/N = 2.83, *p* = 5.85 × 10^−6^), and S11 (ratio of T/N = 2.71, *p* = 3.38 × 10^−5^) in Lyn (ID: P07948), S995 (ratio of T/N = 2.77, *p* = 2.32 × 10^−5^) in Gs (ID: Q5JWF2), S309 (ratio of T/N = 2.94, *p* = 9.07 × 10^−4^) in Rho-GEF (ID: Q9NZN5), S1683 (ratio of T/N = 0.67, *p* = 2.20 × 10^−3^) in IP3R (ID: Q14571), T245 (ratio of T/N = 0.38, *p* = 2.37 × 10^−4^) and T202 (ratio of T/N = 0.29, *p* = 5.36 × 10^−4^) in PKA (ID: A0A0A0MS54), T1843 (ratio of T/N = 1.16, *p* = 1.17 × 10^−2^) in Talin (ID: Q9Y4G6), S173 (ratio of T/N = 3.09, *p* = 6.48 × 10^−4^) in αIIbβ3 (ID: P02675), and S299 (ratio of T/N = 7.61, *p* = 9.68 × 10^−8^), S551 (ratio of T/N = 7.21, *p* = 1.86 × 10^−7^), S291 (ratio of T/N = 5.77, *p* = 1.86 × 10^−7^), S294 (ratio of T/N = 16.67, *p* = 1.10 × 10^−6^), S304 (ratio of T/N = 8.35, *p* = 3.18 × 10^−6^), S364 (ratio of T/N = 6.64, *p* = 4.09 × 10^−6^), S297 (ratio of T/N = 9.29, *p* = 4.52 × 10^−6^), S524 (ratio of T/N = 3.90, *p* = 6.46 × 10^−6^), S594 (ratio of T/N = 4.26, *p* = 7.90 × 10^−6^), S421 (ratio of T/N = 1.94, *p* = 1.03 × 10^−5^), S588 (ratio of T/N = 22.62, *p* = 1.50 × 10^−5^), S300 (ratio of T/N = 24.92, *p* = 2.39 × 10^−5^), T595 (ratio of T/N = 10.40, *p* = 5.75 × 10^−5^), T587 (ratio of T/N = 6.97, *p* = 6.50 × 10^−5^), T359 and T364 (ratio of T/N = 3.03, *p* = 7.53 × 10^−5^), S281 (ratio of T/N = 4.03, *p* = 1.16 × 10^−4^), S485 (ratio of T/N = 2.64, *p* = 1.95 × 10^−4^), T412 (ratio of T/N = 3.12, *p* = 1.95 × 10^−4^), T499 (ratio of T/N = 2.64, *p* = 2.35 × 10^−4^), S356 and S364 (ratio of T/N = 1.98, *p* = 3.15 × 10^−4^), T275 (ratio of T/N = 4.11, *p* = 5.85 × 10^−4^), S274 (ratio of T/N = 2.03, *p* = 7.09 × 10^−4^), and S489 (ratio of T/N = 1.64, *p* = 1.46 × 10^−2^) in FG (ID: P02671). These phosphoproteins and phosphosites involved in the platelet activation pathway would expand the research data for the pathogenesis of NF-PitNETs.

(vi) For bacterial invasion of epithelial cells (Supplementary Figure S1.8), a huge number of pathogenic bacteria can invade phagocytic and non-phagocytic cells and colonize them intracellularly, and then disseminate to other cells. There are two mechanisms, referred to as the zipper model and the trigger model, through which invasive bacteria induce their own uptake by non-phagocytic host cells (e.g., epithelial cells) [47]. For example, Listeria, Staphylococcus, Streptococcus, and Yersinia enter the host cells via the zipper model. These bacteria express proteins on their surfaces that bind with cellular receptors, inducing signal cascades that lead to the tight closure of the cellular membrane around the entering bacteria. Shigella and Salmonella enter host cells via the trigger model [48]. These bacteria inject protein effectors that interact with the actin cytoskeleton by virtue of type III secretion systems. This study found that phosphorylation occurred at the bacterial invasion of epithelial cell-related molecules, including phosphorylation at residues S640 (ratio of T/N = 1.43, *p* = 1.76 × 10^−3^) in α-catenin-CTNNA2 (ID: P26232), T658 (ratio of T/N = 1.67, *p* =4.18 × 10^−4^), S655 (ratio of T/N = 1.91, *p* = 4.30 × 10^−4^), S641 (ratio of T/N = 1.36, *p* = 1.56 × 10^−3^), and S652, Thr654 and S655 (ratio of T/N = 1.35, *p* = 1.19 × 10^−2^) in α-catenin-CTNNA1 (ID: P35221), S774, Thr776, S777 and S778 (ratio of T/N = 20.95, *p* = 4.66 × 10^−5^) in dynamin-DNM1 (ID: Q05193), S774 and S778 (ratio of T/N = 3.13, *p* = 1.76 × 10^−4^) in dynamin-DNM3 (ID: Q9UQ16), S302 and S303 (ratio of T/N = 1.43, *p* = 2.16 × 10^−2^), and S322 (ratio of T/N = 1.88, *p* = 4.60 × 10^−2^) in paxillin (ID: P49023), S2384 (ratio of T/N = 2.66, *p* = 7.67 × 10^−5^) in fibronectin 1 (ID: P02751), and S290 (ratio of T/N = 0.80, *p* = 1.75 × 10^−2^) in vinculin (ID: P18206).

(vii) Tight junctions (TJs) (Supplementary Figure S1.9) are indispensable for establishing a selectively permeable barrier to facilitate diffusion through the paracellular space between neighboring cells. TJs are composed of at least three types of transmembrane proteins—occluding, claudin and junctional adhesion molecules (JAMs)—and a cytoplasmic ‘plaque’ consisting of many different proteins that form large complexes. These are proposed to be involved in junction assembly, barrier regulation, cell polarity, gene transcription, and other pathways [49,50]. This study found that phosphorylation occurred at the TJ-related molecules, including phosphorylation at residues S300 (ratio of T/N = 1.51, *p* = 3.86 × 10^−3^) in TJAP1 (ID: Q5JTD0), T710 (ratio of T/N = 0.48, *p* = 1.76 × 10^−2^) in PKCɛ (ID: Q02156), S1275 (ratio of T/N = 6.89, *p* = 2.48 × 10^−6^), S1173 and S1181 (ratio of T/N = 1.44, *p* = 3.15 × 10^−3^), and S1182 (ratio of T/N = 1.44, *p* = 3.15 × 10^−3^) in afdin (ID: P55196), S418 (ratio of T/N = 2.14, *p* = 3.63 × 10^−6^), S417(ratio of T/N = 3.45, *p* = 5.65 × 10^−6^), and S401 and Thr405 (ratio of T/N = 2.15, *p* = 4.43 × 10^−4^) in myosinⅡ-MYH10 (ID: P35580), S1954 (ratio of T/N = 1.96, *p* = 6.23 × 10^−5^), and S1943 (ratio of T/N = 3.95, *p* = 9.04 × 10^−6^) in myosinⅡ-MYH11 (ID: P35749), S1717 (ratio of T/N = 1.22, *p* = 1.23 × 10^−2^) in myosinⅡ-MYH9 (ID: P35579), S986 (ratio of T/N = 3.07, *p* = 3.01 × 10^−4^) in ZO-1/2 (ID: Q9UDY2), and S417 (ratio of T/N = 3.45, *p* = 5.65 × 10^−6^), S418 (ratio of T/N = 2.14, *p* = 3.63 × 10^−6^), and T401 and S405 (ratio of T/N = 2.15, *p* = 4.43 × 10^−4^) in cortactin (ID: Q14247).

(viii) For vascular smooth muscle (VSMC) contraction (Supplementary Figure S1.10), the VSMC is a kind of highly differentiated cell whose principal function is contraction. For contraction, VSMCs are shortened to regulate the blood flow and pressure by means of decreasing the diameter of a blood vessel. The principal mechanisms of regulating the contractile state of VSMCs are the variation in cytosolic Ca^2+^ concentration ([Ca^2+^]c). Ca^2+^ is mobilized from intracellular stores and/or the extracellular place to increase [Ca^2+^]c in VSMCs in response to the stimuli of vasoconstrictor. The increase in [Ca^2+^]c, in turn, activates the Ca^2+^-CaM-MLCK pathway and induces MLC20 phosphorylation which results in myosin–actin interactions and the development of contractile force [51]. During receptor stimulation, the contractile force is greatly enhanced by the inhibition of myosin phosphatase. Rho/Rho kinase, arachidonic acid, PKA, and PKC have been proven to play a key role in this enhancement. This study found that phosphorylation occurred at the VSMC contraction-related molecules, including phosphorylation at residues S995 (ratio of T/N = 2.77, *p* = 2.32 × 10^−5^) in Gs (ID: Q5JWF2), S663 and T668 (ratio of T/N = 2.32, *p* = 4.24 × 10^−4^), S1295 (ratio of T/N = 1.45, *p* = 1.11 × 10^−3^), S1155 (ratio of T/N = 1.81, *p* = 1.02 × 10^−2^), and S309 (ratio of T/N = 2.94, *p* = 9.07 × 10^−4^) in RhoGEF-ARHGEF11 (ID: O15085), T202 (ratio of T/N = 0.29, *p* = 5.36 × 10^−4^) in PKA (ID: A0A0A0MS54), T710 (ratio of T/N = 0.48, *p* = 1.76 × 10^−2^) in PKC-PRKCE (ID: Q02156), S226 (ratio of T/N = 1.85, *p* = 1.02 × 10^−4^) in PKC-PRKCA (ID: P17252), S732 (ratio of T/N = 2.00, *p* = 2.53 × 10^−4^), S729 (ratio of T/N = 1.69, *p* = 5.94 × 10^−4^), S447 (ratio of T/N = 1.35, *p* = 2.09 × 10^−3^), and S995 (ratio of T/N = 1.33, *p* = 3.82 × 10^−2^) in Raf (ID: P15056), and S1687 (ratio of T/N = 0.67, *p* = 2.20 × 10^−3^) in IP3R (ID: Q14571), and S196 (ratio of T/N = 1.82, *p* = 3.82 × 10^−3^) in CaD (ID: E7EX44).

(ix) Protein processing in the endoplasmic reticulum (ER) (Supplementary Figure S1.11). The ER is a crucial subcellular organelle where proteins are folded with the assistance of lumenal chaperones. Newly synthesized peptides enter the ER via the sec61 and sec62/63 pore. Correctly folded proteins are then packaged into transport vesicles that transport them to the Golgi complex. In contrast, misfolded proteins are still retained within the ER lumen in complex with molecular chaperones. The misfolded proteins are terminally bound to BiP and directed toward degradation via the proteasome in a process that is called ER-associated degradation (ERAD). The accumulation of misfolded proteins in the ER led to ER stress and mobilized a signaling pathway that is called the unfolded protein response (UPR) [52]. This study found that phosphorylation occurred at the protein processing in endoplasmic reticulum-related molecules, including phosphorylation at residues S479 and S480 (ratio of T/N = 1.67, *p* = 2.32 × 10^−4^) in HSP40 (ID: Q96KC8), S114 (ratio of T/N = 0.81, *p* = 8.68 × 10^−3^) in Ubx (ID: Q9UNZ2), S532 (ratio of T/N = 1.67, *p* = 8.68 × 10^−3^) in Sec31(ID: O94979), T375 (ratio of T/N = 6.50, *p* = 1.16 × 10^−3^) in Sec62 (ID: Q99442), S19 and S23 (ratio of T/N = 1.38, *p* = 7.29 × 10^−3^), and S498 and S499 (ratio of T/N = 3.53, *p* = 2.48 × 10^−5^) in OSTs (ID: Q8TCJ2), S583 (ratio of T/N = 10.48, *p* = 5.32 × 10^−7^), S554 and S564 (ratio of T/N = 10.67, *p* = 7.88 × 10^−6^), S564 (ratio of T/N = 6.88, *p* = 1.03 × 10^−5^), and T554 and T562 (ratio of T/N = 15.4, *p* = 7.88 × 10^−6^) in CNX (ID: P27824), S255 and S261(ratio of T/N = 2.09, *p* = 3.73 × 10^−5^), and S226 (ratio of T/N = 1.40, *p* = 1.65 × 10^−3^) in Hsp90 (ID: P08238), S428 (ratio of T/N = 3.15, *p* = 6.12 × 10^−5^) in PDIs (ID: Q15084), and S105 (ratio of T/N = 5.63, *p* = 1.33 × 10^−5^) in TRAP (ID: Q9UNL2).

### 3.4. Upstream Kinase Profiling Analysis of DPPs in NF-PitNETs

**Figure 4:**
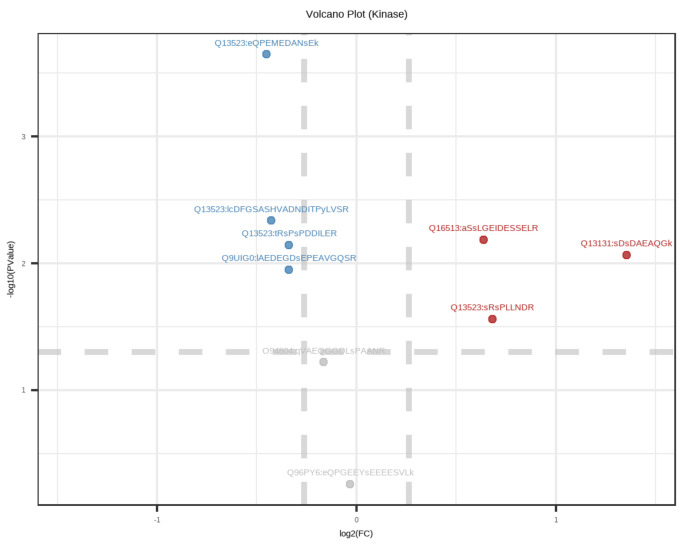
Kinases identified with phosphoproteins.

The human genome has been documented as encoding 518 protein kinases by means of transferring a phosphate-group from ATP to serine, threonine, as well as tyrosine residues [18]. The majority of these kinases are involved in human cancer initiation and progression. In this study, the PhosphoSitePlus database was used to analyze the kinases for the identified phosphoproteins in NF-PitNETs and controls, which identified seven kinases, including GRP78, WSTF, PKN2, PRP4, LOK, NEK1, and AMPKA1 (Table 2; Figure 4). Of them, the substrates of kinases PKN2 and AMPKA1 were DPPs (Supplementary Table S2; Table 2). The recent advancements in small-molecule kinase inhibitors for the treatment of various types of cancer have proven particularly successful in clinic. Moreover, protein kinases are the second largest target destination of drugs after the G-protein coupled receptors [53]. For example, PKN2 is a PKC-related serine/threonine-protein kinase and it is related to tumor cell migration, invasion and apoptosis [54]. For AMPKA1, the protein is the subunit of the 5′-prime-AMP-activated protein kinase (AMPK), which belongs to the ser/thr protein kinase family. AMPK is a cellular energy sensor in eukaryotic cells whose activity is activated by the stimuli and could increase the cellular AMP/ATP ratio. AMPK mediates the activities of numerous key metabolic enzymes by means of phosphorylation. It protects cells from stresses which induce ATP depletion by means of switching off ATP-consuming biosynthetic pathways [55,56]. Therefore, these kinases and their substrates might represent new drug targets for NF-PitNETs and provide new ways of thinking to investigate pathogenesis and therapeutic approaches in NF-PitNETs.

### 3.5. Verification of DPPs in NF-PitNETs Compared to Controls

**Figure 5:**
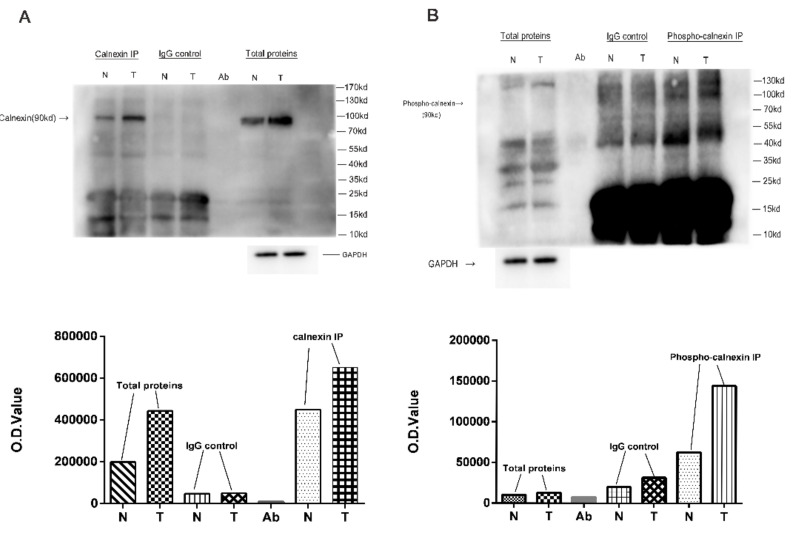
Semiquantitative analysis of phosphorylated calnexin between NF-PitNETs and controls. The total proteins were extracted from NF-PitNETs (T) and control pituitary tissues (N). Calnexin was immunoprecipitated from the total proteins (T: n = 1.5 mg; N: n = 1.5 mg) with anti-calnexin antibodies (6 μg). For the negative control experiment to test the specificity of anti-calnexin antibodies, IgG (6 μg) was used to replace anti-calnexin antibodies for immunoprecipitation. (**A**). Half of the IP product was used to detect the expression level of calnexin in NF-PitNETs (T) and control pituitary tissues (N) with another different anti-calnexin antibody. A portion of anti-calnexin antibodies (Ab: n = 1 μg) and total proteins (N: n = 20 μg; T: n = 20 μg) were used as the control to immunoblot with another different anti-calnexin antibody. (**B**). Half of the IP product was used to detect the phosphorylation level of calnexin in NF-PitNETs (T) and control pituitary tissues (N) with anti-phosphoserine antibodies. A portion of anti-calnexin antibodies (Ab: n = 1 μg) and total proteins (N: n = 20 μg; T: n = 20 μg) were used as control to immunoblot with anti-phosphoserine antibody.

Among 595 DPPs in NF-PitNETs relative to control pituitary tissues, calnexin was randomly selected to confirm the difference in its phosphorylation between NF-PitNETs and controls, which was identified with quantitative phosphoproteomics, and four phosphorylation sites at residues S554 (ratio of T/N = 10.67, *p* = 7.88 × 10^−6^), T562 (ratio of T/N = 15.45, *p* = 1.07 × 10^−5^), S564 (ratio of T/N = 6.88, *p* = 1.03 × 10^−5^), and S583 (ratio of T/N = 10.48, *p* = 5.32 × 10^−7^) were identified in calnexin with quantitative proteomics (Supplementary Table S2). The IP coupled with WB analysis showed that calnexin was overexpressed in NF-PITNETs relative to the control pituitary tissues (Figure 5A), and that the overall phosphorylation level of calnexin was higher in NF-PitNETs than controls (Figure 5B). These results are consistent with the results of MS/MS-based quantitative phosphoproteomics.

## 4. Discussion

Phosphorylation is one of the most common PTMs, and is involved in multiple complexes and crucial signaling pathways in eukaryotes, and it regulates a wide range of basic cellular processes, including cell division, growth, and differentiation. About one third of proteins are phosphorylated during the cell life cycle [18]. A single site or several sites in the same protein can be phosphorylated simultaneously. Similarly, multiple proteins can be phosphorylated by a single protein kinase, and several protein kinases can phosphorylate the same protein [17,18]. Because of that character of phosphorylation, a highly complex but well-aligned cell programming was commenced in response to a specific stimulus. Moreover, multiple sites can be phosphorylated at key regulatory proteins according to recent proteomics research [57]. In addition, there is not only one type of PTM at the same protein; instead multiple PTMs are involved simultaneously. For example, β-catenin is degraded with the help of the ubiquitin/proteosome system, and it was also regulated by αPKC-like enzyme and glycogen synthase kinase3 (GSK3β) [58]. The hydroxyl group of the beta3 integrin family is modified by the phosphate group, and in addition, N-acetylglucosamine plays a crucial role in antagonistic interaction at Thr758 [59]. The abnormal phosphorylation (gain or loss) in a protein is closely related to tumorigenesis and biological processes, such as cancer cell cycle, tumor angiogenesis, gene transcription, energy metabolism, and cell proliferation, which could be the cause of malignancies. There are a large number of studies demonstrating the role of chaotic phosphorylation in the manifestation of tumors, including PitNETs [60,61,62]. Previous research has demonstrated that FLNA, an actin cross-linking protein, is the substrate of different phospho-kinases, and that it might prevent somatostatin receptor 2 (SST2) from regulating in GH-secreting pituitary tumor by means of being promoted by cAMP pathway and inhibited by somatostatin analogs (SSA) [63].

Some previous studies have shown that phosphoproteomics analysis is carried out for PitNET and pituitary control tissues, and has identified a total of 28 phosphoproteins from two different studies: (1) six diverse pituitary phosphoproteins were identified [60], including GH, chromogranin A (CGA), secretogranin I, P1 and/or P2, 60S ribosomal protein, DnaJ homolog subfamily C member 5, and galanin, meanwhile there were eight phosphorylation sites characterized. The proteomics experimental approach included the IMAC enrichment method, which was combined with an optimized detection of phosphopeptides based on liquid chromatography-tandem mass spectrometry (LC-MS/MS) from unseparated tryptic digested human pituitary protein mixture [60]; (2) a total of 50 phosphorylation sites were characterized in 26 proteins (73 phosphopeptides) [61]. The application of experimental strategy of the in-gel IEF-LC-MS/MS methodology from digested pituitary protein mixture involves protein separation by in-gel IEF, followed by sectioning of isoelectric focusing of the proteins in a conventional immobilized pH gradient (IPG) strip, digestion of the proteins in each gel section, enrichment by IMAC for phosphopeptides, and LC-MS/MS analysis, and its data were used to identify phosphopeptide sequences and phosphorylation sites [61]. (3) Four significant molecular-network systems, including PI3K/AKT, mTOR, Wnt, and ERK/MAPK pathway-systems, and 19 hub-molecules whose expression-patterns were altered and phosphorylated, were involved in NF-PitNETs by means of PTMScan experiment-based phosphorylation analysis [62]. In addition, through the method of immunoaffinity enrichment and LC-MS/MS, the significantly down-regulated expression of PRAS40 and up-regulated phosphorylation levels of p-PRAS40 (Thr246) were related to mTOR pathway in NF-PitNETs compared to controls; meanwhile, the down-regulated protein expression of GSK-3α and GSK-3β, and the up-regulated phosphorylation levels of p-GSK3α (Ser21) and p-GSK3β (Ser9),along with the increased expression of β-catenin, were involved in the Wnt pathway in NF-PitNETs compared to controls; (4) a total of 1035 phosphoproteins with 2982 phosphorylation sites were identified in NF-PitNET tissue samples with 6-plex TMT labeling reagents along with TiO_2_ enrichment of phosphopeptides and LC-MS/MS technique. These phosphoproteins are involved in 31 statistically significant signaling pathways, including the RNA transport, spliceosome, and mammalian/mechanistic target of rapamycin (mTOR) signaling pathways, platelet activation, endocytosis, SNARE interactions in vesicular transport, vascular smooth muscle contraction, the insulin signaling pathway, proteoglycans in cancer, the cyclic guanosine monophosphate (cGMP)-protein kinase G (PKG) signaling pathway, the glucagon signaling pathway, focal adhesion, progesterone-mediated oocyte maturation, the estrogen signaling pathway, the protein processing in endoplasmic reticulum, gonadotropin-releasing hormone (GnRH) signaling pathway, gap junctions, the mitogen-activated protein kinase (MAPK) signaling pathway, and the mRNA surveillance pathway.

To gain a deeper understanding of the phosphoproteomic profile in PirNETs, TMT-TiO_2_-LC-MS/MS was used to identify phosphoproteins, phosphorylation sites, and phosporylation level in NF-PitNET tissues relative to controls in this study. A total of 595 DPPs were identified in this study, which is a precious resource in obtaining an in-depth understanding of the mechanism of NF-PitNETs and in discovering new biomarkers and therapeutic targets for the treatment of NF-PitNETs. These findings vastly expand the human pituitary phosphoprotein database to uncover PitNETs’ mechanism from phosphoproteins or phosphosites, providing an in-depth understanding of the biological significance of phosphorylation in PitNETs and allowing us to discover effective biomarkers for patients.

### 4.1. Phosphorylation-Mediated Biological Processes in NF-PitNETs

A total of 11 statistically significant pathways (*p* < 0.05) were identified in this study, which are related closely to the occurrence of cancer, and they could indicate the potential abnormal molecular mechanisms in NF-PitNETs. The spliceosome, a protein-directed metalloribozyme, not only plays a crucial role in normal biological processes, but is also involved in the mechanisms of cancer via the mutations in splicing-regulatory factors or alteration in components of the splicing machinery. Splicing includes various protein–protein and protein–RNA interactions, which are directed by a wide range of trans-acting proteins, and the process is subjected to the regulation by PTMs and protein–RNA interactions [23]. The splicing machinery is accurate and flexible because of the highly dynamic and veracious nature of the spliceosome [64]. Thus, any abnormal changes such as phosphorylation at the spliceosome could result in diseases such as cancer. Previous research attested that the alteration in SRSF3 expression in B-acute lymphoblastic leukemia (B-ALL) cells would influence the splicing process of CD19, and in return it could lead to the impaired recognition of anti-CD19 chimeric antigen receptor (CAR) in T-cells [65]. Furthermore, another study demonstrated that the splicing machinery was dysregulated in PitNETs and was associated with aggressiveness features [66]. So, the identification of the phosphorylation sites involved in the spliceosome and the related proteins would be conducive to cancer pathogenesis and/or treatment.

RNA transport is an essential biological process via multiple complexes including nuclear pore complexes (NPCs), survival motor neuron complexes (SMNs), translation initiation factors (eIFs), and exon-junction complexes (EJCs). This study demonstrated that the phosphoproteins with statistically significant differences were involved in these complexes, and it strongly suggests that the mechanism of NF-PitNETs has a high correlation with the RNA transport phosphorylation. Previous research attested that the intriguing selectivity of NPCs is derived from intrinsically disordered proteins that are rich in phenylalanine-glycine repeats (FG-repeats). The phosphorylation sites of the FG-Nups are regulated by kinases and phosphatases. By means of conducting a one-bead-per-amino-acid (1BPA) model, the consequences indicate that phosphorylation leads to an impaired attraction between the residues, and thus results in the extension of FG-Nups and the formation of a weakened FG-network inside the NPC. The model also indicated that the phosphorylation would lead to an increase in the transport rate of inert molecules, and a decrease in nuclear transport receptors [67]. Therefore, the phosphorylation and dephosphorylation of the key cassette and the important pathway are worthy of further research, and might contribute to the treatment targets for NF-PitNETs.

Cells are covered by a surface layer of glycans, which is referred as the ‘glycocalyx’, and it plays a crucial role in vascular diseases such as atherosclerosis, stroke, hypertension, kidney disease, sepsis, and cancer [68]. For the glycocalyx, its primary glycosaminoglycans, including heparan sulphate (HS) and hyaluronic acid (HA), are usually elevated in cancer cells and are often involved in tumor growth and metastasis, while its core proteins such as syndecans and glypicans are degraded in vascular diseases. This results in the destruction of the vascular permeability barrier, which increases the approach of leucocytes to the endangium and causes the spread of inflammation, and then changes the mechanical transduction mechanism of endothelial cells to prevent disease [69]. In contrast, the glycocalyx on cancer cells is consistently exuberant, accelerating growth factor signaling and integrin clustering [69,70]. Moreover, the glycocalyx could promote the mechanotransduction of interstitial flow shear stress, which is increased in tumors to upregulate matrix metalloproteinase release, which improves cell motility and metastasis [69]. However, the significance of the glycocalyx in human is only beginning to be understood, along with the potential medicines that might be applied to increase and protect the glycocalyx in order to fight vascular disease [41], as well as a different set of agents that can decrease and destroy the cancer glycocalyx to suppress cell growth and metastasis [70]. Thus, abnormal phosphorylation in the glycocalyx of cancer could provide new evidence for the pathogenic mechanism of NF-PitNETs, and more detailed studies of the glycocalyx’s involvement in PitNET diseases and cancer will give rise to novel treatment modalities.

Vesicle trafficking within eukaryotic cells is a complex and precisely regulated multi-step process, including vesicle formation, vesicle translocation and vesicle fusion with the specific target membrane [24,43]. Fusion is the last step of vesicle transport, which is believed to be regulated by a family of proteins referred as SNAREs. The specific pairing of vesicles (v-SNAREs) with the complementary vesicle’s target membrane (t-SNARES) forms the “SNARE complex”, which promotes the fusion of the vesicle membrane with its specific target membrane [34]. According to previous studies, over 38 members of the SNARE family have been identified, which are distributed in distinct subcellular compartments in mammalian cells to regulate diverse transport activities. Mutation of SNAREs or dysfunction of SNARE complex formation may lead to cellular or physiological defects in humans [71]. Studies have found that CKII and CaMKII have been discovered to phosphorylate VAMP [72], by analogy with the other SNARE proteins, this newly identified phosphorylation of VAMP4 from DPPs of NF-PitNETs might affect synaptic vesicle docking and fusion, and contribute to the treatment targets for NF-PitNETs.

Tight junctions produce the paracellular epithelial barrier of ions and solutes, thus not only separating tissue spaces but also affecting the directional transcellular absorption and secretion [49,73]. According to previous studies, the pituicyte-derived factors regulate the decision of endothelial cells to adopt a permeable endothelial fate, which is considered related to the inhibition of Cyp26b activity, leading to the up-regulation of the tight junction protein claudin-5 [74]. Many proteins have been localized to tight junctions, as well as the key barrier components including transmembrane proteins which physically form the sealing contacts, such as members of the claudin (cldn), tight junction-associated proteins 1 (TJAP1) and junctional adhesion molecule (JAM or occludin) family of proteins [75]. The transmembrane proteins combined with scaffolding proteins, such as ZO-1, -2 and -3, among others, interact with cytoskeletal factors to mediate junctional integrity [76]. Many theories are available on the mechanism of binding interactions between these protein components, and one of the crucial mechanisms is phosphorylation. For example, a recent study demonstrated that over-expression of ocln S471 hampered the monolayer maturation and protein localization of normal tight junctions, in accordance with the discovery that phosphorylation at S471 regulates the normal interactions with ZO-1 and cell packing [77]. These significantly expressed phosphoproteins and phosphosites of NF-PitNETs involved in the molecules relating to tight junctions provide new evidence for the tight junctions regarding the pathogenesis of NF-PitNETs.

### 4.2. The Functions of Kinases and Their Corresponding Substrates Associated with Quantified Phosphoproteins

Kinases are enzymes that transfer a phosphate group to a protein; in contrast, phosphatases remove a phosphate group from a protein [18]. Together, in response to internal/external stimuli, these two enzymatic processes regulate multitudinous activities of proteins in a cell in virtually every imaginable way [19,78]. About 538 known kinases are encoded in the human genome, and these counter mechanisms vastly improve the plasticity of the epigenome [18]. Recent developments in the understanding of the fundamental molecular mechanisms regarding cancer cell signaling have elucidated a vital role for kinases in the metastases and carcinogenesis of diverse types of cancer [9,54,55,63]. Since many protein kinases are associated with promoting cell proliferation, migration, and survival, when active or over-expressed, they are often involved in oncogenesis [57]. Over the last four decades, genome-wide studies of kinase mutations have proved that genetic variants of specific kinases are related to cancer initiation, promotion, progression and recurrence [18,19]. Moreover, owning to the chromosomal reshuffling and genetic mutations, various human malignancies have been proven to be associated with the dysfunction of kinases and deactivated phosphatases [53,78]. Apart from the oncological matter, the dysregulation of kinases has been identified in numerous human disorders, such as neurological, immune, and infectious diseases [55,79]. Therefore, there is probably no better clinical media than kinases as the key targets for developing drugs in cancer therapy. Furthermore, apart from the enormous number of kinase-based drug targets, specific kinase inhibitors are less cytotoxic to non-cancerous cells, presenting a tumor-selective killing strategy with considerably lower toxic manifestations [78]. Currently, about one third of protein targets under research in the pharmaceutical industry are kinase-based therapeutic drugs [78]. The majority of the food and drug administration (FDA)-approved kinase inhibitors specifically target the ATP binding sites of kinase enzymes and exhibit therapeutic indications against tumorigenesis [78,79]. This characteristic of therapeutics shows a transformation from conventional chemotherapy to novel targeted cancer treatment [80]. Kinase inhibitors have overcome the main drawback of traditional cancer treatment, as they effectively distinguish between normal non-malignant cells and rapidly amplified cancer cells. Furthermore, kinase inhibitors are also applied in combination with cytotoxic chemotherapy or radiation therapy [81]. On account of this effective treatment, fewer off-target effects and low toxicities appeared in the cancer patient population [78,79]. Overall, kinases represent a new and promising approach to cancer therapy, and already provide beneficial clinical effects. In this study, seven kinases among the quantified phosphoproteins were identified, including GRP78, WSTF, PKN2, PRP4, LOK, NEK1, and AMPKA1 (Table 2; Figure 4). Of these, the substrates of kinases PKN2 and AMPKA1 were DPPs (Supplementary Table S2; Table 2), and the substrates of these kinases could provide new ideas for seeking drug-target and an effective therapeutic method for NF-PitNETs. For example, PKN2 is a PKC-related serine/threonine-protein kinase and it is related to tumor cell migration, invasion, and apoptosis [54]. For AMPKA1, the protein is the subunit of the 5′-prime-AMP-activated protein kinase (AMPK) which belongs to the ser/thr protein kinase family [79]. AMPK is a cellular energy sensor in eukaryotic cells whose activity, activated by the stimuli, could increase the cellular AMP/ATP ratio [55,79]. AMPK mediates the activities of numerous key metabolic enzymes by means of phosphorylation. It protects cells from stresses which induce ATP depletion by means of switching off ATP-consuming biosynthetic pathways [55,56]. Williams–Beuren syndrome transcription factor (WSTF) could regulate the constitutive phosphorylation of H2AX via the tyrosine kinase activity [82]. It is well-known that DNA damage defense mechanisms protect the genome from multiple hazardous substances. Previous studies have shown that phosphorylation of histone H2AX at Ser139 (γH2AX) is a noted PTM in terms of adjusting the DNA damage signaling pathway [83]. Further research on H2AX might be a new target in searching for drug treatments of tumors, including NF-PitNETs. In general, the regulation of kinase organization is critical for maintaining the normal physiological function of cells and investigating the occurrence and progress of neoplasms. Approximately 150 kinase-specific drugs are in clinical trials, while more kinase-targeting inhibitors are in the preclinical stage [78]. In addition, specific cancer genetics, the complex tumor microenvironment, complicated pharmacogenomics, and possible drug resistance would determine how useful a kinase-targeting drug will be in the clinical treatment of a given disease [55,81]. Therefore, this is a promising area where these kinases and their substrates identified in this study might represent new drug targets for NF-PitNETs and provide new ways of thinking to investigate pathogenesis and therapeutic approaches in NF-PitNETs.

### 4.3. The Phosphorylation of Calnexin in NF-PitNETs

Calnexin serves as an endoplasmic reticulum (ER)-specific type of transmembrane protein, and plays an important role in the well-aligned synthesis of glycoproteins [84]. Calnexin discharges into the extracellular region by means of assisting with glycoproteins and the modification (phosphorylation at two serine residues (Ser554/564) via protein kinase CK2 [84]. A previous study found that calnexin improved the expression of PD-1 in T-cells by suppressing the DNA methylation state in a PD-1 promoter, and revealed the mechanism by which calnexin in tumor cells regulates the anti-tumor response of T-cells, suggesting that calnexin may be a potential target for improving anti-tumor immunotherapy [85]. This study found that the phosphopeptide quantity of calnexin in NF-PitNETs samples was about ten times greater than that in the control group. Immunoprecipition (IP) analysis also proved that the phosphorylation of calnexin in NF-PitNETs is higher than that in controls. It was hypothesized that an increased level of calnexin phosphorylation in NF-PitNETs might result in the abnormalities in ER processing, and this study also provides insight into the search for effective drugs and therapeutic targets for NF-PitNETs.

## 5. Strengths and Limitations

This study provided the first DPP profiling (595 DPPs with 1412 phosphosites) in NF-PitNETs and the corresponding phosphorylation-mediated functional characteristics changes and signaling pathway network changes. The key kinase profiling, including GRP78, WSTF, PKN2, PRP4, LOK, NEK1, and AMPKA1, was identified in NF-PitNET pathogenesis with the kinase analysis of those DPPs; and the substrate of these kinases could provide new ideas to find drug targets for NF-PitNETs. These findings are a precious resource in understanding the biological roles of protein phosphorylation in NF-PitNET pathogenesis and in discovering effective phosphoprotein biomarkers and therapeutic targets and drugs for the management of NF-PitNETs.

This set of DPP data is accurate and reliable in NF-PitNETs relative to control pituitary tissues, which is evidenced in the follow two aspects. First, the randomly selected DPP—calnexin—was further verified with IP and Western blot in the NF-PitNETs and control pituitary tissues, the result is consistent with the results of quantitative phosphoproteomics. Second, NF-PitNET samples for proteomics did not show immunoreactivity for GH or ACTH, whereas the control pituitary tissue was the whole pituitary gland, including at least five types of pituitary cells (somatotroph secreting GH, corticotroph secreting ACTH, prolactroph secreting PRL, tyrotroph secreting TSH, and gonadotroph secreting FSH/LH). It is very interesting to note that our quantitative phosphoproteomics analysis (Supplementary Table S2) found that phosphorylated GH1 was significantly decreased (ratio of T/N = 0.08, *p* = 1.93 × 10^−6^, at pSer176), and phosphorylated POMC (POMC is the precursor of ACTH) was also significantly decreased (ratio of T/N = 0.24, *p* = 3.01 × 10^−6^, at pSer108; ratio of T/N = 0.03, *p* = 8.83 × 10^−6^, at pThr58; and ratio of T/N = 0.49, *p* = 8.78 × 10^−4^, at pSer168), in NF-PitNET vs. control pituitary tissue groups. These are very reasonable results, which in turn confirm the accuracy and reliability of our quantitative phosphoproteomics analysis between NF-PitNET and control pituitary tissues. Theoretically, NF-PitNETs were neoplasms with a monoclonal cell origin, and NF-PitNET tissues should consist of a single cell type. However, this phosphoproteomics analysis also detected GH and POMC in NF-PitNET groups; this might be due to contamination of normal pituitary tissue during the tumor removal procedure.

However, one must realize that some limitations existed for this type of quantitative phosphoproteomic data. First, this set of DPP data was derived from a limited sample size (four NF-PitNETs vs. four controls). To convert these findings into routine practice, it is necessary to significantly expand the sample size for further validating and studying molecular mechanisms in detail, and the functional roles of those DPPs, and also identifying the difference among NF-PitNETs as well as the difference between NF-PitNETs and control pituitary tissues. Second, for quantitative phosphoproteomics analysis, the NF-PitNET group was the mixed sample, including two FSH(+)- and two negative hormone-NF-PitNETs (Supplementary Table S1). According to the new 2017 WHO classification of PitNET [86,87], two FSH(+)-NF-PitNETs were gonadotroph adenomas. Two NF-PitNETs with negative immunostaining of pituitary hormones could be gonadotroph adenomas, silent corticotroph adenomas, silent adenomas of Pit-1 derivation, or null cell adenomas. Although our phosphoproteomics studies can reflect the difference between NF-PiNETs and control pituitary tissues, in future studies, it is necessary to separate different subtypes of NF-PitNETs relative to control pituitary tissues for more accurate discrimination. Third, for our IP and WB experiments of phosphorylated calnexin, NF-PitNET group included two NF-PitNET samples with negative immunostaining of pituitary hormones, and one NF-PitNET sample with positive immunostaining for ACTH and FSH (Supplementary Table S1). Although it can distinguish the difference between NF-PitNETs and control pituitary tissues, NF-PitNETs with positive immunostaining for ACTH and FSH were rare [88,89], and could be gonadotroph adenoma, silent corticotroph adenoma, or plurihormonal adenoma [90]; thus, in future studies with the expanded sample size, it is necessary to separate the different subtypes of NF-PitNET samples for more accurate discrimination of the differences in calnexin. Moreover, we should state that PitNET tissue samples are very precious, and the previously limited availability of PitNET samples caused insufficient gonadotroph adenoma samples for this phosphoproteomics experiment. Fourth, differences in collection method and/or the storage of samples could be the source of bias that affect the findings regarding differences between NF-PitNETs and control pituitary tissues, because the NF-PitNET tissue samples were obtained from neurosurgery in living patients, whereas the five control pituitary tissue samples were taken from post mortem tissues. However, this difference is unavoidable for any human PitNET tissue research. Fifth, the different ethnic origin of the samples is also a potential bias. For phosphoproteomics analysis, the NF-PitNET tissues were from four Chinese patients, while the control pituitary tissues were from four post mortem tissues (three subjects from the USA of Caucasian ethnic origin and one subject of African-American ethnic origin) (Supplementary Table S1). In future studies, it will be necessary to investigate the detailed differences in the phosphorylation level of each phosphoprotein in NF-PitNETs and control pituitary tissues among samples of different ethnic origin (Chinese, African-American, and American Caucasian tissue samples).

Here, it is worth mentioning one of our previous studies that identified differentially expressed proteins (DEPs) between samples of control pituitary tissues from patients of different genders (female vs. male), different ages (30-, 40-, and 50-year-old groups), and different ethnic origin (black vs. white) in the analysis of the heterogeneity of human pituitary proteomes [91]. Our study found that the heterogeneity of human control pituitary proteomes did not significantly affect the differences (DEPs) between NF-PITNETs and control pituitary tissues [92,93]. It clearly demonstrated that the PitNET disease-induced differences might be much larger than, or different from, the gender-, age-, and ethnic origin-induced differences, or the potential differences derived from tissue origins (post mortem vs. biopsies). These findings might help to assure the reliability of our phosphoproteomics results.

Moreover, for biological omics analysis, one should realize the difference between statistical significance and biological significance [94]. The statistically significant results must be rationalized with their biological significance; otherwise, the statistically significant results cannot be used as biologically significant results. For example, this present study found that vasopressin-regulated water reabsorption (Supplementary Figure S1.4) and salivary secretion (Supplementary Figure S1.6) were two statistically significant pathways with KEGG pathway analysis. It is well-known that NF-PitNETs arise from the anterior pituitary gland, the normal production, storage, and release of vasopressin is in the posterior pituitary gland [95,96]. For this phosphoproteomics analysis, the control tissues comprised the whole pituitary gland, including the anterior and posterior pituitary glands. Therefore, the detected vasopressin-regulated water reabsorption pathway changes may not be derived from PitNET disease, but may highly possibly be derived from experimental bias. Similarly, the microscopic salivary gland [97] located in the human pituitary is not well- known, and we have not found any relevance between a salivary gland in the posterior pituitary and NF-PitNETs from the anterior pituitary gland. Therefore, the detected statistically significant salivary secretion pathway changes cannot be recognized as a biologically significant result for NF-PitNETs.

## 6. Conclusions

TMT-based quantitative proteomics coupled with TiO_2_ enrichment of phosphopeptides effectively identified and quantified protein phosphorylation at residues of Ser (S), Tyr (Y), and Thr (T) in human NF-PitNETs compared to controls. This study provided the first quantitative phosphoproteomic profiling of human NF-PitNETs relative to control pituitaries, and phosphorylation-mediated biological processes and molecular pathway network changes in NF-PitNETs. These findings add new information on the roles of phosphorylation in PitNETs and provide new insight into elucidating the molecular mechanisms of NF-PitNETs, exploring significant therapeutic targets, and discovering new biomarkers for the effective management of NF-PitNETs.

## Figures and Tables

**Table 1:** The clustered functional characteristics of differentially phosphorylated proteins in human nonfunctional PitNETs.

Category	ID		Count	%	*p*-Value	Genes
Annotation Cluster 1 Enrichment Score: 12.9						
GOTERM_CC_DIRECT	GO:0005913	cell–cell adherens junction	42	7.4	5.37 × 10^−15^	Q9UHB6, Q9UPN3, P18206, Q9H0B6, A0A087WUZ3, A0A0U4BW16, Q9UDY2, P35221, A0A024R1S8, Q9C0C2, Q15762, P55196, Q6PKG0, Q09666, O60716, C9J6P4, P42166, A0A024R4E5, P07948, E9PRY8, Q9H4G0, Q9ULH1, P35611, Q9H2G2, P21333, Q07960, O00567, Q92522, P35579, Q13813, Q15149, O60763, P08238, O95292, Q9UQN3, Q9BY44, A0A024RAN2, Q16513, O76021, E7EX44, Q92597, P26232, Q14247
GOTERM_MF_DIRECT	GO:0098641	cadherin binding involved in cell–cell adhesion	40	7.0	2.04 × 10^−14^	Q9UHB6, Q9UPN3, P18206, Q9H0B6, A0A087WUZ3, Q9UDY2, P35221, A0A0U4BW16, A0A024R1S8, Q9C0C2, P55196, Q6PKG0, Q09666, O60716, C9J6P4, P42166, A0A024R4E5, E9PRY8, Q9H4G0, Q9ULH1, P35611, Q9H2G2, P21333, Q07960, O00567, Q92522, P35579, Q13813, Q15149, O60763, P08238, O95292, Q9UQN3, Q9BY44, A0A024RAN2, Q16513, O76021, E7EX44, Q92597, P26232, Q14247
GOTERM_BP_DIRECT	GO:0098609	cell–cell adhesion	34	6.0	2.25 × 10^−11^	Q9UHB6, Q9UPN3, Q9H0B6, A0A087WUZ3, Q9UDY2, A0A024R1S8, Q9C0C2, P55196, Q6PKG0, Q09666, C9J6P4, P42166, A0A024R4E5, E9PRY8, Q9H4G0, Q9ULH1, P35611, Q9H2G2, Q07960, O00567, Q92522, Q13813, Q15149, O60763, P08238, O95292, Q9BY44, Q9UQN3, A0A024RAN2, Q16513, O76021, E7EX44, Q92597, Q14247
Annotation Cluster 2 Enrichment Score: 7.9						
GOTERM_BP_DIRECT	GO:0006405	RNA export from nucleus	15	2.6	9.39 × 10^−10^	Q15287, Q13247, O95391, Q96FV9, P52948, A0A0S2Z4Z6, J3KTL2, Q08170, Q16629, O75694, Q05519, Q13242, P35658, P09651, Q01130
GOTERM_BP_DIRECT	GO:0006406	mRNA export from nucleus	19	3.3	1.69 × 10^−9^	Q15287, Q9BRD0, Q13247, O75494, O95391, L0R530, Q96FV9, P52948, A0A0S2Z4Z6, J3KTL2, Q08170, P49792, Q16629, O75694, Q05519, Q13242, Q9P2I0, P35658, Q01130
GOTERM_BP_DIRECT	GO:0031124	mRNA 3’-end processing	13	2.3	3.10 × 10^−8^	Q15287, Q08170, Q13247, Q16629, Q12996, O95391, Q05519, Q13242, Q96FV9, Q9P2I0, A0A0S2Z4Z6, Q01130, J3KTL2
GOTERM_BP_DIRECT	GO:0006369	termination of RNA polymerase II transcription	13	2.3	5.63 × 10^−7^	Q15287, Q08170, Q13247, Q16629, Q12996, O95391, Q05519, Q13242, Q96FV9, Q9P2I0, A0A0S2Z4Z6, Q01130, J3KTL2
Annotation Cluster 3 Enrichment Score: 4.0						
GOTERM_CC_DIRECT	GO:0014731	spectrin-associated cytoskeleton	6	1.1	1.24 × 10^−6^	Q12955, Q08495, P16157, A0A087WUZ3, P11171, P11277
GOTERM_CC_DIRECT	GO:0008091	spectrin	5	0.9	8.97 × 10^−5^	A0A087WUZ3, P11171, P11277, O43491, Q13813
GOTERM_BP_DIRECT	GO:0051693	actin filament capping	4	0.7	6.84 × 10^−3^	Q08495, A0A087WUZ3, P11277, Q13813
Annotation Cluster 4 Enrichment Score: 3.0						
GOTERM_BP_DIRECT	GO:0043044	ATP-dependent chromatin remodeling	7	1.2	5.76 × 10^−5^	Q13547, P07910, Q92769, Q14839, B4DY08, Q92922, O96019, F8VXC8
GOTERM_MF_DIRECT	GO:0031492	nucleosomal DNA binding	9	1.6	8.71 × 10^−5^	Q13547, P05114, P07910, Q92769, Q14839, B4DY08, Q92922, P49450, O96019, F8VXC8
GOTERM_CC_DIRECT	GO:0000790	nuclear chromatin	13	2.3	1.39 × 10^−2^	P51531, Q9H1E3, Q9Y618, P52701, Q92769, O75376, Q14839, O96019, F8VXC8, Q13547, P07910, P16402, B4DY08, Q92922
GOTERM_MF_DIRECT	GO:0000980	RNA polymerase II distal enhancer sequence-specific DNA binding	7	1.2	1.68 × 10^−2^	Q13547, P07910, Q92769, Q14839, B4DY08, Q92922, O96019, F8VXC8
Annotation Cluster 5 Enrichment Score: 2.9						
GOTERM_CC_DIRECT	GO:0071564	npBAF complex	5	0.9	3.28 × 10^−4^	P51531, Q8WUB8, Q92922, O96019, F8VXC8
GOTERM_CC_DIRECT	GO:0016514	SWI/SNF complex	5	0.9	8.42 × 10^−4^	P51531, Q92922, Q8NFD5, O96019, F8VXC8
GOTERM_CC_DIRECT	GO:0071565	nBAF complex	4	0.7	7.65 × 10^−3^	P51531, Q92922, Q8NFD5, F8VXC8
Annotation Cluster 6 Enrichment Score: 2.4						
GOTERM_BP_DIRECT	GO:0007064	mitotic sister chromatid cohesion	5	0.9	7.31 × 10^−4^	Q9NTI5, Q7Z5K2, Q29RF7, Q6KC79, Q9UQE7
GOTERM_CC_DIRECT	GO:0000775	chromosome, centromeric region	7	1.2	7.06 × 10^−3^	Q9NTI5, P83916, Q13185, Q7Z5K2, Q29RF7, P49450, Q9UQE7
GOTERM_BP_DIRECT	GO:0007062	sister chromatid cohesion	9	1.6	1.53 × 10^−2^	Q9NTI5, P49792, O75122, Q7Z5K2, Q29RF7, P49450, Q9UQE7, Q8WYP5, P52948
Annotation Cluster 7 Enrichment Score: 2.3						
GOTERM_BP_DIRECT	GO:0061025	membrane fusion	9	1.6	5.61 × 10^−5^	O00161, D3DUW5, P63027, Q05193, Q16623, O60763, Q9UNZ2, Q9UQ16, P61266
KEGG_PATHWAY	hsa04130:S	NARE interactions in vesicular transport	6	1.1	4.25 × 10^−3^	O00161, P63027, Q16623, O75396, P61266, O75379
GOTERM_MF_DIRECT	GO:0005484	SNAP receptor activity	6	1.1	7.42 × 10^−3^	O00161, P63027, Q16623, O75396, P61266, O75379
GOTERM_BP_DIRECT	GO:0016192	vesicle-mediated transport	12	2.1	8.19 × 10^−3^	P63027, Q16623, O75396, Q13439, O00203, O75131, P61266, P35606, O75379, Q13367, Q9UPT6, Q9UN37
GOTERM_CC_DIRECT	GO:0031201	SNARE complex	6	1.1	2.10 × 10^−2^	O00161, P63027, Q16623, O75396, P61266, O75379
GOTERM_BP_DIRECT	GO:0017157	regulation of exocytosis	4	0.7	4.62 × 10^−2^	P63027, Q16623, P61266, Q9Y6V0
Annotation Cluster 8 Enrichment Score: 2.3						
GOTERM_BP_DIRECT	GO:0016925	protein sumoylation	14	2.5	7.34 × 10^−5^	Q02880, Q12888, Q99502, A0A024R2M8, Q14676, L0R530, Q8NDX5, P52948, P07910, P49792, O75694, B4DY08, Q9UQE7, P35658, P29590
GOTERM_BP_DIRECT	GO:1900034	regulation of cellular response to heat	9	1.6	2.30 × 10^−3^	Q96B36, P07900, P08238, P49792, O75694, B3KUY2, L0R530, P35658, P52948
GOTERM_BP_DIRECT	GO:0007077	mitotic nuclear envelope disassembly	7	1.2	2.31 × 10^−3^	P02545, P49792, O75694, P17252, L0R530, P35658, P52948
GOTERM_BP_DIRECT	GO:0006409	tRNA export from nucleus	5	0.9	1.69 × 10^−2^	P49792, O75694, L0R530, P35658, P52948
GOTERM_BP_DIRECT	GO:0010827	regulation of glucose transport	5	0.9	1.87 × 10^−2^	P49792, O75694, L0R530, P35658, P52948
GOTERM_BP_DIRECT	GO:0075733	intracellular transport of virus	6	1.1	2.10 × 10^−2^	P49792, O75694, L0R530, P35658, O00505, P52948
GOTERM_CC_DIRECT	GO:0044615	nuclear pore nuclear basket	3	0.5	4.86 × 10^−2^	P49792, P35658, P52948
Annotation Cluster 9 Enrichment Score: 2.2						
GOTERM_BP_DIRECT	GO:0031032	actomyosin structure organization	7	1.2	1.52 × 10^−4^	P35580, Q9H4G0, Q9Y2J2, Q92614, A0A0U4BW16, P11171, P35579, O43491
GOTERM_CC_DIRECT	GO:0019898	extrinsic component of membrane	7	1.2	3.67 × 10^−2^	Q9UEW8, Q9H4G0, Q9Y2J2, Q96C24, P11171, Q9Y4F1, O43491
GOTERM_BP_DIRECT	GO:0030866	cortical actin cytoskeleton organization	4	0.7	3.76 × 10^−2^	Q9H4G0, Q9Y2J2, P11171, O43491
Annotation Cluster 10 Enrichment Score: 2.1						
GOTERM_BP_DIRECT	GO:0033523	histone H2B ubiquitination	4	0.7	1.50 × 10^−3^	Q5VTR2, Q6PD62, Q8WVC0, Q8N7H5
GOTERM_BP_DIRECT	GO:0010390	histone monoubiquitination	4	0.7	4.13 × 10^−3^	Q5VTR2, Q6PD62, Q8WVC0, Q8N7H5
GOTERM_BP_DIRECT	GO:0001711	endodermal cell fate commitment	3	0.5	1.34 × 10^−2^	Q6PD62, Q8WVC0, Q8N7H5
GOTERM_CC_DIRECT	GO:0016593	Cdc73/Paf1 complex	3	0.5	1.71 × 10^−2^	Q6PD62, Q8WVC0, Q8N7H5
GOTERM_BP_DIRECT	GO:0045638	negative regulation of myeloid cell differentiation	4	0.7	2.02 × 10^−2^	Q96T37, Q6PD62, Q8WVC0, Q8N7H5
Annotation Cluster 11 Enrichment Score: 1.5						
GOTERM_BP_DIRECT	GO:0006446	regulation of translational initiation	5	0.9	2.51 × 10^−2^	B5ME19, O60841, E7EX17, Q59GJ0, P04792, P23588
GOTERM_BP_DIRECT	GO:0006413	translational initiation	10	1.8	2.79 × 10^−2^	Q8NE71, P05387, Q13144, B5ME19, Q6PKG0, Q9BY44, O60841, P05388, E7EX17, Q59GJ0, P23588
GOTERM_MF_DIRECT	GO:0003743	translation initiation factor activity	6	1.1	4.37 × 10^−2^	Q13144, B5ME19, Q9BY44, O60841, E7EX17, Q59GJ0, P23588
Annotation Cluster 12 Enrichment Score: 1.4						
GOTERM_BP_DIRECT	GO:1904903	ESCRT III complex disassembly	3	0.5	3.70 × 10^−2^	A0A024R2C5, Q9UQN3, Q9UN37
GOTERM_BP_DIRECT	GO:1902188	positive regulation of viral release from host cell	3	0.5	4.44 × 10^−2^	A0A024R2C5, Q9UQN3, Q9UN37
GOTERM_BP_DIRECT	GO:0006997	nucleus organization	4	0.7	4.62 × 10^−2^	A0A024R2C5, Q9UQN3, Q14980, Q9UN37

**Table 2:** The identified kinases based on the identified phosphorylated proteins in human nonfunctional PitNETs.

Accession	KINASE	GENE	SUB	Description	Coverage	Proteins	Unique Peptides
P11021	GRP78	HSPA5	GRP78	78 kDa glucose-regulated protein OS = Homo sapiens GN = HSPA5 PE = 1 SV = 2 [GRP78_HUMAN]	6.57	12	2
Q9UIG0	WSTF	BAZ1B	H2AX	Tyrosine-protein kinase BAZ1B OS = Homo sapiens GN = BAZ1B PE = 1 SV = 2 [BAZ1B_HUMAN]	1.15	1	1
Q16513	PKN2	PKN2	pyrin	Serine/threonine-protein kinase N2 OS = Homo sapiens GN = PKN2 PE = 1 SV = 1 [PKN2_HUMAN]	1.42	1	1
Q13523	PRP4	PRPF4B	ELK1	Serine/threonine-protein kinase PRP4 homolog OS = Homo sapiens GN = PRPF4B PE = 1 SV = 3 [PRP4B_HUMAN]	5.46	2	1
O94804	LOK	STK10	Radixin, Ezrin, PLK1, Moesin	Serine/threonine-protein kinase 10 OS = Homo sapiens GN = STK10 PE = 1 SV = 1 [STK10_HUMAN]	1.55	1	1
Q96PY6	NEK1	NEK1	TAZ, VDAC1, VHL, RAD54L	Serine/threonine-protein kinase Nek1 OS = Homo sapiens GN = NEK1 PE = 1 SV = 2 [NEK1_HUMAN]	1.27	1	1
Q13131	AMPKA1	PRKAA1		5’-AMP-activated protein kinase catalytic subunit alpha-1 OS = Homo sapiens GN = PRKAA1 PE = 1 SV = 4 [AAPK1_HUMAN]	1.79	1	1

## Data Availability

All data are available in this article and the Supplementary Materials.

## Supplementary Materials

The following are available online at https://www.mdpi.com/article/10.3390/cells10092225/s1, Supplementary Figure S1: The significant signaling pathways involved in phosphoproteins in human nonfunctional PitNETs. Supplementary Table S1: Clinical information of nonfunctional PitNETs and control tissue samples. Supplementary Table S2: Differentially phosphorylated proteins identified in nonfunctional PitNETs and control pituitaries. Supplementary Table S3: Statistically significant GO biological processes (BP) of differentially phosphorylated proteins in human nonfunctional PitNETs. Supplementary Table S4: Statistically significant GO cellular components (CC) of differentially phosphorylated proteins in human nonfunctional PitNETs. Supplementary Table S5: Statistically significant GO molecular functions (MF) of differentially phosphorylated proteins in human nonfunctional PitNETs.
