# Supplementary material for: Phosphorylation-Mediated Molecular Pathway Changes in Human Pituitary Neuroendocrine Tumors Identified by Quantitative Phosphoproteomics
Source: Cells. 2021 Aug 27;10(9):2225. doi: 10.3390/cells10092225 (PMC8471408; doi:10.3390/cells10092225)
Supplement: Supplementary file 1 [file cells-10-02225-s001.zip › Figure S1_v6.pptx]

## Slide 1
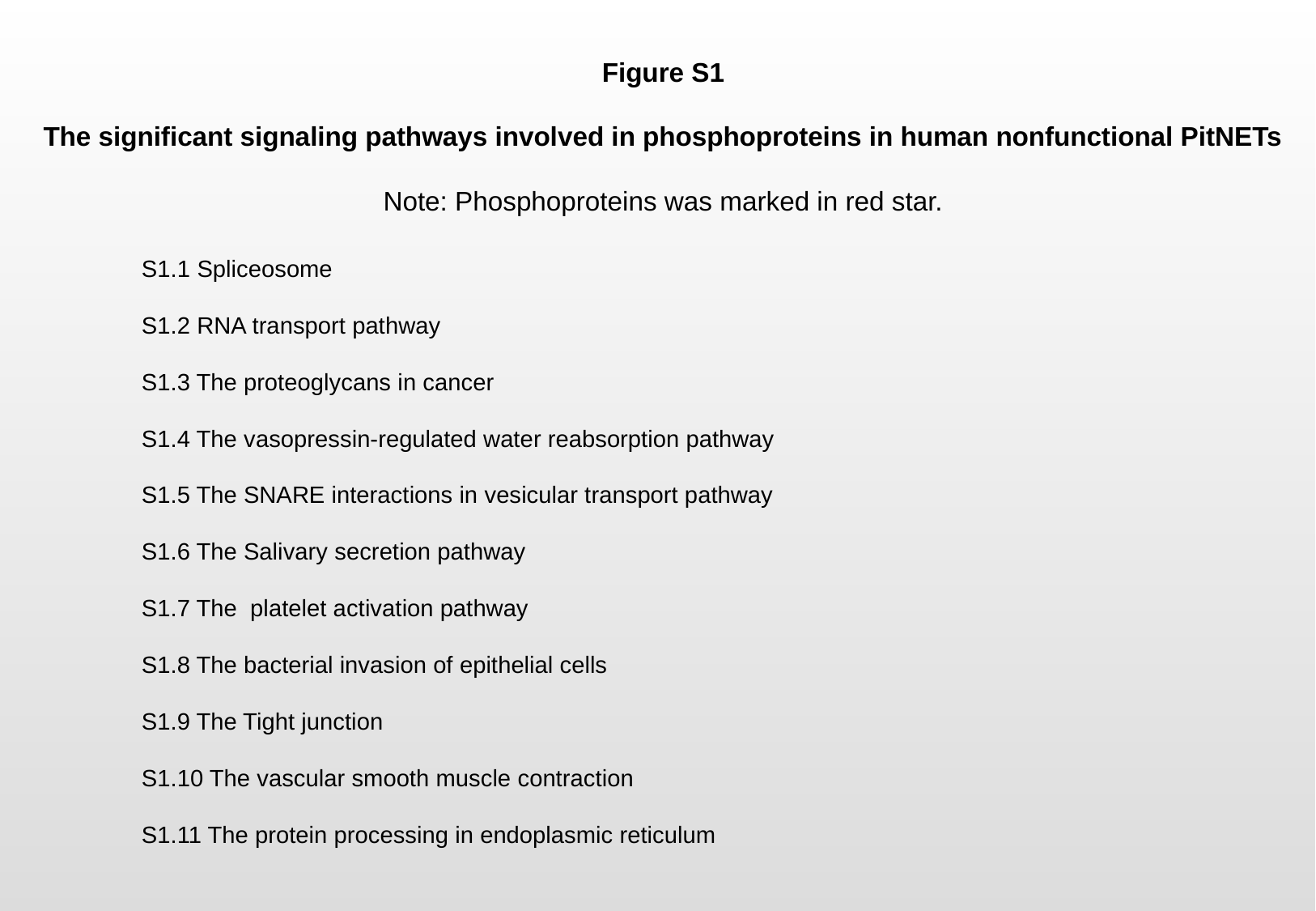

Figure S1
The significant signaling pathways involved in phosphoproteins in human nonfunctional PitNETs
Note: Phosphoproteins was marked in red star.
S1.1 Spliceosome
S1.2 RNA transport pathway
S1.3 The proteoglycans in cancer
S1.4 The vasopressin-regulated water reabsorption pathway
S1.5 The SNARE interactions in vesicular transport pathway
S1.6 The Salivary secretion pathway
S1.7 The platelet activation pathway
S1.8 The bacterial invasion of epithelial cells
S1.9 The Tight junction
S1.10 The vascular smooth muscle contraction
S1.11 The protein processing in endoplasmic reticulum

## Slide 2
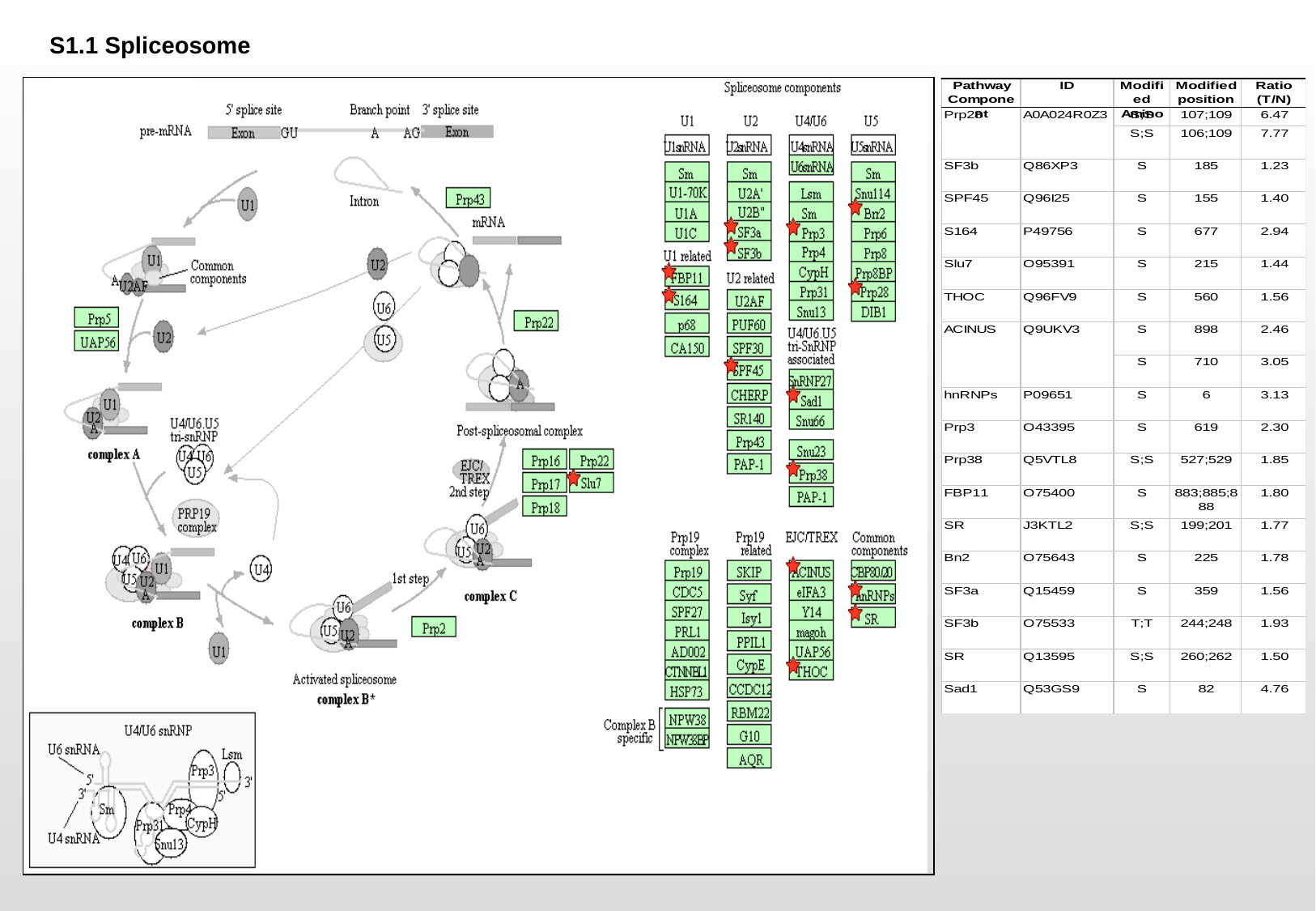

S1.1 Spliceosome

## Slide 3
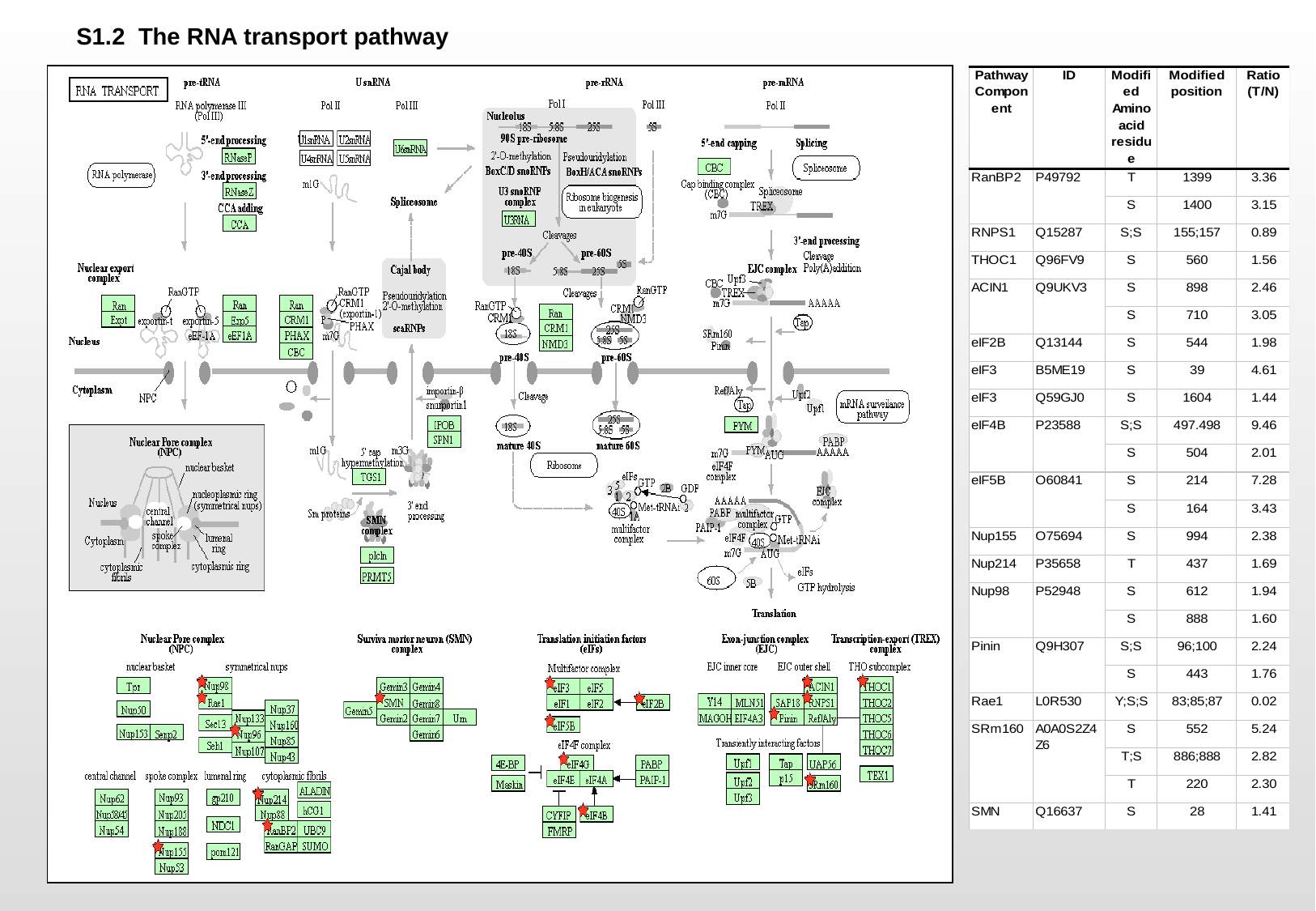

S1.2 The RNA transport pathway

## Slide 4
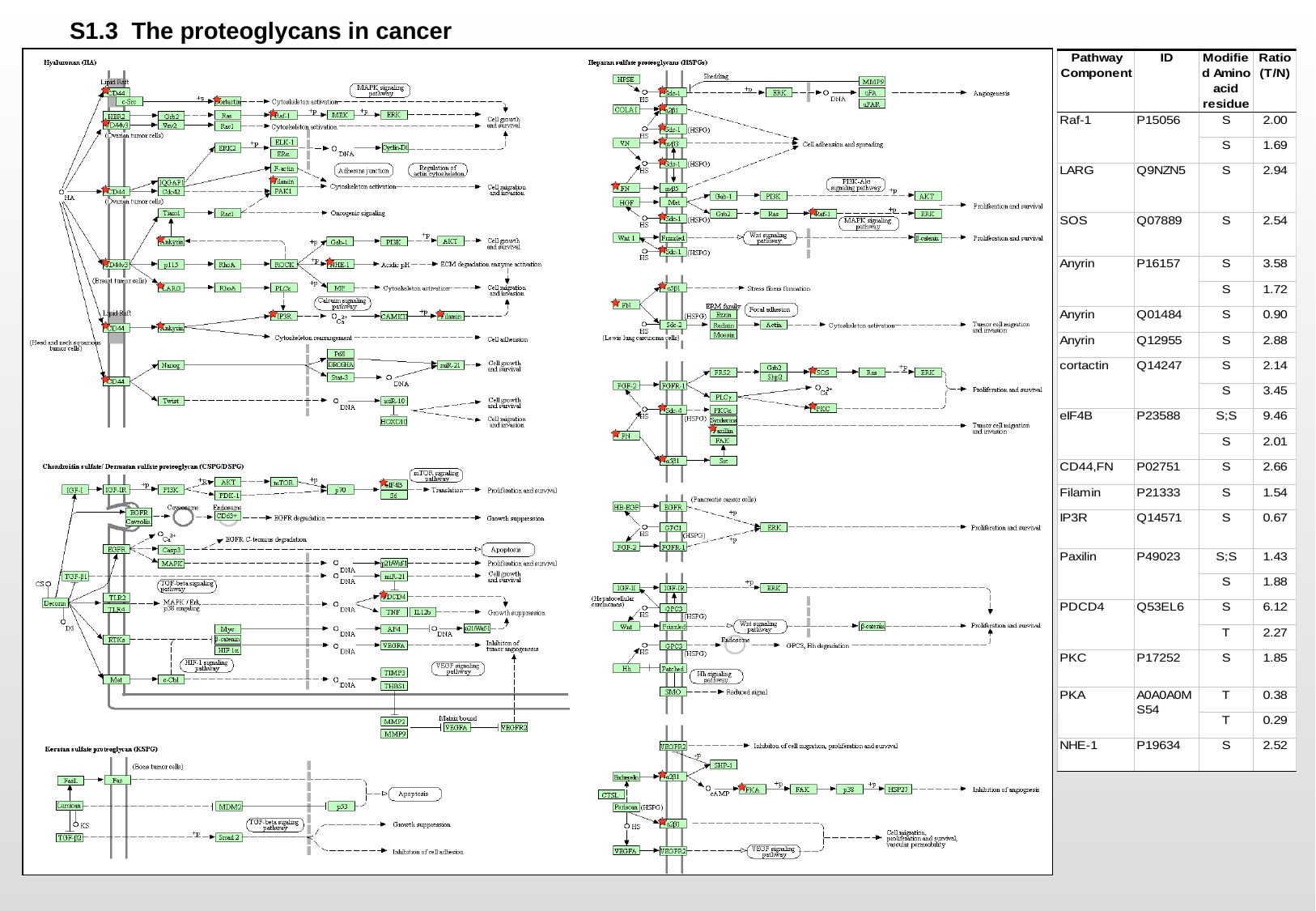

S1.3 The proteoglycans in cancer

## Slide 5
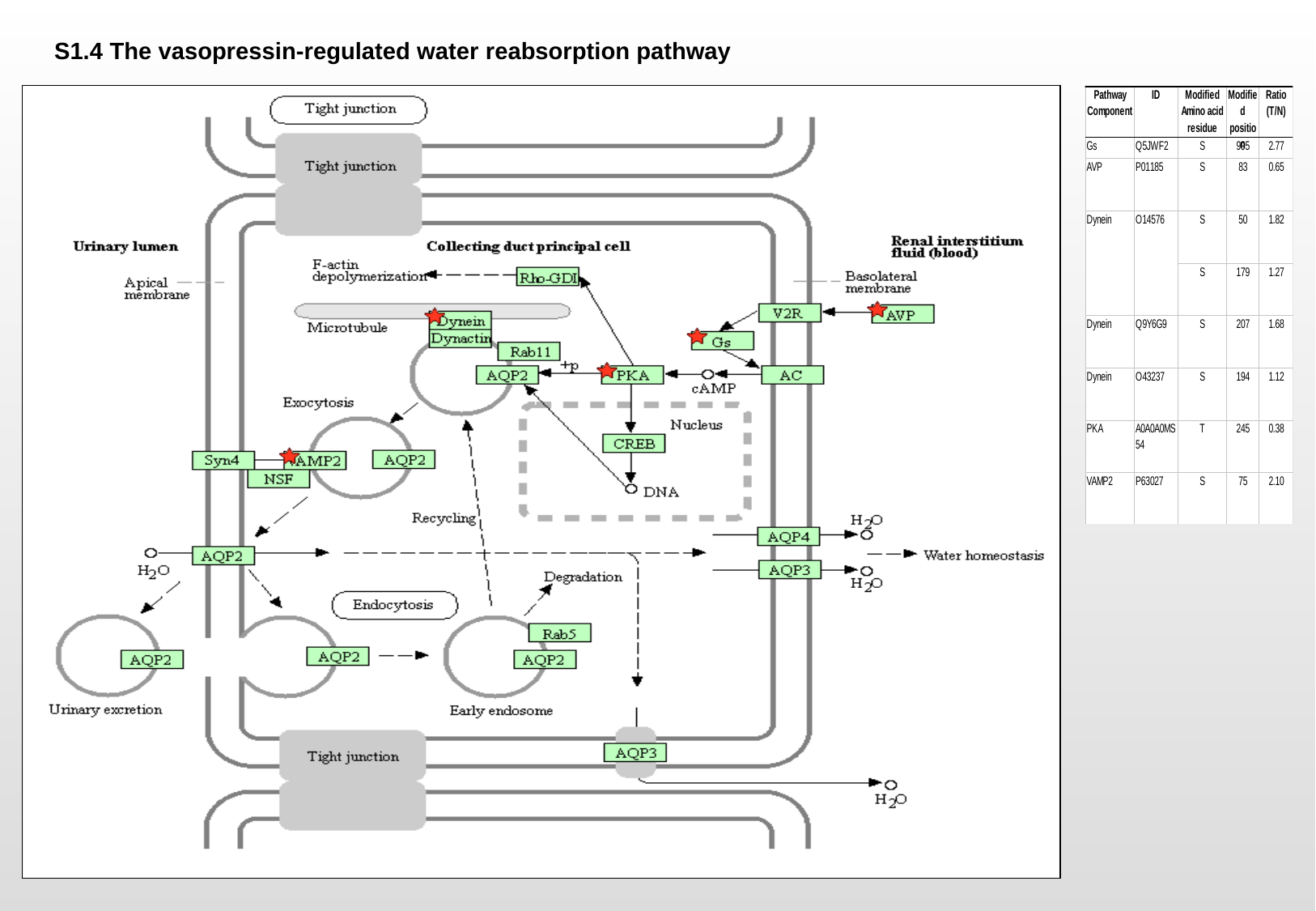

S1.4 The vasopressin-regulated water reabsorption pathway

## Slide 6
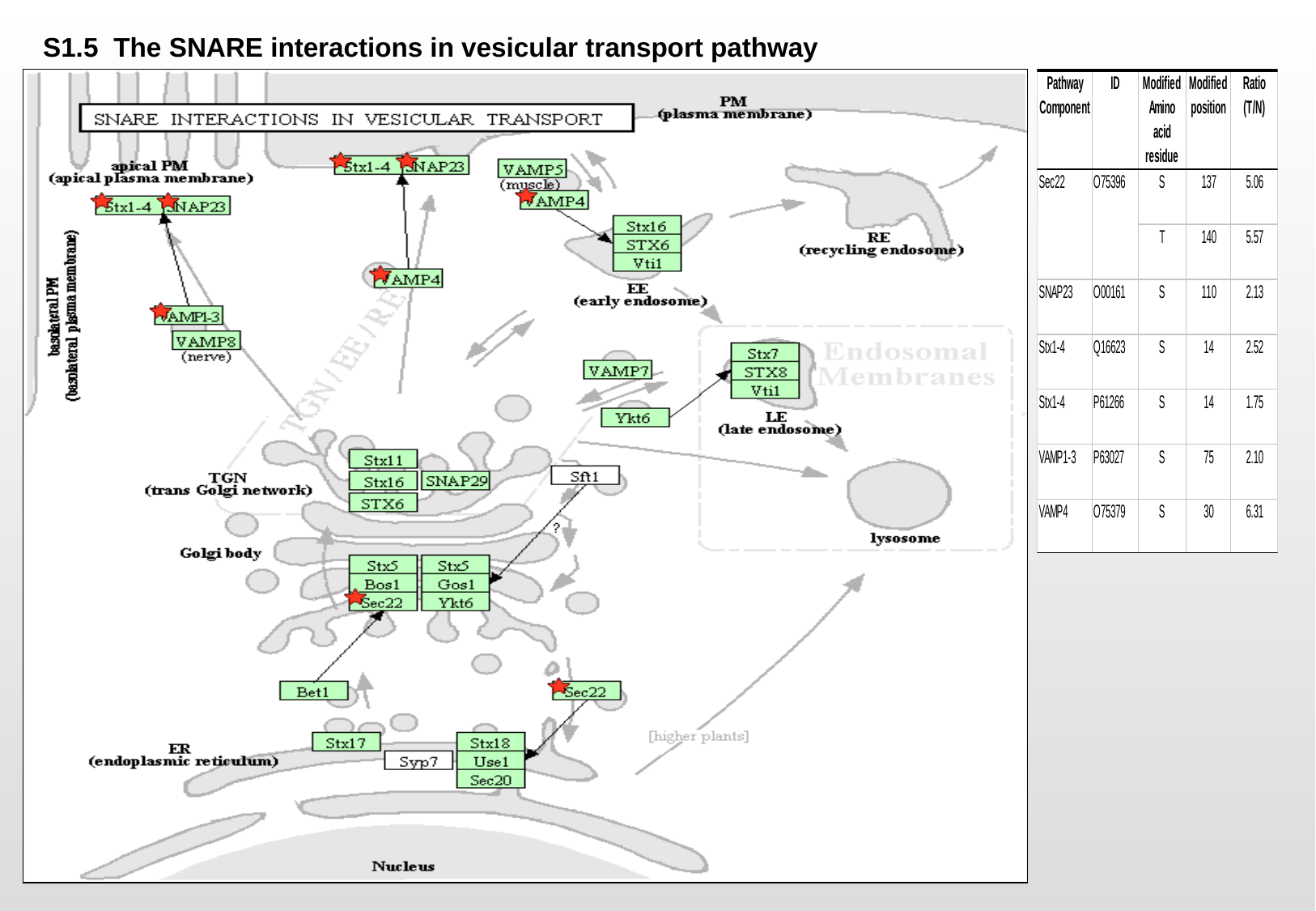

S1.5 The SNARE interactions in vesicular transport pathway

## Slide 7
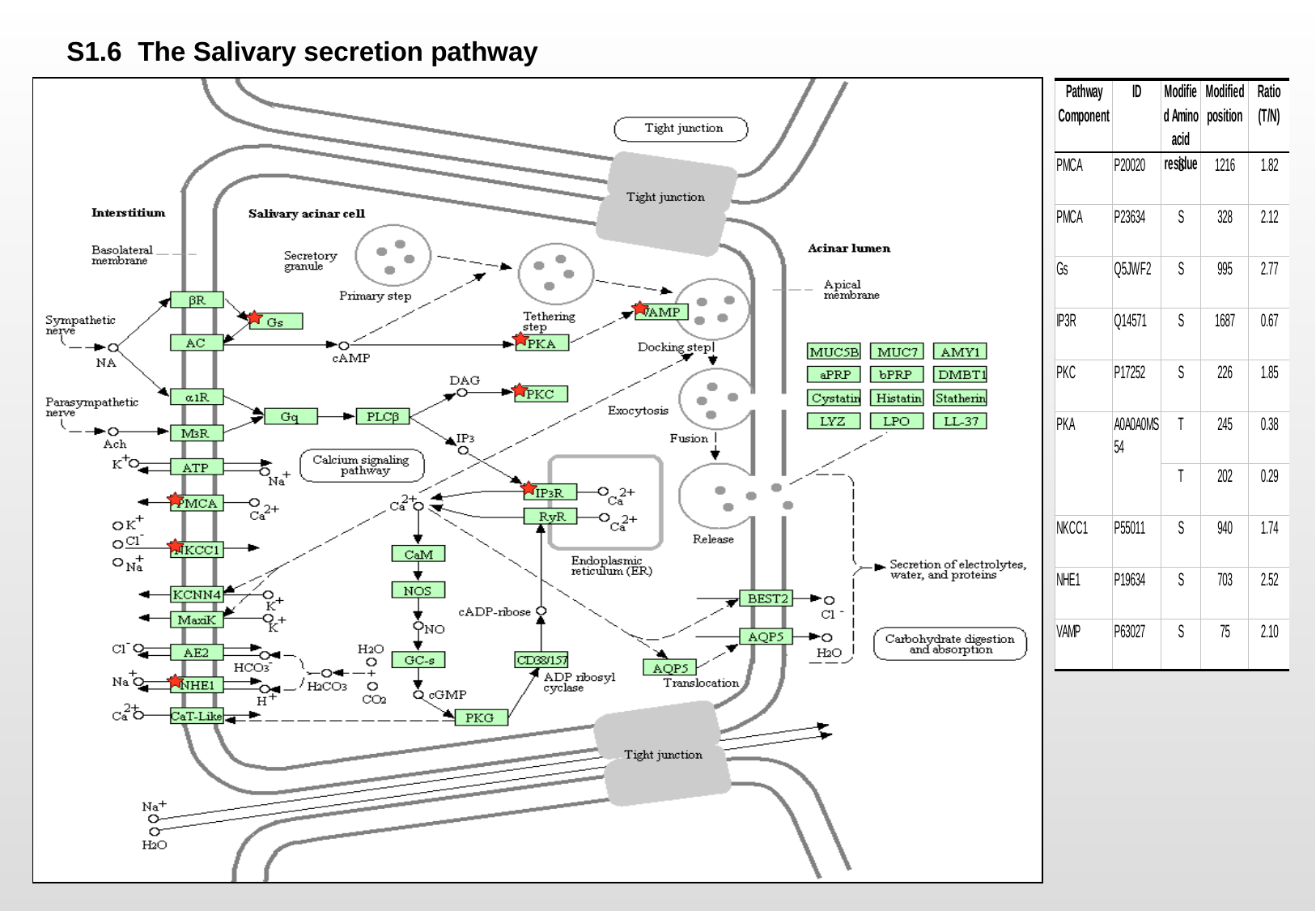

S1.6 The Salivary secretion pathway

## Slide 8
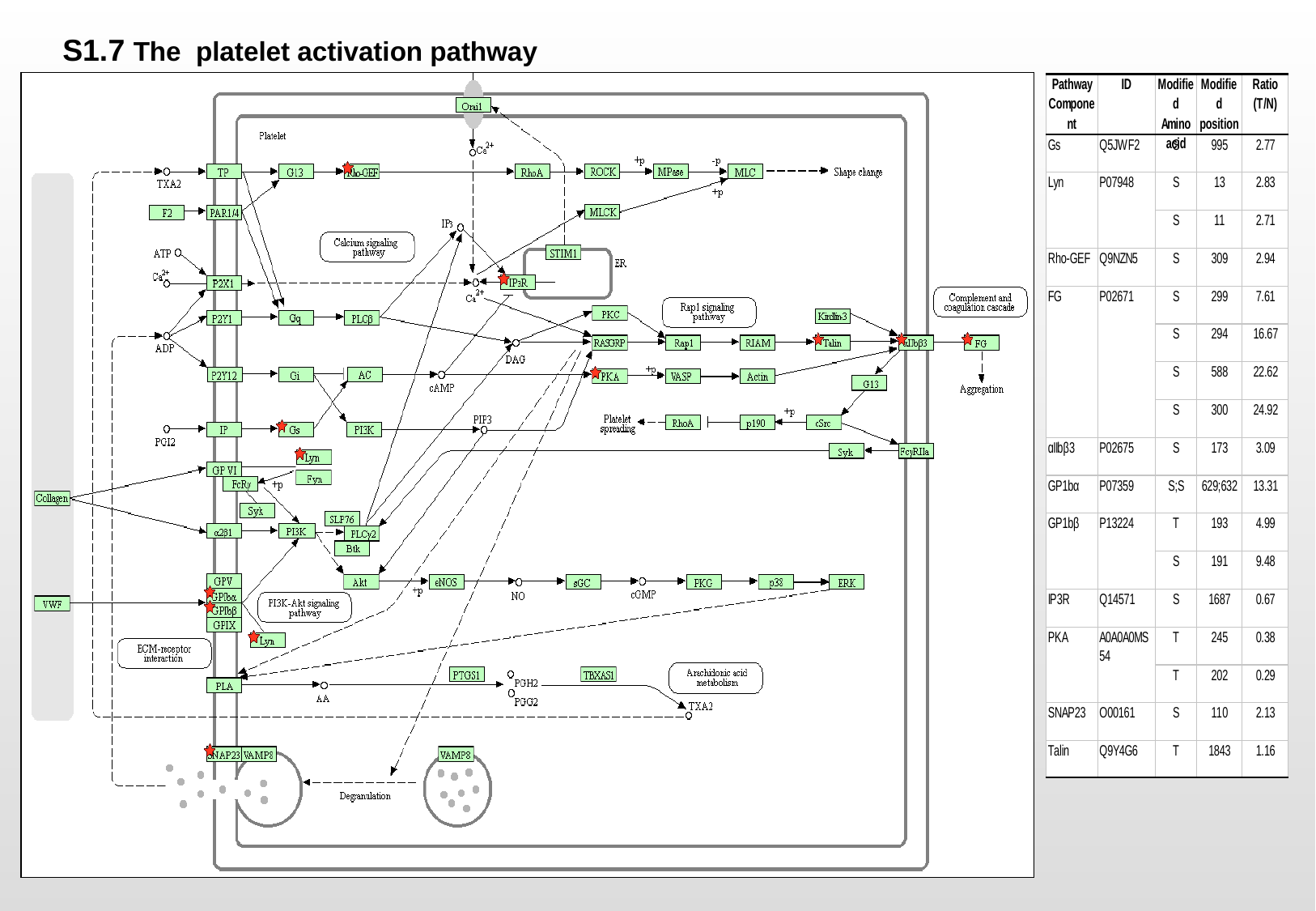

S1.7 The platelet activation pathway

## Slide 9
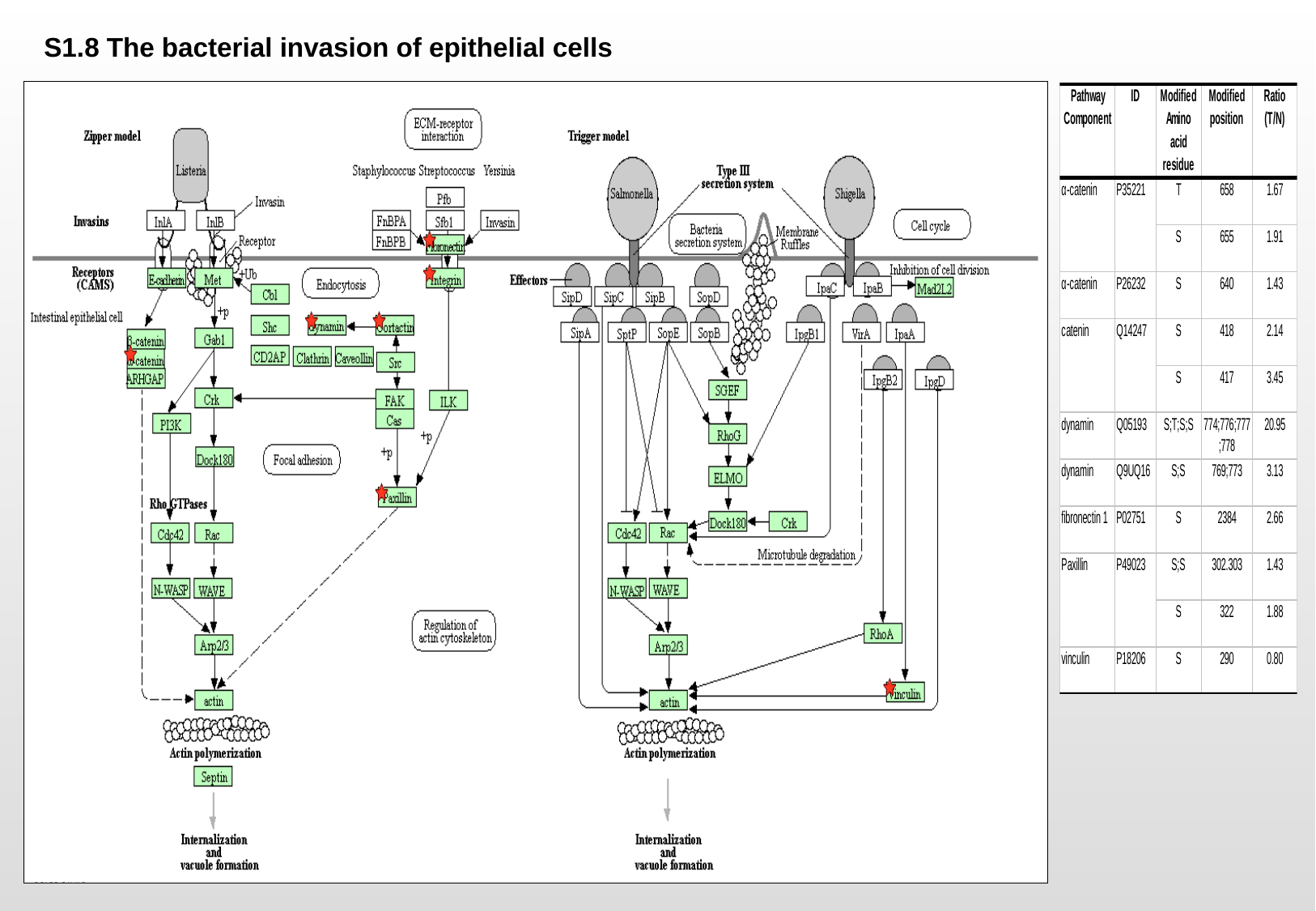

S1.8 The bacterial invasion of epithelial cells

## Slide 10
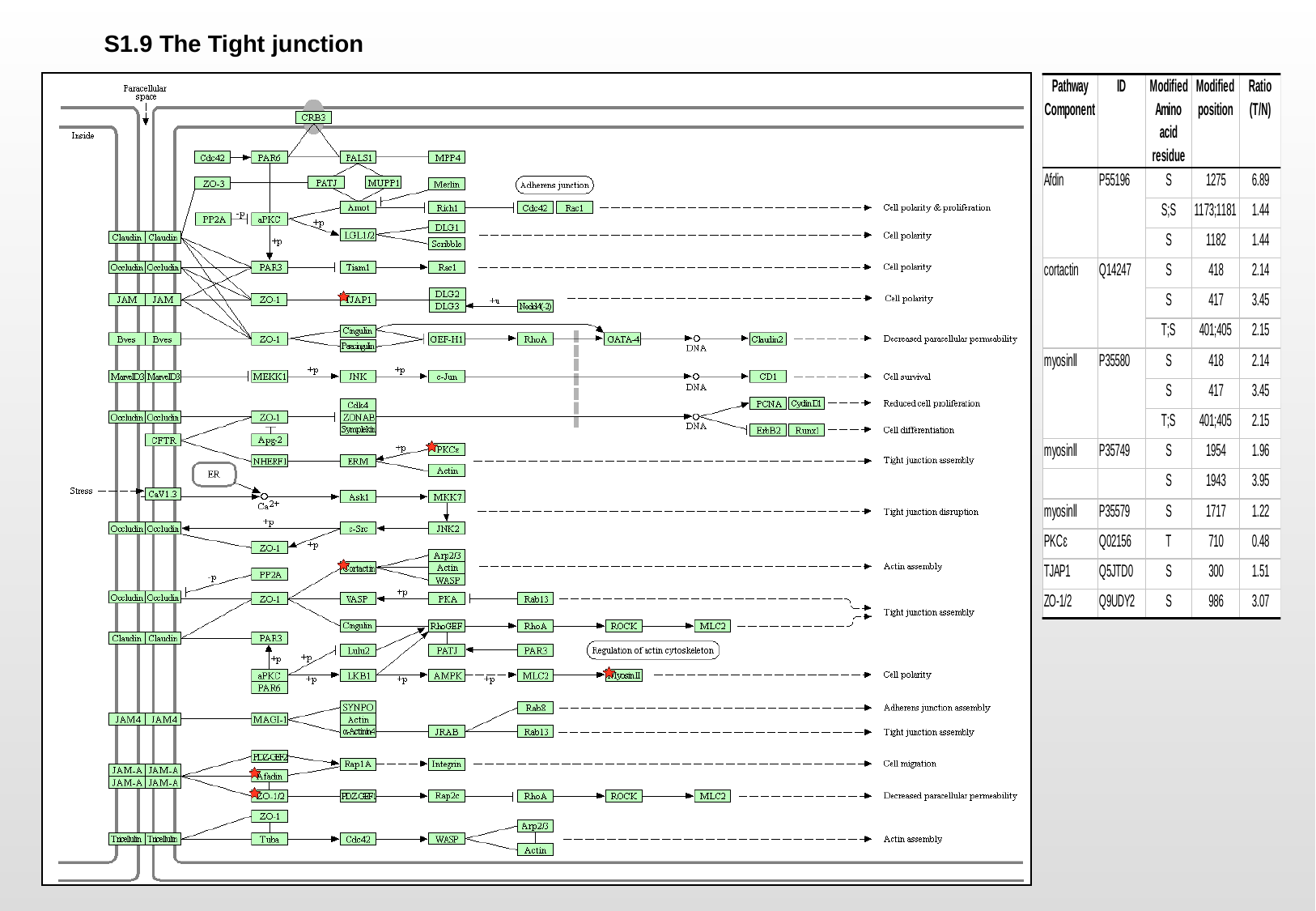

S1.9 The Tight junction

## Slide 11
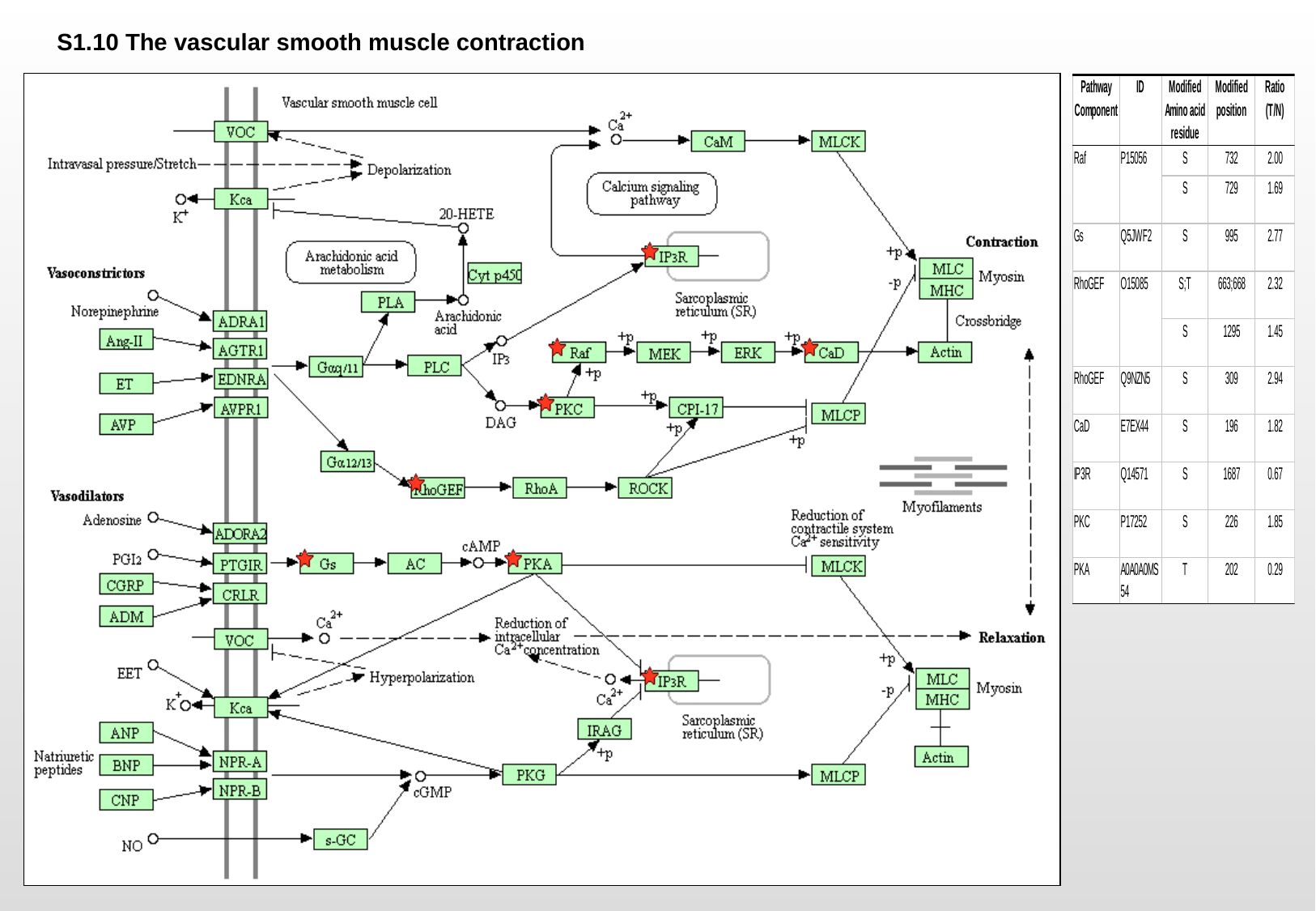

S1.10 The vascular smooth muscle contraction

## Slide 12
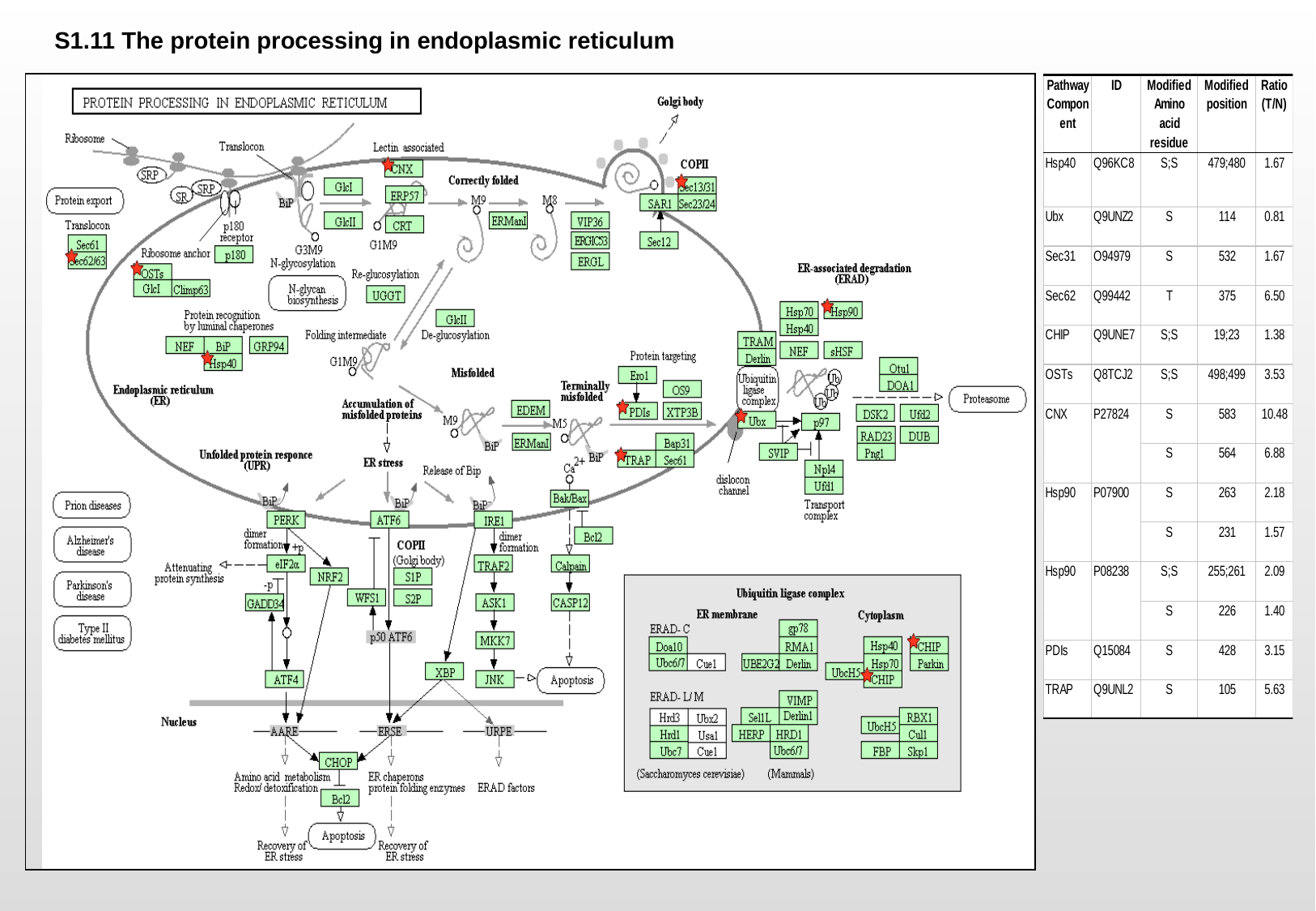

S1.11 The protein processing in endoplasmic reticulum
