# Supplementary material for: Phosphorylation-Mediated Molecular Pathway Changes in Human Pituitary Neuroendocrine Tumors Identified by Quantitative Phosphoproteomics
Source: Cells. 2021 Aug 27;10(9):2225. doi: 10.3390/cells10092225 (PMC8471408; doi:10.3390/cells10092225)
Supplement: Supplementary file 1 [file cells-10-02225-s001.zip › Table S1_v4.pdf]

Table S1. Clinical information of human nonfunctional PitNET and control tissue samples. DNT = do not test. ACTH = adrenocorticotropic hormone. GH = growth hormone. PRL = prolactin. FSH = follicle-stimulating hormone. LH =luteinizing hormone. TSH= thyroid-stimulating hormone. hGH = human growth hormone. NFPA = nonfunctional pituitary adenoma. IP = immunoprecipitation. WB = Western blot.

Group	Sex	Age	Clinical information	Immunohistochemistry	Experiments
NFPA	Male	58	NFPA in sellar region. Sellar floor bone destruction, enriched blood supply in tumor, and tumor size: 4.5 x 3 x 3 cm^3	ACTH (-), hGH (-), PRL (-), FSH (-), LH (-), TSH (-)	Proteomics
	Male	53	NFPA in sellar region. Sellar floor bone thinning, and tumor size: 3 x 3 x 2.5 cm^3	ACTH (-), hGH (-), PRL (-), FSH (-), LH (-), TSH (-)	Proteomics
	Female	43	NFPA in sellar region. Sellar floor bone thinning, enriched blood supply in tumor, and tumor size: 4 x 3 x 3 cm^3	ACTH (-), hGH (-), PRL (-), FSH (+), LH (-), TSH (-)	Proteomics
	Female	43	NFPA in sellar region. Adehesion of surrounding tissues, and tumoer size: 4.5 x 4 x6 cm^3	ACTH (-), hGH (-), PRL (-), FSH (+), LH (-), TSH (-)	Proteomics
	Female	51	NFPA in sellar region. Adehesion of surrounding tissues, and tumor size: 4 x 4 x3.5 cm^3	ACTH (+), hGH (-), PRL (-), FSH (+), LH (-), TSH (-)	IP/WB
	Female	43	NFPA in sellar region. Sellar floor bone thinning, enriched blood supply in tumor, and tumor size: 3.5 x 3 x 2.5 cm^3	ACTH (-), hGH (-), PRL (-), FSH (-), LH (-), TSH (-)	IP/WB
	Female	44	NFPA in sellar region. Sellar floor bone thinning, and tumor size: 2.5 x 2.9 x 1.8 cm^3	ACTH (-), hGH (-), PRL (-), FSH (-), LH (-), TSH (-)	IP/WB
Control	Male	36	White, Multiple toxic materials. Blood alcohol = 0.5	DNT	Proteomics; IP/WB

		g/L. Blood: HepB (+), HepC (−), HIV (−).		
Female		White, 15 h gunshot wound to head. No drugs or alcohol. Blood: HepB (−), HepC (−), HIV (−).	DNT	Proteomics; IP/WB
Female	34	Black, Gunshot wound to chest. Blood alcohol = 0.3 g/L; no drugs. Blood: HepB (+), HepC (−), HIV (−).	DNT	Proteomics; IP/WB
Female	40	White, Multiple toxic compounds. Blood: HepB (+), HepC (+), HIV(−).	DNT	Proteomics; IP/WB
Male	45	White, Drowning. Blood alcohol = 3.1 g/L; no other drugs detected. Blood: HepB (+), HepC (+), HIV(−).	DNT	IP/WB
