# Supplementary material for: Phosphorylation-Mediated Molecular Pathway Changes in Human Pituitary Neuroendocrine Tumors Identified by Quantitative Phosphoproteomics
Source: Cells. 2021 Aug 27;10(9):2225. doi: 10.3390/cells10092225 (PMC8471408; doi:10.3390/cells10092225)
Supplement: Supplementary file 1 [file cells-10-02225-s001.zip › Table S4_v1.pdf]

Table S4. Statistically significant GO cellular components (CC) of differentially phosphorylated proteins in human nonfunctional PitNETs.

ID	Cellular components	Count	%	PValue	Genes
GO:0005654	nucleoplasm	196	34.4	4.5E-33	P23193, Q15477, Q13200, A0A0A0MS54, Q5H9R7, O75494, O95391, O43491, P55196, Q96T37, O75554, Q92922, Q00839, Q08J23, Q9NTI5, Q9BZZ5, O95251, Q7Z6E9, Q9UKV3, Q12996, Q15459, Q9UEY8, Q9Y4W2, Q9Y6G9, P17252, O75376, Q9UDY2, Q8NFD5, O75475, Q96FV9, F8VXC8, Q96B36, Q6PKG0, Q12789, P42858, Q13098, Q5VT52, Q9NVM9, Q8NE71, O43395, P35611, Q14839, Q29RF7, A0A024R0Z3, B4DR36, Q92504, P52756, Q96T23, Q9BXP5, Q08170, Q13439, Q01130, Q9Y3T9, O75151, A0A024R2M8, P31943, Q6PJT7, Q15424, Q04727, Q13427, P09651, P18583, P49756, O95218, Q5VTR2, Q8NDX5, P05114, Q96PU4, P19338, P25788, O95104, O95400, Q14694, Q9C0C2, Q13547, Q14498, O60832, P07900, Q9H7L9, P83916, Q6NZI2, Q08999, Q99502, Q6KC79, P17096, P52948, J3KTL2, P07910, C9JFV4, P43243, Q9UNE7, O43493, Q15287, Q12906, P51531, P52701, Q9NYB0, Q9UQ35, Q8N7H5, Q14137, Q16629, Q7Z5K2, Q9Y3E1, Q9H4A6, Q9UQE7, O00161, Q02880, Q5VZL5, Q9H1B7, Q14676, O00567, A0A0S2Z4Z6, Q96EZ8, P02545, P08238, Q16637, Q53GS9, P49407, Q8WYP5, Q9NWH9, Q9Y2F5, O43823, O00461, Q9Y2W1, Q9HC78, Q96JM3, Q9UKN8, A0A0A0MR66, P51858, Q9P2I0, Q8WVC0, Q14980, Q13131, Q9Y618, B3KUY2, Q86VM9, Q13501, A0A140VK92, Q02040, Q1KMD3, P49023, A8MXP9, Q5JVS0, P22059, Q92614, Q16514, Q53EL6, O75400, Q16513, O43719, A0A0A0MRM9, Q96T60, Q9H0D6, Q92769, Q9NYF8, Q96C24, P18615, Q9UNZ2, Q9P2K5, Q92625, B4DY08, Q9H307, Q8IXM2, Q13242, Q9NXG2, Q13895, Q96ST2, Q13247, Q96T58, P49450, Q9Y5J1, Q13627, Q8NEY8, Q86XP3, O75533, Q96KR1, Q16352, Q9NYV4, A0A0A0MT33, Q5T200, P27708, P61266, Q92945, P29375, O14579, Q9BWU0, Q12888, O75643, P19634, Q7Z6Z7, Q13367, O96019, O00505, P35659, Q05519, Q14103, Q9HCN4, P35658, P29590

GO:0005829	cytosol	184	32.3	1.6E-18	Q15287, Q13200, Q13144, A0A0A0MS54, Q5H9R7, Q4KMP7, O75494, P82094, A0A0U4BW16, O43847, O60291, Q4VCS5, P55196, B7Z6G2, D6REX3, Q16623, Q5JSH3, O15027, Q13283, P49792, O60716, P50502, Q92539, Q7Z5K2, Q9Y3E1, Q9UQE7, Q9H4A6, O60504, P15056, D3DUW5, Q9NTI5, Q02880, P18669, E7EX17, A0A024R098, A0A0S2Z4Z6, P11277, Q07889, A0A140VKC4, Q7Z6E9, Q15149, P02545, Q9UKV3, P08238, Q16637, O75131, Q8N5V2, Q8WYP5, P49407, Q9UEY8, P34932, P18206, P17252, Q32P45, P16157, P35606, Q9UDY2, O60271, Q15121, Q6PCE3, O75475, Q9NZN5, O94979, Q8WWN9, Q96B36, P42858, O75122, Q14980, P07948, E9PRY8, Q13131, Q5JWF2, Q99996, Q8NE71, P60174, P05387, Q9ULH1, Q9UPQ9, P35612, P35611, P05388, B3KUY2, Q29RF7, Q13501, Q99442, Q59GJ0, Q641Q2, Q13085, P07814, Q6DEN2, P49023, Q13439, Q9UQN3, Q5JVS0, P22059, A0A024RAN2, Q53EL6, Q16513, Q92597, O15085, P26232, Q9NR45, Q06210, Q9UEW8, A0A087WUZ3, P35221, E9PDH4, Q01484, Q02156, P11166, Q9UNZ2, O75061, Q02410, Q9Y2H6, Q15036, Q9Y2X7, B4DY08, Q14194, Q13895, Q9NQT8, Q9H4G0, B7ZKY2, Q08495, P21333, P49450, Q9BTU6, O43237, Q07960, Q9Y4F1, Q9Y5B6, P35579, Q13813, P01185, A0A0G2JMX7, P35580, Q12955, P16949, O60841, A0A024RDB2, Q8NFP9, P17600, E7EX44, P24534, P04792, P46821, Q9C0C9, O94874, P25788, P49585, Q9H0B6, Q9C0C2, P68871, P23588, Q16555, Q13547, Q8IWW6, P07900, P08670, Q09666, P27708, P61266, O14576, Q92945, Q6ZRI8, P49840, O43765, Q53EW6, P35749, O14579, P63027, Q6NZI2, A0A024R2C5, P31323, Q9Y6D5, P17096, Q7Z6Z7, Q149N5, Q96KC8, O00505, P52948, Q9UN37, O60763, P07910, Q14103, O94921, P35658, Q9UNE7, O43493, P29590
GO:0016607	nuclear speck	37	6.5	3.4E-18	Q15287, Q9UK58, P80723, Q66PJ3, Q86XP3, Q9UBB9, O95400, Q9NYF8, O75533, O75494, O95391, Q9UQ35, Q96FV9, Q9Y2W1, Q9NYV4, Q14498, Q5T200, Q9H0G5, Q6PJT7, Q96T37, O75554, Q9H307, Q6PD62, Q13427, P18583, O43395, Q13247, P49756, A0A0S2Z4Z6, J3KTL2, P02545, Q02040, Q9UKV3, Q08170, O75400, Q13627, Q01130
GO:0005913	cell adherens junction	42	7.4	5.4E-15	Q9UHB6, Q9UPN3, P18206, Q9H0B6, A0A087WUZ3, A0A0U4BW16, Q9UDY2, P35221, A0A024R1S8, Q9C0C2, Q15762, P55196, Q6PKG0, Q09666, O60716, C9J6P4, P42166, A0A024R4E5, P07948, E9PRY8, Q9H4G0, Q9ULH1, P35611, Q9H2G2, P21333, Q07960, O00567, Q92522, P35579, Q13813,

					Q15149, O60763, P08238, O95292, Q9UQN3, Q9BY44, A0A024RAN2, Q16513, O76021, E7EX44, Q92597, P26232, Q14247
GO:0005737	cytoplasm	241	42.4	1.0E-14	Q9BVG4, Q5H9R7, O75494, O95391, P27816, P11137, A0A0U4BW16, Q9BUT9, O43491, P55196, H0YBE0, O60716, P50502, O43294, Q08J23, Q9BZZ5, Q8ND56, O95251, Q9UKV3, Q9BZH6, Q9UEY8, Q9Y4W2, Q9Y6G9, P17252, Q2M2I8, P16157, Q9UDY2, Q8NFD5, Q96FV9, Q9NZN5, Q8TEA8, Q96B36, Q6PKG0, P42858, Q13098, C9J6P4, Q02952, Q9NVM9, P07948, Q8WUY3, O43395, Q8NE71, P78559, Q8WX93, Q14839, B4DR36, Q9P035, Q13085, P07814, Q9BXP5, Q13439, Q9UQN3, P53805, O15085, Q9NR45, Q9Y3T9, Q9UEW8, O75151, P28290, A0A024R2M8, P31943, Q6PJT7, Q15036, P42166, Q13425, Q13428, Q13427, Q14194, P09651, A0A024R4E5, Q9NQT8, P21127, B7ZKY2, P49756, Q13107, P21333, Q9H2G2, Q07960, Q9Y4F1, Q15773, Q3KQU3, A0A0G2JMX7, P05114, O60841, O95747, O60941, P24534, P04792, P27105, Q9C0C9, O94874, P25788, O95400, Q14694, Q9C0C2, A0A0D9SF30, Q13547, O60832, P07900, Q9H7L9, P08670, Q09666, O43765, Q6NZI2, Q08999, Q9H1E3, P31323, Q99502, Q9NSK0, J3KTL2, Q9UN37, Q15435, C9JFV4, Q9Y3D3, Q9UNE7, Q15287, Q12906, Q9UPN3, Q08AD1, Q13144, P52701, Q9NYB0, O60291, Q4VCS5, Q8N7H5, D6REX3, O15027, Q14137, Q13283, Q16629, Q7Z5K2, Q9UQE7, Q9P2G1, P15056, O00161, D3DUW5, Q02880, Q5VZL5, P18669, O00567, P11277, Q96EZ8, Q15149, P02545, P08238, Q16637, O75131, P49407, Q9UHB6, Q9H2U2, P80723, Q32P45, O60271, P49006, Q9Y4G6, Q9UPT6, A0A024R1S8, O94979, Q8WWN9, P01189, Q96JM3, P48681, O75122, P51858, Q14980, E9PRY8, Q5JWF2, Q13131, Q8IV36, Q6ZTN6, P05388, B3KUY2, Q9UPU5, Q13501, Q14157, Q641Q2, Q02040, Q6DEN2, P49023, Q5JVS0, P12036, P22059, A0A024RAN2, Q7Z5L9, Q53EL6, O75400, Q16513, Q92597, Q14247, P26232, A0A0A0MRM9, Q92769, Q9UBB9, Q9NYF8, Q96C24, A0A087WUZ3, L0R530, P11171, Q02156, A0A024QZ15, Q9UNZ2, Q6GYQ0, Q9P2K5, Q92625, Q9Y2X7, Q8IXM2, O95359, Q13895, Q9H4G0, Q9BSQ5, P15924, P35579, P35580, O95292, P16949, A0A024RDB2, Q9BY44, Q9HBL0, Q8NEY8, Q496C9, P46821, P07359, Q86XP3, O95671, Q96KR1, Q16555, Q9Y2J2, P61266, O14576, P29375, Q8WXX5, Q9BWU0, A0A024R2C5, Q96K21, Q12888, P19634, P25490, Q86UE4, Q7Z6Z7, Q13367,

					O00505, C9JA93, O43166, Q9H2P0, O94921, Q9HCN4, P29590
GO:0005634	nucleus	239	42.0	2.8E-12	P23193, Q9UK58, Q15477, Q13200, Q96TC7, Q5JTV8, O75494, O95391, P82094, P85037, A0A0U4BW16, Q9BUT9, O43491, H0YBE0, O60716, Q92539, Q00839, O60504, Q9P2D1, Q08J23, Q9NTI5, Q9BZZ5, O95251, Q92522, Q7Z6E9, Q969E4, A0A140VKC4, Q9UKV3, Q9BZH6, Q13207, Q12996, Q9UK76, Q13595, O75376, P16157, O75475, Q96FV9, Q8TEA8, Q12789, P42858, C9J6P4, Q16849, Q7Z4V5, Q9NVM9, P07948, O43395, A0A090J7P6, P35611, Q8WX93, Q29RF7, Q14839, A0A024R0Z3, P20020, B4DR36, P52756, Q96T23, Q08170, Q9UQN3, P53805, Q01130, Q9Y3T9, Q9UEW8, Q9H6F5, O75151, P28290, A0A024R2M8, P31943, Q9BXK5, Q6PJT7, Q15424, Q04727, P42166, Q13428, P08651, Q13427, P09651, Q6P158, A0A024R4E5, P21127, Q13107, Q5VTR2, O95218, P21333, Q15773, Q8NDX5, Q9Y5B6, P05114, O60841, Q96PU4, Q9HCD5, P24534, P04792, P19338, Q9C0C9, O94874, P25788, Q8WUB8, O95400, Q14694, Q9C0C2, Q13547, Q14498, O60832, P07900, Q9H7L9, P83916, Q09666, O43765, Q6NZI2, Q08999, Q9H1E3, Q8ND76, Q99502, Q6KC79, P17096, J3KTL2, Q9UN37, Q15435, P07910, C9JFV4, P43243, Q15287, Q12906, P51531, Q13144, Q9NYB0, O60291, Q14137, P05455, Q13283, Q16629, Q7Z5K2, Q9Y3E1, Q9UQE7, P15056, Q02880, Q9H1B7, Q8TDD1, Q14676, Q3KNV8, A0A0S2Z4Z6, Q96EZ8, Q92733, P02545, P08238, Q16637, O75131, Q8WYP5, Q8WWQ0, P49407, P80723, Q9NWH9, Q32P45, P45880, O43823, Q9Y2W1, Q9HC78, Q96JM3, Q9H0G5, A0A0A0MR66, P53999, Q9UPA5, Q14980, E9PRY8, Q5JWF2, Q13131, P60174, Q9Y618, P05388, B3KUY2, Q02040, Q1KMD3, A8MXP9, Q5JVS0, P22059, Q13129, Q7Z5L9, Q53EL6, Q16513, O43719, Q92597, Q5SSJ5, P26232, Q96T60, Q66PJ3, Q9H0D6, Q92769, Q9NYF8, Q13185, A0A087WUZ3, L0R530, P11171, Q9GZT3, Q02156, P18615, P21796, Q9UNZ2, Q6GYQ0, Q9P2K5, Q02410, B4DY08, O95359, Q13895, Q96ST2, P15924, Q9BWW4, Q96T58, P49450, P17029, Q9Y5J1, P35579, P35580, O95365, Q13627, Q9NVU7, Q496C9, Q9BW71, Q9BRD0, Q86XP3, O75533, Q9NYV4, P27708, P61266, O14576, P29375, Q6IPX3, Q8WXX5, Q9BWU0, Q9NPJ4, A0A024R2C5, Q12888, P46100, O75643, P25490, Q86UE4, Q7Z6Z7, O96019, Q96KC8, O00505, P35659, Q9H2P0, Q05519, Q14103, O94921, P35658, P29590

GO:0005730	nucleolus	64	11.2	4.3E-11	Q12906, P23193, Q9Y3T9, Q96T60, Q9H6F5, Q66PJ3, Q9H0D6, O75151, Q9UBB9, Q9NYF8, A0A024R2M8, A0A087WUZ3, P11137, L0R530, H0YBE0, Q6PJT7, Q9H3N1, Q14137, Q13428, P08651, Q13242, O95359, Q9P2D1, Q13895, Q02880, Q08J23, B7ZKY2, Q8TDD1, Q5VTR2, P21333, O95251, O00567, Q92522, Q9Y5J1, Q96EZ8, Q7Z6E9, Q9UKV3, Q9NVU7, P19338, Q9UK76, Q9BW71, Q9Y4W2, Q13595, O43823, Q9NYV4, O60832, Q12789, P53999, O00264, P29375, Q8WVC0, E9PRY8, Q08999, A0A024R0Z3, Q8N8A6, B4DR36, Q86UE4, Q641Q2, O60763, Q13085, C9JFV4, P22059, Q9GZR7, O76021, P29590, A0A0A0MRM9
GO:0005925	focal adhesion	39	6.9	2.0E-10	Q9UHB6, P18206, Q96N67, Q32P45, P35221, A0A0U4BW16, Q9Y4G6, A0A024R1S8, O43491, B7Z6G2, Q13283, P08670, Q09666, O43294, O75122, P29966, Q9Y2X7, Q13425, Q02952, O60504, O00161, Q0ZGT2, B7ZKY2, P05387, P35611, Q8WX93, P05388, P21333, P19634, Q14676, P17096, Q9P035, P35579, Q15149, P24821, P49023, O75131, Q9HBL0, P04792, P35658, Q14247
GO:0016020	membrane	117	20.6	6.0E-10	Q12906, Q13200, O00203, O95391, A0A0U4BW16, O60291, P56730, Q8N7H5, P49792, P55011, O75694, O15320, Q00839, D3DUW5, P23634, P18669, Q9BZZ5, Q8TDD1, O00567, P08238, Q8TBA6, Q9BZH6, Q6ZSS7, Q8NC56, Q8N5V2, O60486, Q9Y4W2, Q9UEY8, Q9Y6G9, Q9UP95, O75376, O43823, Q9NZN5, Q14168, O00461, Q6PKG0, Q12789, O75122, O00264, Q9Y6V0, Q9P2I0, Q8TCJ2, Q5JWF2, Q8NE71, P27824, Q9ULH1, P05387, Q9Y618, P05388, Q14839, Q8N8A6, Q99442, P20020, B4DR36, P02724, Q92504, P07814, Q1KMD3, A8MXP9, A0A024RAN2, Q92614, P22059, Q9GZR7, O75400, O15085, Q96T60, Q9H0D6, Q14571, Q96C24, P31943, P21796, P11166, Q02410, Q9Y2H6, Q15036, Q9Y2X7, B4DY08, P42166, Q9H307, Q13425, Q5T1M5, P09651, O15173, Q13895, Q9BTU6, P21333, O43237, Q15773, P35579, Q13813, P24821, P16949, P19338, P07359, P27105, O94874, Q86XP3, Q9H0B6, O95674, A0A0D9SF30, Q16555, P07900, Q9BZC7, P27708, Q09666, Q92945, P61266, O43765, P63027, O75643, Q9Y6D5, Q7Z6Z7, Q9Y2Q0, Q149N5, Q96KC8, O60763, O75396, P07910, C9JFV4, O76021, P43243

GO:0005681	spliceosomal complex	18	3.2	2.5E-09	O43395, O75643, Q9UBB9, P49756, Q9BZZ5, O75533, Q96I25, O95391, P52756, Q92733, A0A0A0MT33, Q02040, P07910, O75554, B4DY08, Q53GS9, Q15459, P09651, Q01130
GO:0043234	protein complex	36	6.3	3.4E-08	O94874, P18206, Q92769, Q9H0B6, A0A087WUZ3, Q14694, A0A0U4BW16, P11171, F8VXC8, Q16555, Q13547, B7Z6G2, Q96B36, P07900, P42858, P27708, P50502, Q15036, B4DY08, Q92922, Q13425, D3DUW5, Q6NZI2, O43395, P27824, Q9BSQ5, O94885, P23634, Q9BWW4, Q99502, Q14839, Q9BTU6, Q96I25, O96019, P11277, P35579, P07910, Q16513
GO:0005856	cytoskeleton	33	5.8	9.4E-08	Q9UEY8, P27105, Q9UPN3, P80723, Q9UEW8, P18206, P16157, P11171, Q9C0C2, Q01484, Q02156, Q16555, O43491, P08670, Q9Y2J2, O43294, Q9Y6V0, Q02952, O60504, Q9H4G0, Q0ZGT2, Q99996, P15924, P35612, P35611, Q9Y4F1, Q15149, O43166, P49023, Q9HBL0, E7EX44, P04792, Q14247
GO:0016363	nuclear matrix	16	2.8	1.9E-07	Q9Y618, B7ZKY2, O43823, P25490, Q96FV9, A0A0S2Z4Z6, A0A0A0MT33, A8MXP9, P27708, O43294, O75400, Q13427, P43243, Q9UQE7, Q8WYP5, Q14980, P29590
GO:0000785	chromatin	15	2.6	3.9E-07	Q9NTI5, Q9H1E3, Q9Y618, Q29RF7, Q13185, Q6KC79, P17096, Q13547, P05114, P83916, P42166, Q7Z5K2, Q9UQE7, P49407, Q8WYP5
GO:0014731	spectrin-associated cytoskeleton	6	1.1	1.2E-06	Q12955, Q08495, P16157, A0A087WUZ3, P11171, P11277
GO:0030529	intracellular ribonucleoprotein complex	17	3.0	3.3E-06	Q12906, O75643, P05388, Q32P45, Q9GZT3, P07814, P07910, Q9H0G5, Q13283, P05455, Q12789, Q14137, A0A024RDB2, B4DY08, Q13627, Q14103, Q00839, P09651, P19338

GO:0071013	catalytic step 2 spliceosome	14	2.5	3.6E-06	O75643, Q9UBB9, O75533, O95391, A0A024R0Z3, Q9UQ35, P31943, A0A0S2Z4Z6, J3KTL2, P07910, Q9H307, B4DY08, Q00839, Q15459, P09651
GO:0014069	postsynaptic density	19	3.3	1.1E-05	Q9UEY8, Q9UQ16, P35612, Q08495, A0A087WUZ3, P27816, Q07889, A0A0G2JMX7, O43166, O75061, Q9Y2J2, P12036, P17600, Q9Y6V0, Q9UPA5, P46821, P49407, P07948, P26232
GO:0005794	Golgi apparatus	49	8.6	3.0E-05	Q9UPN3, P52701, O00203, Q8TBE7, P82094, P35221, P22466, Q02156, Q5JSH3, O15027, O00391, Q02410, Q9Y2H6, Q15036, Q9H4A6, D3DUW5, Q9BTU6, Q9NTI2, Q12955, O95292, Q8TBA6, P17600, Q8NEY8, Q6IAA8, Q9UQ16, O43823, O75379, O00461, Q05193, P42858, C9J6P4, O75122, Q16849, Q9NQQ7, O14579, P07948, Q8IV36, Q99996, Q8WXF7, Q13367, Q9Y2Q0, Q92504, A0A140VK92, O60763, Q13439, Q92614, P22059, Q14247, O43493
GO:0005875	microtubule associated complex	8	1.4	5.6E-05	A0A0G2JMX7, P78559, P49023, P49792, P11137, P27816, Q15121, P46821
GO:0030141	secretory granule	11	1.9	7.0E-05	P10645, P63027, Q16623, P05060, C9J650, P05408, Q96C24, Q16849, P13521, P22466, P01189, P01185
GO:0036464	cytoplasmic ribonucleoprotein granule	7	1.2	7.7E-05	A0A0G2JMX7, Q16637, P05388, O14576, Q00839, P19338, Q16352
GO:0005938	cell cortex	14	2.5	8.4E-05	Q9UEY8, P02671, Q14571, P02675, B7Z6G2, P35580, P49023, Q5TBA9, O75122, P29966, Q02952, Q14980, P19338, Q14247
GO:0008091	spectrin	5	0.9	9.0E-05	A0A087WUZ3, P11171, P11277, O43491, Q13813
GO:0043005	neuron projection	20	3.5	1.0E-04	P63027, O00161, O94874, P23634, Q96N67, Q9BTU6, P27816, P11137, Q01484, Q13547, B7Z6G2, Q16623, P07900, Q12955, P16949, Q02410, Q16637, P08670, Q92625, P15056

GO:0015629	actin cytoskeleton	19	3.3	1.1E-04	Q9UHB6, P18206, B7ZKY2, Q8WX93, Q08495, P21333, A0A0U4BW16, P35221, Q9Y4G6, P11277, Q8NDX5, P35579, Q96EZ8, P35580, Q13085, Q09666, Q9HCD5, P29966, E7EX44, P26232
GO:0045111	intermediate filament cytoskeleton	9	1.6	1.5E-04	P51531, P48681, Q15149, Q9UNZ2, P08670, Q99442, Q16513, Q16352, Q9UNE7
GO:0043034	costamere	6	1.1	2.0E-04	O15061, Q15149, P18206, Q12955, Q09666, Q01484
GO:0043197	dendritic spine	12	2.1	2.0E-04	P35580, P27824, O43166, P31323, Q9UQ16, Q9ULH1, O60716, Q9Y6D5, Q9Y4F1, P49407, P46821, Q14247
GO:0005694	chromosome	12	2.1	3.1E-04	Q7Z6E9, Q9NTI5, Q15435, Q9UNZ2, Q7Z5K2, Q29RF7, Q14676, Q96KR1, Q9UQE7, Q8NEY8, Q5SSJ5, Q14980
GO:0071564	npBAF complex	5	0.9	3.3E-04	P51531, Q8WUB8, Q92922, O96019, F8VXC8
GO:0030863	cortical cytoskeleton	6	1.1	4.1E-04	Q08495, P21333, A0A0U4BW16, P11171, P35579, P02730, Q14247
GO:0008021	synaptic vesicle	11	1.9	4.3E-04	P63027, Q05193, Q16623, A0A0A0MS29, Q96A57, Q02410, P61266, P45880, P17600, Q16849, P21796
GO:0005874	microtubule	22	3.9	4.9E-04	D3DUW5, Q9NQT8, Q9UPN3, Q9Y6G9, Q96TC7, Q08AD1, Q9UQ16, P78559, Q9H0B6, P27816, P11137, O43237, Q9NSK0, Q16555, A0A0G2JMX7, Q05193, P16949, O75122, O14576, Q13425, Q92597, P46821
GO:0008180	COP9 signalosome	7	1.2	5.5E-04	P80723, P08238, Q13098, Q96N67, A0A0U4BW16, Q4VCS5, P35579, O43491

GO:0070062	extracellular exosome	114	20.0	5.5E-04	Q13200, B2RUZ4, A0A0A0MS54, P27816, A0A0U4BW16, O60291, P02730, O43491, O60716, P50502, P55011, Q16629, O00161, P18669, Q15149, P08238, O75131, Q8WWQ0, Q8WYP5, Q9H2U2, P34932, P80723, P18206, Q9UQ16, P17252, P45880, O60271, P49006, Q9NZN5, A0A024R1S8, Q9Y2W1, Q05193, P53999, P01008, O00264, Q9Y6V0, Q14980, P07948, Q8IV36, Q5JWF2, P02671, P27824, P60174, P05387, B3KUY2, P05388, A0A024R0Z3, Q13501, P20020, P02724, A0A140VK92, Q15084, Q13085, Q13439, Q9UQN3, Q92597, Q01130, Q14247, Q9NR45, Q06210, A0A024R2M8, A0A087WUZ3, P21796, P11166, O00391, B4DY08, Q13425, P09651, P15924, P01042, P02675, Q96T58, Q9H2G2, P21333, Q07960, P35579, Q13813, P35580, B7Z351, Q9NRX5, P16949, O95747, P19338, P04792, P07359, Q6IAA8, P27105, P02751, P25788, P68871, A0A0D9SF30, Q16555, P07900, P08670, P27708, Q09666, P29966, P35749, P63027, Q08999, A0A024R2C5, Q8ND76, P31323, P02765, Q6KC79, P19634, Q7Z6Z7, Q9Y2Q0, C9JA93, Q9UN37, J3KTL2, Q14515, Q15435, P07910, Q14103, Q9UNE7
GO:0030133	transport vesicle	11	1.9	5.6E-04	Q5JWF2, O75396, O60763, Q8TBA6, P35606, O75379, O15240, O14579, O43493, A0A140VK92, O00461
GO:0016514	SWI/SNF complex	5	0.9	8.4E-04	P51531, Q92922, Q8NFD5, O96019, F8VXC8
GO:0016323	basolateral plasma membrane	15	2.6	1.1E-03	Q9UEW8, P15924, B7ZKY2, P23634, P16157, P19634, P20020, Q01484, P02730, P11166, P07900, Q12955, P08238, P49407, P26232
GO:0048471	perinuclear region of cytoplasm	34	6.0	1.1E-03	P10645, P27105, Q9UQ16, P17252, A0A0A0MS54, E9PDH4, O60271, Q9GZT3, O94979, Q02156, D6REX3, Q13547, H0YBE0, P07900, Q5JSH3, Q02410, O14576, P07948, D3DUW5, Q5JWF2, P63027, P31323, Q08495, Q9Y6D5, Q9H2G2, P21333, P19634, Q86UE4, Q07960, Q9UN37, B7Z351, O60763, P02545, P22059, Q16513, Q92597

GO:0030054	cell junction	27	4.7	1.6E-03	F5H7N0, P80723, Q9UDY2, P35221, P11171, Q8N7H5, O43491, P55196, Q16623, Q16849, Q9Y6V0, Q9UPA5, P63027, D3DUW5, Q96A57, P15924, Q9Y6D5, Q9BTU6, Q9Y4F1, Q96EZ8, A0A0A0MS29, O43166, O75131, P22059, P17600, Q16513, P46821
GO:0005911	cell-cell junction	14	2.5	2.1E-03	P11166, O00161, P55196, Q9UEY8, Q9H4G0, P18206, B7ZKY2, O60716, Q9Y2J2, Q9H307, P21333, E9PDH4, P35221, Q9Y4G6
GO:0030426	growth cone	11	1.9	2.6E-03	A0A0G2JMX7, P35580, P80723, Q6DEN2, Q8WX93, O60716, Q96N67, Q8N5V2, Q5T1M5, Q16555, Q14247
GO:0005802	trans-Golgi network	12	2.1	2.7E-03	P63027, Q96A57, Q5JTD0, Q13439, Q8NFP9, O75122, Q92614, Q9Y6D5, Q9H4A6, Q9Y2Q0, O43493, Q9NTI2
GO:0030018	Z disc	11	1.9	2.9E-03	Q0ZGT2, Q12955, Q8WX93, P23634, Q16637, P21333, P16157, P04792, Q01484, P02730, Q9UNE7
GO:0030424	axon	16	2.8	3.0E-03	Q9NQT8, P27824, Q9UQ16, Q8WXF7, Q8WX93, P07197, Q96N67, P56730, A0A0G2JMX7, P35580, P42858, Q9H2P0, P12036, Q5T1M5, Q9UPA5, P26232
GO:0043231	intracellular membrane-bounded organelle	30	5.3	3.1E-03	Q6IAA8, P51531, Q13595, P52701, O95391, P35221, O94979, D6REX3, O00391, Q15036, Q7Z5K2, Q9H4A6, O15240, P15056, Q13895, D3DUW5, Q9BWU0, P63027, Q99996, P23634, P20020, Q9Y2Q0, Q8ND56, Q8NDX5, A0A140VK92, Q13813, Q641Q2, Q13439, Q8NEY8, P35658, Q14247
GO:0005819	spindle	11	1.9	3.5E-03	Q96JM3, P35580, Q08J23, P83916, P61266, Q13185, A0A0U4BW16, Q14194, Q14980, P04792, P35579, Q3KQU3
GO:0005720	nuclear heterochromatin	5	0.9	3.8E-03	P83916, Q96PU4, P46100, Q13185, O75475

GO:0045121	membrane raft	15	2.6	3.8E-03	Q6NZI2, Q6IAA8, P27105, P18206, P31323, P45880, Q9BTU6, P19634, E9PDH4, P20020, Q15762, Q01484, P21796, Q09666, P07948
GO:0042641	actomyosin	4	0.7	4.8E-03	Q16623, P35580, Q92614, A0A0U4BW16, P35579
GO:0016580	Sin3 complex	4	0.7	6.1E-03	Q13547, Q9H7L9, Q92769, O75376
GO:0072562	blood microparticle	12	2.1	6.2E-03	P11166, P02671, P02751, P27105, P01042, P02675, A5PL32, P02765, Q9BY44, P01008, P68871, P02730
GO:0043209	myelin sheath	12	2.1	6.2E-03	Q05193, P07900, P27824, P18669, P12036, P45880, P17600, P10515, Q92597, Q16352, P21796, Q16555
GO:0000775	chromosome, centromeric region	7	1.2	7.1E-03	Q9NTI5, P83916, Q13185, Q7Z5K2, Q29RF7, P49450, Q9UQE7
GO:0071565	nBAF complex	4	0.7	7.6E-03	P51531, Q92922, Q8NFD5, F8VXC8
GO:0097418	neurofibrillary tangle	3	0.5	8.5E-03	A0A0G2JMX7, P07197, P12036
GO:0030027	lamellipodium	12	2.1	9.0E-03	B7Z6G2, P35580, Q6DEN2, Q8WX93, P49023, O60716, P19634, P35221, Q16513, Q4VCS5, P26232, Q14247
GO:0005903	brush border	7	1.2	9.0E-03	Q9UHB6, D3DUW5, Q9UEY8, P35580, Q15149, P18206, A0A0U4BW16, P35579
GO:0019013	viral nucleocapsid	5	0.9	9.3E-03	P07910, O75643, Q09666, B4DY08, Q14103, P09651
GO:0030687	preribosome,	5	0.9	1.1E-02	Q9Y4W2, Q9H6F5, P05387, Q14137, P05388

	large subunit precursor				
GO:0030673	axolemma	4	0.7	1.1E-02	Q9Y2J2, P16157, A0A087WUZ3, Q9UPT6
GO:0000790	nuclear chromatin	13	2.3	1.4E-02	P51531, Q9H1E3, Q9Y618, P52701, Q92769, O75376, Q14839, O96019, F8VXC8, Q13547, P07910, P16402, B4DY08, Q92922
GO:0005652	nuclear lamina	3	0.5	1.7E-02	P02545, B7ZKY2, P61266
GO:0016593	Cdc73/Paf1 complex	3	0.5	1.7E-02	Q6PD62, Q8WVC0, Q8N7H5
GO:0005815	microtubule organizing center	11	1.9	1.7E-02	Q9Y4W2, Q14498, Q7Z6E9, Q9BZC7, Q5TBA9, O75122, P61266, Q9Y6D5, O60271, Q14194, O95359
GO:0015030	Cajal body	6	1.1	1.8E-02	O43395, Q86XP3, Q9Y2F5, Q16637, Q9UQ35, A0A0A0MRM9
GO:0031201	SNARE complex	6	1.1	2.1E-02	O00161, P63027, Q16623, O75396, P61266, O75379
GO:0031965	nuclear membrane	14	2.5	2.1E-02	Q86UE4, Q9P035, P52948, Q96KC8, Q16352, H0YBE0, P02545, P49792, O75694, P42166, Q96T37, Q8NC56, Q8WYP5, Q9UK76, P29590
GO:0005635	nuclear envelope	11	1.9	2.2E-02	Q8NE71, P02545, P49585, P49792, O75694, Q13185, P42166, L0R530, Q9NYB0, P52948, O15173
GO:0005883	neurofilament	3	0.5	2.2E-02	P07197, P12036, Q16352
GO:0001939	female pronucleus	3	0.5	2.2E-02	P11166, P83916, O43823
GO:0034399	nuclear periphery	4	0.7	2.4E-02	A0A0G2JMX7, O75475, Q8WYP5, P52948
GO:0005697	telomerase	4	0.7	2.4E-02	O60832, P07910, B3KUY2, B4DY08, Q00839

	holoenzyme complex				
GO:0031093	platelet alpha granule lumen	6	1.1	2.4E-02	P02671, P02751, O00391, P01042, P02675, P02765
GO:0030425	dendrite	18	3.2	2.5E-02	Q5JWF2, P05388, E7EX17, O75494, Q9BTU6, Q9Y4F1, Q9UPT6, P56730, P01185, P23588, Q16555, Q96EZ8, A0A0G2JMX7, Q12955, P42858, Q9H2P0, P17600, Q9UPA5, Q14980
GO:0030315	T-tubule	5	0.9	2.6E-02	Q12955, P23634, Q09666, P19634, Q01484
GO:0030658	transport vesicle membrane	5	0.9	2.6E-02	P10645, Q96C24, Q16849, Q13425, Q8WXD2
GO:0035145	exon-exon junction complex	4	0.7	2.7E-02	Q15287, Q9H307, J3KTL2, Q9Y2W1
GO:0005577	fibrinogen complex	3	0.5	2.8E-02	P02671, P02751, P02675
GO:0005915	zonula adherens	3	0.5	2.8E-02	P18206, O60716, P35221
GO:0031252	cell leading edge	5	0.9	2.9E-02	P08670, O75122, Q9H2G2, Q2M2I8, A0A0U4BW16, P35579
GO:0043025	neuronal cell body	17	3.0	2.9E-02	P27824, Q99996, P31323, E7EX17, O75494, Q9BTU6, P21333, P22466, Q07889, Q16555, P23588, B7Z6G2, P35580, P07900, P27708, Q9H2P0, Q16849, Q14980
GO:0035327	transcriptionally active chromatin	4	0.7	3.0E-02	C9JFV4, Q9Y2F5, O75475, Q6PD62, B4DR36

GO:0042470	melanosome	8	1.4	3.2E-02	P11166, P27105, Q15084, O75396, P07900, P27824, P08238, P35749
GO:0031012	extracellular matrix	16	2.8	3.4E-02	P02751, P27824, P15924, P02765, P21333, A0A0U4BW16, P35579, P48681, Q15149, P24821, P02545, P07900, P08670, O43294, Q00839, P04792, Q15388
GO:0019898	extrinsic component of membrane	7	1.2	3.7E-02	Q9UEW8, Q9H4G0, Q9Y2J2, Q96C24, P11171, Q9Y4F1, O43491
GO:0005637	nuclear inner membrane	5	0.9	3.9E-02	Q5JTV8, A8MXP9, Q13185, P42166, Q9Y6X4, P43243
GO:0005768	endosome	13	2.3	4.0E-02	A0A024R2C5, Q9BZC7, Q5JTD0, Q9UQN3, Q15036, Q96C24, Q16849, Q9BTU6, O75379, Q9H4A6, Q9UN37, O43493, Q9NTI2
GO:0005884	actin filament	6	1.1	4.5E-02	Q08495, Q8WX93, P21333, Q5T1M5, Q4VCS5, Q14247
GO:0014704	intercalated disc	5	0.9	4.5E-02	P15924, Q12955, P19634, P35221, Q01484
GO:0000922	spindle pole	8	1.4	4.6E-02	A0A024R2C5, Q9Y6G9, Q96TC7, Q5TBA9, O14576, Q9UQE7, Q14980, Q9UN37
GO:0031519	PcG protein complex	4	0.7	4.6E-02	Q3KNV8, P25490, Q14157, Q8NDX5
GO:0000777	condensed chromosome kinetochore	7	1.2	4.7E-02	Q96JM3, Q9Y6G9, Q12888, O75151, O75122, O14576, Q8WYP5
GO:0031224	intrinsic component of membrane	3	0.5	4.9E-02	Q5JWF2, A5PL32, Q9BTU6
GO:0044615	nuclear pore nuclear basket	3	0.5	4.9E-02	P49792, P35658, P52948

GO:0033270	paranode region of axon	3	0.5	4.9E-02	Q9NQT8, Q12955, Q9Y2J2
