# Supplementary material for: Phosphorylation-Mediated Molecular Pathway Changes in Human Pituitary Neuroendocrine Tumors Identified by Quantitative Phosphoproteomics
Source: Cells. 2021 Aug 27;10(9):2225. doi: 10.3390/cells10092225 (PMC8471408; doi:10.3390/cells10092225)
Supplement: Supplementary file 1 [file cells-10-02225-s001.zip › Table S5_v2.pdf]

Table S5. Statistically significant GO molecular functions (MF) of differentially phosphorylated proteins in human nonfunctional PitNETs.

ID	Molecular functions	Count	%	PValue	Genes name
GO:004482	poly(A) RNA binding	146	25.7	4.9E-51	Q12906, Q15287, Q9UPN3, O75494, P27816, A0A0U4BW16, Q9UQ35, H0YBE0, Q14137, P05455, Q13283, Q16629, Q96T37, Q00839, Q08J23, Q9BZZ5, Q8TDD1, E7EX17, O00567, Q8ND56, A0A0S2Z4Z6, Q92522, A0A140VKC4, Q7Z6E9, Q15149, Q9UKV3, P08238, Q8N1G4, P16402, O75131, Q12996, Q15459, Q9Y4W2, Q9Y6G9, Q9NWH9, Q13595, Q32P45, O43823, O75475, Q9UPT8, Q9Y2W1, Q05193, Q6PKG0, Q9H0G5, A0A0A0MR66, P53999, C9J6P4, P51858, O43395, Q8NE71, P27824, Q9UPQ9, P35611, P05388, Q86VM9, A0A024R0Z3, Q8N8A6, B4DR36, Q13442, Q59GJ0, Q14157, P52756, Q02040, Q9BXP5, Q1KMD3, Q08170, A8MXP9, A0A024RAN2, Q92614, Q9GZR7, O75400, Q5VTL8, Q16513, O43719, P55081, Q01130, A0A0A0MRM9, Q9Y3T9, Q9H6F5, Q9H0D6, Q66PJ3, Q92769, Q9NYF8, A0A087WUZ3, P35221, P31943, Q9GZT3, P18615, Q6PJT7, Q5JSZ5, Q9BRJ6, Q15424, Q9P2K5, Q9Y2H6, B4DY08, Q9H307, Q13425, Q13428, Q13242, Q13427, Q9NXG2, P09651, A0A024R4E5, Q6P158, Q13895, P21127, P18583, P15924, Q13247, P49756, Q96T58, O95218, P21333, P35579, Q9Y5J1, Q8WXD2, O60841, A0A024RDB2, Q9HCD5, Q9HBL0, Q8NEY8, P19338, P04792, Q9C0C9, Q9BRD0, Q86XP3, O95104, O75533, Q14694, Q96KR1, P23588, Q14498, P07900, O60832, Q5T200, Q9UKJ3, Q09666, Q92945, Q6NZI2, Q9H1E3, O75643, Q86UE4, Q7Z6Z7, J3KTL2, O60763, P35659, P07910, Q7L4I2, C9JFV4, Q05519, O76021, Q14103, P43243

GO:0005515	protein binding	412	72.4	2.1E-33	Q13200, Q96TC7, Q5JTV8, Q4KMP7, Q5H9R7, O75494, P27816, P85037, A0A0U4BW16, Q9BUT9, P55196, H0YBE0, P50502, O75694, O15320, O75554, Q92922, Q00839, Q9P2D1, Q9NTI5, Q9BZZ5, A0A024R098, A0A140VKC4, Q13207, Q9Y6G9, O75376, P16157, Q2M2I8, O75379, O75475, Q96FV9, Q05193, Q96B36, Q6PKG0, P01008, Q16849, P07948, Q8NE71, P27824, Q9ULH1, A0A024R0Z3, B4DR36, Q92504, P52756, P07814, Q08170, Q13439, P55081, O15085, Q01130, A0A0S2Z5H6, Q9H6F5, O75151, P35221, P31943, Q01484, Q04727, Q13425, Q13428, Q13427, Q14194, O15173, A0A024R4E5, P21127, P18583, P01042, Q13107, P21333, Q9H2G2, Q07960, Q8NDX5, Q5XUX1, O15061, Q96PU4, Q9HCD5, O95747, P24534, P04792, P19338, P27105, P25788, Q96D71, O95400, Q13547, P07900, Q9H7L9, P08670, Q08999, Q8ND76, P31323, Q99502, Q9Y6D5, Q6KC79, Q9NSK0, J3KTL2, P07910, C9JFV4, Q9Y3D3, Q9UNE7, Q15287, P51531, Q9UPN3, Q08AD1, Q13144, P52701, Q9NYB0, O60291, Q8N7H5, Q4VCS5, P02730, Q16623, O15027, Q13283, Q16629, Q7Z5K2, Q6PD62, Q9H4A6, D3DUW5, Q5VZL5, O00567, P50479, P11277, A0A0S2Z4Z6, Q15149, Q92733, P02545, Q16637, Q8WWQ0, O60486, P80723, Q9UQ16, Q9Y2F5, Q9Y608, P45880, O60271, Q15121, P49006, Q9Y4G6, Q9UPT6, C9J650, P53999, Q14980, Q13131, P02671, Q16799, P05060, P60174, Q9UPQ9, Q9Y618, P07197, A0A140VK92, Q6DEN2, P49023, Q5JVS0, Q92614, P22059, Q13129, Q16514, O75400, Q53EL6, Q16513, Q5SSJ5, Q96T60, Q92769, Q9UBB9, Q9NYF8, Q13185, A0A087WUZ3, P22466, P11171, P11166, Q92625, Q13242, Q5T1M5, Q15388, Q13895, Q96ST2, Q9H4G0, Q9BSQ5, Q13247, Q96T58, Q9BTU6, P16949, A0A024RDB2, P17600, Q9HBL0, E7EX44, Q8NEY8, P02751, O75533, P68871, Q16555, Q5T200, Q9Y2J2, Q92945, F5H757, P29375, P49840, A0A024R2C5, O75643, Q12888, Q96I25, P13521, P25490, Q96KC8, O00505, Q9HCN4, P29590, P23193, Q9UK58, A0A0A0MS54, P11137, P82094, O95391, Q9Y5Y0, B7Z6G2, P49792, O60716, O43294, Q96T37, O60504, E7EX17, P10515, O95251, Q7Z6E9, Q9UKV3, Q9BZH6, Q8N1G4,

Q12996, Q15459, P18206, P17252, P35606, Q9UDY2, Q8NFD5, F8VXC8, Q9NZN5, Q12789, P42858, Q13098, C9J6P4, Q7Z4V5, Q02952, Q9NVM9, O43395, Q99996, P56597, P78559, Q8WX93, P41236, Q29RF7, Q14839, Q9P035, P20020, Q15084, Q13085, Q96T23, Q9BXP5, Q9UQN3, Q9Y3T9, Q9UEW8, A0A024R2M8, Q15762, Q9BXK5, Q15424, Q5JSZ5, Q6PJT7, Q9BRJ6, Q15036, P05408, P09651, Q6P158, Q9NQT8, B7ZKY2, P02675, Q08495, P49756, Q5VTR2, O95218, Q15773, Q13813, A0A0G2JMX7, B7Z351, Q12955, Q9NRX5, O60841, O60941, Q6IAA8, Q9C0C9, O94874, P13224, Q14694, P23588, Q14498, O60832, P83916, Q09666, O43765, Q53EW6, Q6NZI2, P17096, Q9Y2Q0, P52948, Q9UN37, Q15435, P43243, O43493, Q12906, O43847, D6REX3, Q14137, P05455, P55011, Q9UQE7, Q9P2G1, P15056, O00161, P18669, P23634, Q14676, Q3KNV8, Q07889, Q96EZ8, P08238, A5PL32, O75131, Q5SPY9, P49407, Q9UHB6, P34932, Q32P45, O43823, A0A024R1S8, O94979, Q9Y2W1, Q96JM3, Q9UKN8, Q9H0G5, A0A0A0MR66, O00264, O75122, Q8WVC0, Q9P2I0, E9PRY8, Q8IV36, Q5JWF2, P05387, P05388, B3KUY2, Q86VM9, Q13501, Q14157, Q02040, A8MXP9, A0A024RAN2, Q92597, P26232, A0A0A0MRM9, Q14247, Q66PJ3, Q9H0D6, Q96C24, E9PDH4, Q9GZT3, P21796, Q02156, P18615, Q9UNZ2, Q5JTD0, Q02410, B4DY08, Q9Y2X7, Q9NXG2, P15924, Q9BWW4, P49450, O43150, P17029, P35579, P35580, O95292, Q9BY44, P12694, O95365, O14545, Q13627, P46821, P07359, Q9BW71, Q9BRD0, Q86XP3, O95671, Q9H0B6, Q96KR1, Q9NYV4, A0A0A0MT33, Q9UKJ3, O14576, P01241, P35749, O14579, Q9NPJ4, Q9BWU0, P63027, Q8WXF7, Q96K21, P46100, P19634, Q7Z6Z7, Q86UE4, O96019, Q149N5, C9JA93, O60763, A0A0A0MS29, O75396, Q9H2P0, Q05519, O94921, Q14103, P35658

GO:0000166	nucleotide binding	46	8.1	7.7E-16	Q15287, P26378, Q13595, Q9NWH9, O95104, O75494, P45880, Q32P45, P31943, Q9GZT3, P18615, P23588, Q14498, P07900, A0A0A0MT33, Q9BZC7, Q15424, Q9P2K5, A0A0A0MR66, P05455, Q13283, Q16629, B4DY08, Q96T37, P51858, Q13242, P09651, Q9UPQ9, Q13247, P49756, Q96T58, E7EX17, Q96I25, P20020, J3KTL2, P52756, P07910, Q02040, Q9UKV3, Q9BXP5, Q08170, A0A024RDB2, A8MXP9, P53805, Q05519, Q14103, O43719, P43243, P19338, Q01130
GO:0098641	cadherin binding involved in cell-cell adhesion	40	7.0	2.0E-14	Q9UHB6, Q9UPN3, P18206, Q9H0B6, A0A087WUZ3, Q9UDY2, P35221, A0A0U4BW16, A0A024R1S8, Q9C0C2, P55196, Q6PKG0, Q09666, O60716, C9J6P4, P42166, A0A024R4E5, E9PRY8, Q9H4G0, Q9ULH1, P35611, Q9H2G2, P21333, Q07960, O00567, Q92522, P35579, Q13813, Q15149, O60763, P08238, O95292, Q9UQN3, Q9BY44, A0A024RAN2, Q16513, O76021, E7EX44, Q92597, P26232, Q14247
GO:0005200	structural constituent of cytoskeleton	21	3.7	2.3E-10	Q9UEY8, Q9H4G0, P15924, P07197, P16157, A0A087WUZ3, P11171, Q9Y4G6, P11277, Q01484, Q16352, Q13813, O43491, A0A0G2JMX7, O15061, Q12955, P08670, Q9Y2J2, P12036, O60504, P26232
GO:0005198	structural molecule activity	30	5.3	1.3E-09	Q9UEY8, P18206, P35606, P11137, P27816, P16157, P35221, P11171, Q9Y4G6, O43491, P48681, P08670, Q9Y2J2, Q9H307, Q13425, Q14980, O14579, P02671, Q9H4G0, P15924, P78559, P35612, P35611, P07197, P02675, Q3KQU3, P02545, A8MXP9, P12036, P43243, P46821
GO:0030507	spectrin binding	11	1.9	3.2E-09	Q12955, Q08AD1, P35612, Q08495, P35611, O14576, Q16849, P16157, P11171, Q01484, O43491
GO:0003723	RNA binding	44	7.7	4.2E-08	Q12906, Q15287, P26378, Q15477, O75494, L0R530, Q96FV9, P31943, P18615, P23588, Q14498, O60832, A0A0A0MT33, P05455, P49792, B4DY08, Q92945, Q9NXG2, Q00839, Q9P2I0, P09651, A0A024R4E5, P18583, Q13247, Q8TDD1, O95218, Q99502, E7EX17, Q96I25, P25490, Q59GJ0, O00567, P52948, J3KTL2, P52756, P07910, Q8N1G4, Q16637, Q53EL6, O75400, Q12996, Q14103, O76021, O43719, P19338, Q15459

GO:0003682	chromatin binding	34	6.0	3.3E-07	Q9Y3T9, P51531, P52701, Q92769, O75475, F8VXC8, Q8N7H5, P18615, Q13547, P83916, Q15424, Q04727, Q92922, Q7Z4V5, Q9UQE7, Q9P2D1, Q13131, Q02880, Q9H1E3, Q9Y618, P46100, Q5VTR2, Q6KC79, P49450, P17096, B4DR36, O96019, P05114, A0A140VKC4, P35659, Q9H2P0, C9JFV4, Q9HCD5, Q14103, Q5SSJ5
GO:0051015	actin filament binding	18	3.2	9.4E-07	Q9UHB6, Q9UPN3, Q0ZGT2, P35612, P35611, P28290, P21333, A0A0U4BW16, P35221, Q9Y4G6, A0A024R1S8, P11277, P35579, P35580, O75122, P29966, Q92614, P35749, P26232
GO:0005516	calmodulin binding	21	3.7	2.5E-06	P63027, Q9UEY8, Q08AD1, P49585, B7ZKY2, P35612, P23634, P35611, A0A087WUZ3, P11137, P19634, A0A0U4BW16, P20020, P49006, P11171, P35579, Q13813, P35580, P29966, Q13425, E7EX44, P35749
GO:0008017	microtubule binding	21	3.7	1.1E-05	D3DUW5, Q9NQT8, Q9UPN3, Q9UQ16, P78559, P07197, Q14839, P27816, P11137, L0R530, Q16555, A0A0G2JMX7, Q05193, O95292, P12036, O75122, O14576, Q9Y3E1, Q92597, P46821, Q14980
GO:0003779	actin binding	24	4.2	2.8E-05	Q9UEY8, Q9UPN3, P18206, A0A087WUZ3, A0A0U4BW16, P11171, P49006, Q9Y4G6, O43491, Q9Y2J2, Q13425, Q5T1M5, Q9H4G0, P35612, Q08495, Q8WX93, P35611, P11277, P35579, Q13813, P35580, Q15149, P17600, Q9HBL0, E7EX44
GO:0003729	mRNA binding	15	2.6	4.2E-05	Q9BWU0, P26378, P49756, Q32P45, O75533, J3KTL2, P52756, A0A140VKC4, P07900, Q9H0G5, P05455, Q13283, Q9BY44, A0A024RDB2, Q12996, A0A0A6YYJ8
GO:0031492	nucleosomal DNA binding	9	1.6	8.7E-05	Q13547, P05114, P07910, Q92769, Q14839, B4DY08, Q92922, P49450, O96019, F8VXC8
GO:0030674	protein binding, bridging	11	1.9	1.5E-04	P02671, Q16623, P15924, Q12955, P02675, P50502, P12036, P19634, Q9UDY2, Q01484, Q9UNE7
GO:0004386	helicase activity	11	1.9	3.5E-04	P51531, Q15477, Q86XP3, O75643, P46100, E7EX17, A0A024R0Z3, Q9GZR7, Q8N8A6, Q6P158, Q9P2D1, P23588
GO:0042826	histone deacetylase binding	12	2.1	3.9E-04	Q13547, A0A140VKC4, P07900, Q02880, Q9H7L9, Q9Y618, P08238, O75376, Q6KC79, O43823, Q14103, Q16513

GO:1990226	histone methyltransferase binding	4	0.7	5.9E-04	P83916, P08238, Q13185, O00567
GO:0044325	ion channel binding	12	2.1	9.2E-04	Q16623, P07900, Q99996, Q12955, P08238, P42858, P21333, Q14694, P49407, Q01484, P21796, P07948
GO:0004004	ATP-dependent RNA helicase activity	9	1.6	1.0E-03	Q15477, Q86XP3, Q13283, O75643, Q8TDD1, Q32P45, A0A024R0Z3, Q9GZR7, Q8N8A6, Q6P158
GO:0051020	GTPase binding	6	1.1	1.2E-03	P07900, P07814, Q13439, Q16849, P21333, A0A087WUZ3
GO:0003676	nucleic acid binding	49	8.6	2.0E-03	Q15287, Q9H0D6, Q9UBB9, O75494, P31943, Q9GZT3, P18615, Q15424, Q9P2K5, Q13283, Q16629, B4DY08, Q96T37, O75554, Q13242, P09651, P18583, Q8TDD1, P49756, Q96T58, P17029, Q7Z6E9, Q9UKV3, A0A024RDB2, P19338, Q9NWH9, Q13595, Q86XP3, Q32P45, Q96KR1, Q9HC78, Q96JM3, Q14498, A0A0A0MT33, Q9UKJ3, A0A0A0MR66, O75643, Q96I25, A0A024R0Z3, Q8N8A6, P25490, J3KTL2, P52756, P07910, Q08170, A8MXP9, Q9GZR7, Q05519, Q14103, P43243, O43719, Q01130
GO:0019901	protein kinase binding	24	4.2	2.1E-03	Q9NQT8, Q9UK58, Q9UEW8, Q8ND76, O94885, P18669, Q9UP95, Q13501, Q01484, Q15762, P21796, Q16555, A0A0G2JMX7, B7Z6G2, Q05193, P08238, P49023, Q8NFP9, O60716, P55011, P12036, P61266, P17600, P04792
GO:0017166	vinculin binding	4	0.7	3.2E-03	O15061, P49023, P35221, O60504
GO:0030506	ankyrin binding	5	0.9	3.2E-03	Q15149, A0A087WUZ3, Q9C0C2, P11277, P02730
GO:0008022	protein C-terminus binding	14	2.5	5.4E-03	Q13131, O94812, P55196, Q05193, Q02880, A0A024R2C5, O94885, P08670, Q15036, Q6KC79, Q14676, P19338, O00505, Q9UN37
GO:0045296	cadherin binding	5	0.9	5.5E-03	P18206, Q12955, O60716, P35221, Q92597
GO:0032564	dATP binding	3	0.5	5.8E-03	P07900, P08238, P50502
GO:0005484	SNAP receptor activity	6	1.1	7.4E-03	O00161, P63027, Q16623, O75396, P61266, O75379

GO:0030911	TPR domain binding	3	0.5	9.4E-03	P07900, P08238, Q9UNE7
GO:0019899	enzyme binding	20	3.5	1.0E-02	Q02880, Q92769, P17252, Q13185, P16157, P17096, Q9P035, Q9C0C2, Q01484, Q02156, Q13547, A0A0G2JMX7, Q9H7L9, P83916, Q9UKV3, O95292, P27708, Q9H4A6, Q9UNE7, P07948
GO:0005524	ATP binding	64	11.2	1.2E-02	P51531, Q9UEW8, Q15477, Q96T60, P52701, A0A0A0MS54, E9PDH4, A0A0U4BW16, Q02156, Q13283, Q9UQE7, Q00839, Q6P158, Q9P2D1, P15056, Q9NQT8, Q02880, P21127, Q9NTI5, B7ZKY2, P23634, Q8TDD1, Q9BTU6, Q9H2G2, O43237, P35579, Q9NTI2, P35580, P08238, P17600, O95747, Q13627, Q9C0C9, Q9Y6G9, P34932, Q86XP3, P17252, Q32P45, Q2M2I8, Q9Y2W1, Q9NYV4, P07900, Q9BZC7, P27708, P49840, P35749, Q53EW6, P07948, Q13131, Q8NE71, A0A024R2C5, P46100, O75643, Q14839, A0A024R0Z3, Q8N8A6, P20020, Q9Y2Q0, Q9UN37, Q13085, P07814, Q92614, Q9GZR7, O94921, Q16513, A0A0A0MRM9
GO:0005085	guanyl-nucleotide exchange factor activity	10	1.8	1.3E-02	B7Z6G2, Q6IAA8, Q13144, Q96N67, Q9Y6D5, Q8N5V2, Q149N5, Q07889, Q9NZN5, O15085
GO:0003713	transcription coactivator activity	16	2.8	1.3E-02	P51531, O75151, Q5VTR2, Q8NFD5, P25490, Q86UE4, O96019, F8VXC8, Q9Y2W1, H0YBE0, P53999, Q92539, O43294, Q16514, Q92922, P29375, P29590
GO:0016810	hydrolase activity, acting on carbon-nitrogen (but not peptide) bonds	4	0.7	1.3E-02	Q6DEN2, P27708, Q14194, Q16555
GO:0050733	RS domain binding	3	0.5	1.4E-02	P18583, O75494, J3KTL2
GO:0016887	ATPase activity	13	2.3	1.4E-02	Q9NQT8, Q9UPN3, P51531, A0A024R2C5, Q8NE71, P52701, A0A0U4BW16, Q9UN37, P35579, P07900, Q96T23, Q9BZC7, Q9UKV3, Q92614
GO:0005080	protein kinase C binding	6	1.1	1.5E-02	Q02880, P15924, P29966, Q13501, P04792, Q01130

GO:0005096	GTPase activator activity	17	3.0	1.7E-02	D3DUW5, Q9ULH1, Q4KMP7, O43150, Q9P035, Q07960, Q149N5, C9JA93, Q9NZN5, Q07889, Q8IWW6, Q6GYQ0, O43166, Q6ZRI8, Q9Y2X7, P49407, O15085
GO:0000980	RNA polymerase II distal enhancer sequence-specific DNA binding	7	1.2	1.7E-02	Q13547, P07910, Q92769, Q14839, B4DY08, Q92922, O96019, F8VXC8
GO:0003677	DNA binding	69	12.1	2.0E-02	Q12906, P23193, Q9UBB9, Q9NYF8, P82094, Q9NYB0, Q9P2K5, Q13283, Q8IXM2, Q9H307, P42166, Q92922, P08651, Q00839, Q02880, Q9NTI5, Q96ST2, P18583, Q5VZL5, Q96T58, P49450, Q15773, Q8NDX5, A0A0S2Z4Z6, Q07889, Q92522, Q9Y5B6, P05114, A0A140VKC4, Q96PU4, O95365, Q13207, Q496C9, Q8WYP5, O75376, Q32P45, O43823, Q8NFD5, Q96KR1, Q96FV9, F8VXC8, Q8TEA8, Q9HC78, Q9UKN8, Q12789, P53999, Q92945, P51858, P29375, Q7Z4V5, E9PRY8, Q08999, Q99996, Q9Y618, Q12888, P46100, Q14839, P17096, Q7Z6Z7, P25490, Q96KC8, P52756, P35659, Q9BXP5, Q9H2P0, P53805, Q16514, Q92614, Q13129, Q5SSJ5, P29590
GO:0002162	dystroglycan binding	3	0.5	3.1E-02	P18206, O75122, P11137
GO:0005522	profilin binding	3	0.5	3.1E-02	P42858, Q9Y6V0, Q14247
GO:0003756	protein disulfide isomerase activity	4	0.7	3.1E-02	Q15084, O00391, Q9H3N1, Q9H1E5
GO:0019900	kinase binding	7	1.2	3.2E-02	Q13131, P11166, Q16623, P08238, P42858, P21333, Q9UNE7
GO:0019904	protein domain specific binding	13	2.3	3.4E-02	P80723, P31323, Q13185, Q9UDY2, A0A0U4BW16, Q9UN37, Q8WWN9, P35579, Q16623, A0A140VKC4, Q9UQN3, P50502, P22059, P61266
GO:0048306	calcium-dependent protein binding	6	1.1	3.6E-02	D6REX3, P63027, Q16623, O75131, P17600, P19634, O94979
GO:0008134	transcription factor binding	16	2.8	3.8E-02	Q9H1E3, Q9Y618, Q92769, P35611, P21333, P17096, B4DR36, Q9Y5B6, Q13547, A0A140VKC4, P42858, C9JFV4, Q16514, Q16849, Q14103, P49407, O60504
GO:0003777	microtubule motor	7	1.2	4.1E-02	Q9NQT8, Q9Y6G9, Q9H0B6, O14576, O43237, Q9NSK0, Q9UQE7

	activity				
GO:0003690	double-stranded DNA binding	7	1.2	4.3E-02	Q96T60, Q9H1E3, Q9NWH9, Q15424, P52701, O43823, Q8ND56
GO:0003743	translation initiation factor activity	6	1.1	4.4E-02	Q13144, B5ME19, Q9BY44, O60841, E7EX17, Q59GJ0, P23588
